# The causal relationship between psoriasis and cancers: a two-sample Mendelian randomization analysis

**DOI:** 10.3389/fonc.2024.1366958

**Published:** 2024-03-21

**Authors:** Jiaye Long, Miyang Yang, Yingrong Pang, Hongyan Kang, Shuai Liang, Du Wang

**Affiliations:** ^1^ Department of Interventional Radiology, Inner Mongolia Forestry General Hospital, The Second Clinical Medical School of Inner Mongolia University for The Nationalities, Yakeshi, Inner Mongolia, China; ^2^ Department of Radiology, The First Clinical Medical College, Fujian University of Traditional Chinese Medicine, Fuzhou, Fujian, China; ^3^ Department of Cardiology, Inner Mongolia Forestry General Hospital, The Second Clinical Medical School of Inner Mongolia University for The Nationalities, Yakeshi, Inner Mongolia, China

**Keywords:** psoriasis, cancers, Mendelian randomization analysis, GWAS - genome-wide association study, meta - analysis

## Abstract

**Background:**

Although observational studies suggest a correlation between psoriasis (PS) and cancers, it is still unknown whether this association can replace causal relationships due to the limitations of observational studies. Therefore, we conducted a two-sample Mendelian randomization (MR) analysis to evaluate the causal relationship between PS and cancers.

**Methods:**

PS genetic summary data were obtained from two genome-wide association studies (GWAS). We employed MR Base for individuals retrieving tumors from distinct locations. Inverse-variance weighted analysis was the principal method used for MR, supplemented by weighted median, MR Egger, simple mode, and weighted mode. To investigate the possible link between psoriasis and cancers, we performed two independent two-sample MR studies and a meta-analysis based on two independent MR analyses.

**Results:**

Two independent MR analyses both found no significant causal relationship between PS and overall cancers (OR=1.0000, 95% confidence interval [CI]:0.9999-1.0001, *P*=0.984; OR=1.0000, 95% CI:0.9999-1.0001, *P*=0.761), and no significant causal relationship with 17 site-specific cancers. In the meta-analysis conducted by two two-sample MR analyses, there was no significant causal relationship between PS and overall cancers (OR=1.0000, 95% CI: 0.9999-1.0001, *P*=1.00, *I* ^2 =^ 0.0%), and there was no significant causal relationship with 17 site-specific cancers.

**Conclusions:**

Our findings do not support a genetic link between PS and cancers. More population-based and experimental investigations will be required better to understand the complicated relationship between PS and cancers.

## 1 Introduction

Psoriasis (PS) is an inflammatory disease that is controlled by several genes, influenced by environmental variables, and mediated by immunology (1). The prevalence of PS varies across regions worldwide, spanning from 0.5% to 4.6% (2). PS can affect the patient’s skin, scalp, joints, and other organs as a systemic disease. The following symptoms are the most commonly observed: The symptoms include (1) erythematous lesions (2); pruritus, stinging, or skin irritation (3); epidermal fissures, xerosis, and potential hemorrhage (4); flaky scalp; and (5) arthralgia and inflammation. The patient has been further burdened by comorbidities associated with PS, including cardiovascular disease, inflammatory gastrointestinal disease, diabetes, and PS arthritis (3). Notwithstanding this, the link between PS and cancer is uncertain, especially in tumors with specific locations. The significance of inflammatory factors in the pathogenesis of PS validates the soundness of this correlation. Previous research has also demonstrated that inflammatory factors increase tumor formation (4). A study based on the UK electronic medical record database investigated the link between PS and cancer, finding that it existed only in non-melanoma skin cancer, lymphoma, and lung cancer (5). A meta-analysis also indicates that PS is linked to cancers (6).

Still, observational research has its drawbacks, including confounding bias, information bias, and the challenge of establishing causal relationships. The reported correlation between PS and cancer in observational studies could be coincidental. Therefore, this relationship requires additional investigation. As a statistical model, mendelian randomization (MR) employs genetic variation as instrumental variables (IVs) to infer the causal relationship between exposure and outcome (7, 8). Genetic variation is intrinsic and assigned randomly after conception, simulating a randomized controlled environment, reducing the impact of reverse causality, and explaining confounding circumstances. Therefore, MR has been frequently used for causal analysis of exposure and outcomes. This study aims to employ a MR analysis to evaluate whether gene-determined PS has a causal association with cancer at specific sites.

## 2 Materials and methods

### 2.1 Study design

**Figure 1:**
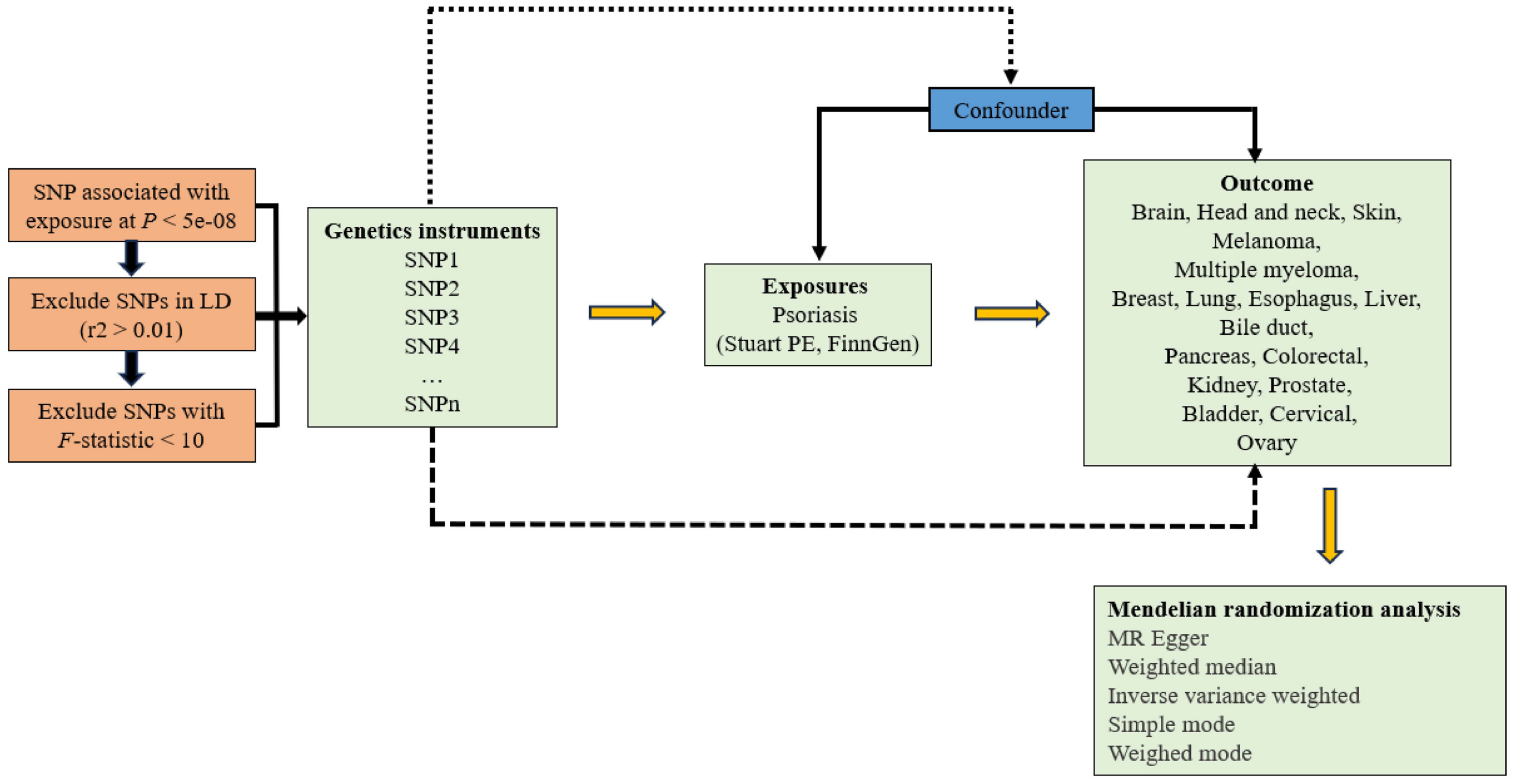
Study design of Mendelian randomization study on the causal relationship between psoriasis and cancers.

The design of the study is shown in **Figure 1**.

### 2.2 Selection of data sources and candidate instrumental variables

We obtained PS GWAS summary data via the IEU Open GWAS Project online platform from Stuart PE et al. (PMID: 34927100; GWAS ID: ebi-a-GCST90019016) and FinnGen (GWAS ID: Finn-b-L12-PSORIASIS). Stuart PE et al. included eight cohorts (15,967 cases and 28,194 controls) and conducted a meta-analysis of 8,536,277 markers in 4 cohorts using inverse variance weighting (IVW) method, resulting in 47 loci. By accumulating genetic, clinical, and health data, the FinnGen project, a large-scale genetic research initiative, investigates the correlation between genomic information and health characteristics of the Finnish population. In the final analysis, ICD-10 code L40 incorporated 4,510 cases and 212,242 controls for 16,380,464 loci.

We utilized MR Base (http://app.mrbase.org), a novel platform that aggregated data from multiple GWAS in a unified fashion, to analyze cancers (9). In order to obtain the outcome data of tumors at specific locations, we sequentially input “esophagus,” “multiple myeloma,” “liver,” “pancreas,” “biliary tract,” “cancer,” “coloratum,” “breast,” “kidney,” “lung,” “melanoma,” “state,” “brain,” “head and neck,” “leukemia,” “oval,” “bladder,” “skin” to obtain the corresponding GWAS ID. Concurrently, to perform a two-sample MR analysis, we excluded populations from the same source as those exposure revealed by accessing the UK Biobank database.

Use a filtering condition of p < 5e-08 to identify single nucleotide polymorphisms (SNPs) that are substantially connected with exposure factors as IVs when performing correlation analysis on exposure data. Subsequently, adjust the clump parameter (r2 > 0.01, distance=10,000kb) to eliminate chain imbalance interference. In addition, SNPs with incompatible alleles and palindromic SNPs were removed from MR analysis. Other populations will not be included in our MR investigation, which picked members of the European population.

### 2.3 Statistical analysis

The primary analysis method for MR was IVW (10), with supplementary approaches including MR Egger (11), weighted median (12), simple mode (13), and weighted mode (14). We tested the IVs strength using the F-statistic to remove the impact of weak instrumental bias on estimating causal relationships (15). F=R^2^ (N-K-1)/K (1-R^2^) was the formula used to calculate the F-statistic, where R^2^, N, and K represented the proportion of exposure variance explained by genetic variation, sample size, and number of IVs, respectively. F > 10 indicated the absence of weak instrumental bias. MR analysis results may be impacted by heterogeneity when the IVs for exposure and outcome were derived from various analysis platforms, studies, and populations. The IVW and MR Egger tests were used to assess heterogeneity, and a P-value < 0.05 suggested that heterogeneity existed in the study. In order to investigate the influence of remaining SNPs on causal connections, sensitivity analysis was performed using the leave-one-out method, which involved removing one SNP linked to exposure at a time. Outlier SNPs with possible pleiotropy bias can be found using MR-PRESSO; if the P-value < 0.05, outliers were present (16). Prior to performing MR analysis, eliminate any outliers. We used the Bonferroni correction, where a P-value < 0.0015 (0.05/(2 PS exposures * 17 tumor outcomes)) was statistically significant, taking into account the number of tumors to prevent false positives in multiple tests. Since our study’s exposure sources came from two distinct databases, we performed a meta-analysis to determine the causal relationship between each exposure and outcome. When *I* ^2^ > 50%, a random effect model was applied since it was deemed to have high heterogeneity. Conversely, a fixed effect model was applied. All results are represented by odds ratio (OR) values and 95% confidence intervals (CI). For all statistical studies, R software (version 4.3.0) was used. The R software packages “TwoSampleMR,” “Mendelian Randomization,” “MR PRESSO,” and “meta” were among those that were utilized.

## 3 Results

The number of SNPs selected from the PS GWAS of Stuart PE and FinnGen was shown in **Table 1**; **Supplementary Tables 1**-**34**. All F-statistics were greater than 10 (**Supplementary Tables 1**-**34**). The Cochran heterogeneity test using MR Egger and IVW methods showed that 8 out of 34 MR analyses exhibited heterogeneity (**Supplementary Table 35**). MR-Egger test showed no statistically significant difference in their intercepts (*P* > 0.05), indicating that our study did not observe directed pleiotropy (**Supplementary Table 35**). Meanwhile, MR-PRESSO did not detect outliers. The results of the leave-one-out method indicated that the SNPs removed one by one did not impact the results (**Supplementary Figure 1**; **Supplementary Figure 2**).

**Table 1:** Results of Mendelian randomization analysis and meta-analysis using inverse-variance weighted method for psoriasis and cancers.

exposure	outcome	nsnp	OR (95% CI)	pval	*I* ^2^ (%)
Stuart PE FinnGen Meta-analysis	brain	48	1.0001 (0.9999-1.0002)	0.488	
		12	1.0000 (0.9998-1.0003	0.966	
			1.0001 (1.0000-1.0002)	0.263	0.0
Stuart PE FinnGen Meta-analysis	head and neck	49	1.0003 (1.0000-1.0005)	0.025	
		12	1.0004 (1.0000-1.0007)	0.027	
			1.0001 (0.9995-1.0006)	0.826	69.4
Stuart PE FinnGen Meta-analysis	skin	47	1.0002 (0.9995-1.0009)	0.504	
		10	0.9999 (0.9992-1.0005)	0.694	
			1.0000 (0.9996-1.0005)	0.873	0.0
Stuart PE FinnGen Meta-analysis	melanoma	25	0.9997 (0.9994-1.0000)	0.051	
		2	0.9992 (0.9982-1.0002)	0.106	
			0.9994 (0.9985-1.0002)	0.128	0.0
Stuart PE FinnGen Meta-analysis	multiple myeloma	48	1.0000 (0.9999-1.0002)	0.649	
		12	1.0000 (0.9998-1.0003)	0.768	
			1.0000 (0.9999-1.0000)	1.000	0.0
Stuart PE FinnGen Meta-analysis	breast	47	0.9743 (0.9484-1.0008)	0.057	
		13	1.0140 (0.9974-1.0307)	0.100	
			0.9954 (0.9574-1.0349)	0.816	83.7
Stuart PE FinnGen Meta-analysis	lung	49	1.0003 (0.9998-1.0008)	0.226	
		12	1.0001 (0.9994-1.0009)	0.688	
			1.0003 (0.9999-1.0007)	0.205	0.0
Stuart PE FinnGen Meta-analysis	esophagus	48	1.0001 (0.9999-1.0003)	0.353	
		12	0.9999 (0.9997-1.0003)	0.963	
			1.0001 (0.9997-1.0003)	0.415	0.0
Stuart PE FinnGen	liver	44	0.9999 (0.9998-1.0000)	0.126	
		11	1.0000 (0.9998-1.0001)	0.497	
Meta-analysis			0.9999 (0.9998-1.0000)	0.103	15.4
Stuart PE FinnGen Meta-analysis	bile duct	48	1.0000 (0.9998-1.0001)	0.669	
		12	1.0000 (0.9998-1.0002)	0.740	
			1.0000 (0.9999-1.0001)	1.000	0.0
Stuart PE FinnGen Meta-analysis	pancreas	22	1.0482 (0.9084-1.2100)	0.519	
		4	0.9300 (0.6548-1.3180)	0.680	
			1.0302 (0.9022-1.1763)	0.661	0.0
Stuart PE FinnGen Meta-analysis	colorectum	49	1.0003 (1.0000-1.0010)	0.405	
		12	1.0003 (0.9994-1.0011)	0.525	
			1.0003 (0.9999-1.0007)	0.172	0.0
Stuart PE FinnGen Meta-analysis	kidney	26	1.0000 (0.9997-1.0002)	0.770	
		2	1.0001 (0.9989-1.0013)	0.864	
			1.0000 (0.9998-1.0002)	0.973	0.0
Stuart PE FinnGen Meta-analysis	prostate	49	0.9982 (0.9968-0.9997)	0.016	
		12	0.9980 (0.9957-1.0004)	0.105	
			0.9981 (0.9969-0.9994)	0.003	0.0
Stuart PE FinnGen Meta-analysis	bladder	49	1.0001 (0.9998-1.0004)	0.632	
		12	1.0000 (0.9997-1.0003)	0.899	
			1.0001 (0.9998-1.0003)	0.620	0.0
Stuart PE FinnGen Meta-analysis	cervix	48	0.9998 (0.9994-1.0001)	0.280	
		12	1.0002 (0.9998-1.0006)	0.340	
			1.0000 (0.9996-1.0004)	0.951	54.0
Stuart PE FinnGen Meta-analysis	ovary	49	1.0000 (0.9995-1.0005)	0.991	
		12	0.9997 (0.9991-1.0003)	0.636	
			0.9999 (0.9995-1.0003)	0.531	0.0
Stuart PE FinnGen Meta-analysis	overall		1.0000 (0.9999-1.0001) 1.0000 (0.9999-1.0001) 1.0000 (0.9999-1.0001)	0.984 0.761 1.000	0.0

Based on PS GWAS of Stuart PE, there was no causal relationship between genetically predicted PS and brain cancer (OR=1.0001, 95% CI: 0.9999-1.0002, *P*=0.488), head and neck cancer (OR=1.0003, 95% CI: 1.0000-1.0005, *P*=0.025), skin cancer (OR=1.0002, 95% CI: 0.9995-1.0009, *P*=0.504), melanoma (OR=0.9997, 95% CI: 0.9994-1.0000, *P*=0.051), multiple myeloma (OR=1.0000, 95% CI: 0.9999-1.0002, *P*=0.649), breast cancer (OR=0.9743, 95% CI: 0.9484-1.0008, *P*=0.057), lung cancer (OR=1.0003, 95% CI: 0.9998-1.0008, *P*=0.226), esophageal cancer (OR=1.0001, 95% CI: 0.9999-1.0003, *P*=0.353), liver cancer (OR=0.9999, 95% CI: 0.9998-1.0000, *P*=0.126), bile duct cancer (OR=1.0000, 95% CI: 0.9998-1.0001, *P*=0.669), pancreatic cancer (OR=1.0482, 95% CI: 0.9084-1.2100, *P*=0.519), colorectal cancer (OR=1.0003, 95% CI: 1.0000-1.0010, *P*=0.405), kidney cancer (OR=1.0000, 95% CI: 0.9997-1.0002, *P*=0.770), prostate cancer (OR=0.9982, 95% CI: 0.9968-0.9997, *P*=0.016), bladder cancer (OR=1.0001, 95% CI: 0.9998-1.0004, *P*=0.632), cervical cancer (OR=0.9998, 95% CI: 0.9994-1.0001, *P*=0.280), ovarian cancer (OR=1.0000, 95% CI: 0.9995-1.0005, *P*=0.991), and overall cancer (OR=1.0000, 95% CI: 0.9999-1.0001, *P*=0.984) (**Table 1**).

Furthermore, based on PS GWAS of FinnGen, there was no causal relationship between genetically predicted PS and brain cancer (OR=1.0000, 95% CI: 0.9998-1.0003, *P*=0.966), head and neck cancer (OR=1.0004, 95% CI: 1.0000-1.0007, *P*=0.027), skin cancer (OR=0.9999, 95% CI: 0.9992-1.0005, *P*=0.694), melanoma (OR=0.9992, 95% CI: 0.9982-1.0002, *P*=0.106), multiple myeloma (OR=1.0000, 95% CI: 0.9998-1.0003, *P*=0.768), breast cancer (OR=1.0140, 95% CI: 0.9974-1.0307, *P*=0.100), lung cancer (OR=1.0001, 95% CI: 0.9994-1.0009, *P*=0.688), esophageal cancer (OR=0.9999, 95% CI: 0.9997-1.0003, *P*=0.963), liver cancer (OR=1.0000, 95% CI: 0.9998-1.0001, *P*=0.497), bile duct cancer (OR=1.0000, 95% CI: 0.9998-1.0002, *P*=0.740), pancreatic cancer (OR=0.9300, 95% CI: 0.6548-1.3180, *P*=0.680), colorectal cancer (OR=1.0003, 95% CI: 0.9994-1.0011, *P*=0.525), kidney cancer (OR=1.0001, 95% CI: 0.9989-1.0013, *P*=0.864), prostate cancer (OR=0.9980, 95% CI: 0.9957-1.0004, *P*=0.105), bladder cancer (OR=1.0000, 95% CI: 0.9997-1.0003, *P*=0.899), cervical cancer (OR=1.0002, 95% CI: 0.9998-1.0006, *P*=0.340), ovarian cancer (OR=0.9997, 95% CI: 0.9991-1.0003, *P*=0.636), and overall cancer (OR=1.0000, 95% CI: 0.9999-1.0001, *P*=0.761) (**Table 1**).

Eventually, we performed a meta-analysis of the OR values and their CI generated by Stuart PE and FinnGen’s GWAS to generate new OR values and 95% CI. The meta-analysis had no significant causal relationship between PS and brain cancer (OR=1.0001, 95% CI: 1.0000-1.0002, *P*=0.263), head and neck cancer (OR=1.0001, 95% CI: 0.9995-1.0006, *P*=0.826), skin cancer (OR=1.0000, 95% CI: 0.9996-1.0005, *P*=0.873), melanoma (OR=0.9994, 95% CI: 0.9985-1.0002, *P*=0.128), multiple myeloma (OR=1.0000, 95% CI: 0.9999-1.0000, *P*=1.000), breast cancer (OR=0.9954, 95% CI: 0.9574-1.0349, *P*=0.816), lung cancer (OR=1.0003, 95% CI: 0.9999-1.0007, *P*=0.205), esophageal cancer (OR=1.0001, 95% CI: 0.9997-1.0003, *P*=0.415), liver cancer (OR=0.9999, 95% CI: 0.9998-1.0000, *P*=0.103), bile duct cancer (OR=1.0000, 95% CI: 0.9999-1.0001, *P*=1.000), pancreatic cancer (OR=1.0302, 95% CI: 0.9022-1.1763, *P*=0.661), colorectal cancer (OR=1.0003, 95% CI: 0.9999-1.0007, *P*=0.172), kidney cancer (OR=1.0000, 95% CI: 0.9998-1.0002, *P*=0.973), prostate cancer (OR=0.9981, 95% CI: 0.9969-0.9994, *P*=0.003), bladder cancer (OR=1.0001, 95% CI: 0.9998-1.0003, *P*=0.620), cervical cancer (OR=1.0000, 95% CI: 0.9996-1.0004, *P*=0.951), ovarian cancer (OR=0.9999, 95% CI: 0.9995-1.0003, *P*=0.531), and overall cancer (OR=1.0000, 95% CI: 0.9999-1.0001, *P*=1.000) (**Table 1**). The results of the other four methods were consistent with those of the IVW method (**Supplementary Table 36**).

## 4 Discussion

We discovered no significant causal connection between PS and 17 site-specific cancers in our two-sample MR analysis. This discovery was confirmed by sensitivity analysis and was consistent across two independent exposure data sources. The meta-analysis conducted on both data sets yielded consistent results. To our knowledge, this was the first MR study to look into the link between PS and 17 site-specific cancers.

Numerous epidemiological studies have already been conducted on the association between PS and cancers. The standardized incidence rate of malignant tumors was 1.40 (95% CI: 1.21-1.51) in a cohort study undertaken by Frentz et al. (17) over a mean follow-up period of 9.3 years after the discharge of patients with PS. Simultaneously, Ji et al. (18) concluded that the standardized incidence rate of total cancers in PS patients throughout the average 10-year follow-up period after discharge was 1.33 (95% CI: 1.26-1.40). Although observational studies in clinical practice indicate the link between PS and cancers, the underlying processes are unknown. Furthermore, it is currently unknown if correlations found in observational research may be used to replace causal links. This problem can be effectively solved via MR analysis.

Our findings imply that PS and tumors are not causally related, indicating that observational studies that have already been published may have been skewed by confounding variables. According to the most recent epidemiological research, obesity, alcohol use, and smoking are among the risk factors for PS (19). Indeed, several cancers, including pancreatic, liver, esophageal, and lung cancers, are also linked to obesity, drinking, and smoking (20–22). Although there are overlaps in the risk variables for cancers and PS, these confounding factors have not been considered in much prior epidemiological research. Thus, specific epidemiological research implies that PS is connected with a higher risk of acquiring cancers, which the common risk factors between PS and cancers can explain. Nevertheless, very little data on the association between PS and cancers has been adjusted for confounding variables. When smoking, BMI, age, education level, and hormone use were taken into account, the study’s findings revealed that PS was solely linked to the risk of colon cancer (HR=1.6, 95% CI: 1.0-2.4), not the chance of acquiring other types of cancers (23). Concurrently, a different study that controlled for several factors (such as age, physical activity, BMI, smoking, drinking, hypertension, and diabetes) found a correlation between PS and the risk of gastric cancer (HR=1.31, 95% CI: 1.08-1.58), but not with other cancer risks (24). Murdaca et al. (25) investigated the expression of human leukocyte antigen-G (HLA-G) in gastric cancer tissue and discovered that HLA-G was present in gastric adenocarcinoma but not in the non-tumor gastric mucosa. Under normal conditions, HLA-G is highly expressed in the trophoblast cells of the human placenta and can also be found in early pregnancy fetal brain, thymus, and adult eye tissue, indicating a selective tissue distribution (26). HLA-G promotes immunological tolerance by controlling the activity of natural killer (NK) cells, T cells, and antigen-presenting cells (27, 28). As a result, HLA-G may play a role in PS, an inflammatory disease that affects certain organs. At the same time, HLA-G molecules allow tumor cells to elude the killing and dissolving actions of NK cells and cytotoxic T cells, which is a strategy for tumor cells to avoid immune surveillance. However, there is currently a lack of study on the association between HLA-G and cancers, with the exception of gastric cancer. More research is needed to determine the association between HLA-G and cancers.

However, we cannot neglect that the majority of research on the link between PS and cancer is focused on hospitalized patients. Furthermore, the PS that landed these people in the hospital could have been more severe. Nonetheless, most research does not stratify PS severity but focuses on the relationship between PS and cancers. Regardless, the impact of PS severity classifications on tumor risk may vary. According to research, the risk of developing cancer increases in direct proportion to the severity of PS (29, 30).

The mechanism underlying PS and tumor development is currently being researched. However, the inflammatory response of the two pathways is the most fiercely debated. PS is caused by activated plasma cell dendritic cells producing pro-inflammatory cytokines and tumor necrosis factor-α (TNF-α), activating myeloid dendritic cells (31). Activated myeloid dendritic cells also produce IL-12 and IL-23, which activate Th1 and Th17 (31). Keratinocytes are triggered by the release of tumor necrosis factor-a and IL-17A by both Th1 and Th17 (3). Keratinocytes then excite plasma cell dendritic cells, which generate a variety of cytokines, chemokines, and antimicrobial peptides (3). As a result, the inflammatory response lasts an extended period. TNF-α, IL-1, IL-12, IL-23, and other upregulated cytokines in PS have been demonstrated to play a role in some malignancies (32). Prospective studies are required, and common confounding factors must be thoroughly explored in order to truly and honestly study the impact of PS on malignancies. MR analysis, as a research approach in evidence-based medicine with the second most vigorous justification after randomized controlled trials, can be a substitute to some extent.

Our study offers some advantages. For starters, MR can reduce the impact of confounding factors and reverse causation in inferring causal correlations, making it superior to typical observational investigations. Furthermore, we chose two extensive GWAS databases for the IVs of PS to ensure the richness and applicability of genetic variables. Likewise, the sensitivity analysis and the subsequent meta-analysis showed consistent results when investigating the causal association between PS and cancers. Lastly, the genetic variables we chose are all from the European population, eliminating redundant bias. Some limits, however, cannot be overlooked. To begin, the GWAS database simply includes the PS exposure level without considering the severity of PS or treatment alternatives. Second, because all of our genetic data is from Europe, its extrapolation is limited.

In conclusion, our findings imply no link between PS and 17 site-specific cancers. However, due to the potential confounding factors identified in clinical practice, more rigorous tests must be designed in the future to clarify the complex association between PS and cancers.

## Data availability statement

The original contributions presented in the study are included in the article/**Supplementary Material**. Further inquiries can be directed to the corresponding author.

## Author contributions

JL: Writing – original draft, Writing – review & editing. MY: Conceptualization, Writing – review & editing. YP: Conceptualization, Writing – review & editing. HK: Data curation, Writing – review & editing. SL: Data curation, Writing – review & editing. DW: Writing – review & editing, Supervision.
