# Supplementary material for: The causal relationship between psoriasis and cancers: a two-sample Mendelian randomization analysis
Source: Front Oncol. 2024 Mar 21;14:1366958. doi: 10.3389/fonc.2024.1366958 (PMC10991695; doi:10.3389/fonc.2024.1366958)

Supplementary Material

Supplementary Table 1. Genetic instrumental tools used in Mendelian randomization analysis on psoriasis and brain cancer, based on Stuart PE et al.

| SNP | Effect allele | Other allele | Chr | Pos | Exposure | | | Outcome | | | R^2^ | F |
| --- | --- | --- | --- | --- | --- | --- | --- | --- | --- | --- | --- | --- |
|  |  |  |  |  | beta | se | pval | beta | se | pval |  |  |
| rs10888503 | T | C | 1 | 1.53E+08 | 0.1866 | 0.0171 | 1.44E-27 | 9.80E-05 | 9.87E-05 | 0.32 | 0.015673 | 109.8526 |
| rs10893884 | C | T | 11 | 1.28E+08 | -0.106 | 0.016 | 3.99E-11 | -7.92E-05 | 9.37E-05 | 0.4 | 0.005614 | 38.95497 |
| rs11085744 | T | C | 19 | 10819967 | 0.1146 | 0.0166 | 5.21E-12 | -0.0002 | 9.45E-05 | 0.038 | 0.006476 | 44.96898 |
| rs1108618 | G | A | 10 | 81043743 | -0.1098 | 0.0165 | 3.25E-11 | -0.00013 | 9.44E-05 | 0.18 | 0.005911 | 41.02386 |
| rs11135059 | A | G | 5 | 1.59E+08 | -0.3078 | 0.0178 | 4.41E-67 | -2.45E-05 | 9.95E-05 | 0.81 | 0.041839 | 301.2692 |
| rs11249215 | A | G | 1 | 25297184 | 0.1403 | 0.016 | 2.14E-18 | 5.93E-06 | 9.40E-05 | 0.95 | 0.009815 | 68.38812 |
| rs11581607 | A | G | 1 | 67707690 | -0.3647 | 0.036 | 4.56E-24 | -0.00011 | 0.000187 | 0.55 | 0.016582 | 116.3357 |
| rs11767350 | G | A | 7 | 37385365 | -0.0998 | 0.0164 | 1.19E-09 | -1.75E-05 | 9.44E-05 | 0.85 | 0.00487 | 33.76492 |
| rs11795343 | C | T | 9 | 32523737 | -0.1062 | 0.0164 | 1.02E-10 | -9.80E-05 | 9.56E-05 | 0.31 | 0.00542 | 37.59828 |
| rs118002009 | T | G | 11 | 64124980 | -0.102 | 0.0169 | 1.62E-09 | -0.00013 | 9.39E-05 | 0.17 | 0.005145 | 35.68061 |
| rs12133684 | A | G | 1 | 2.07E+08 | 0.1218 | 0.0206 | 3.26E-09 | -0.00014 | 0.000118 | 0.23 | 0.004753 | 32.95205 |
| rs12188300 | T | A | 5 | 1.59E+08 | 0.5005 | 0.0264 | 5.39E-80 | 9.11E-05 | 0.00016 | 0.57 | 0.042431 | 305.7206 |
| rs12211087 | A | T | 6 | 31269946 | 1.3769 | 0.0253 | 1E-200 | -1.14E-05 | 0.000163 | 0.94 | 0.312188 | 3131.528 |
| rs1295685 | G | A | 5 | 1.32E+08 | 0.1759 | 0.0206 | 1.16E-17 | -5.21E-05 | 0.000121 | 0.67 | 0.009166 | 63.82789 |
| rs131656 | A | G | 22 | 21917450 | 0.114 | 0.0202 | 1.69E-08 | 0.000179 | 0.000119 | 0.13 | 0.004028 | 27.9037 |
| rs142502677 | A | T | 18 | 51791307 | 0.1035 | 0.0185 | 2.22E-08 | 0.000125 | 0.000107 | 0.24 | 0.004251 | 29.45142 |
| rs1611236 | A | G | 6 | 29748690 | 0.123 | 0.0172 | 7.90E-13 | 6.79E-05 | 9.99E-05 | 0.5 | 0.006625 | 46.0133 |
| rs1648153 | A | G | 11 | 1.1E+08 | -0.1388 | 0.0164 | 2.93E-17 | 5.55E-05 | 9.44E-05 | 0.56 | 0.009405 | 65.5077 |
| rs2021511 | T | C | 16 | 11344903 | -0.1163 | 0.0181 | 1.20E-10 | 0.000109 | 0.000104 | 0.3 | 0.005427 | 37.64747 |
| rs2066819 | T | C | 12 | 56750204 | -0.3283 | 0.0348 | 4.28E-21 | -6.88E-05 | 0.000188 | 0.71 | 0.013291 | 92.93557 |
| rs2111485 | G | A | 2 | 1.63E+08 | 0.1616 | 0.0166 | 1.76E-22 | -4.81E-05 | 9.55E-05 | 0.61 | 0.012453 | 86.99818 |
| rs2451258 | T | C | 6 | 1.6E+08 | -0.0985 | 0.0169 | 5.26E-09 | -0.00012 | 9.67E-05 | 0.21 | 0.004536 | 31.43511 |
| rs2638281 | G | A | 19 | 49210869 | 0.0976 | 0.0164 | 2.46E-09 | 0.000101 | 9.55E-05 | 0.29 | 0.004561 | 31.61174 |
| rs2675662 | G | A | 10 | 75599127 | -0.1184 | 0.0167 | 1.49E-12 | -6.05E-05 | 9.42E-05 | 0.52 | 0.006894 | 47.89755 |
| rs2735009 | A | G | 6 | 29807853 | -0.2952 | 0.0204 | 1.63E-47 | -4.40E-05 | 0.000115 | 0.7 | 0.028588 | 203.045 |
| rs28510484 | C | G | 15 | 31637569 | -0.1398 | 0.0231 | 1.37E-09 | -0.00013 | 0.000125 | 0.29 | 0.005515 | 38.26397 |
| rs28752856 | G | C | 6 | 31298421 | 1.1163 | 0.0225 | 1E-200 | -1.98E-05 | 0.000147 | 0.89 | 0.260118 | 2425.599 |
| rs28998802 | A | G | 17 | 26124908 | 0.2113 | 0.0222 | 1.89E-21 | -1.42E-05 | 0.000136 | 0.92 | 0.010792 | 75.26839 |
| rs34536443 | C | G | 19 | 10463118 | -0.6799 | 0.0504 | 2.02E-41 | -0.00034 | 0.000232 | 0.14 | 0.039812 | 286.07 |
| rs35741374 | T | C | 2 | 61072567 | 0.1634 | 0.0163 | 1.08E-23 | -0.00014 | 9.44E-05 | 0.15 | 0.013092 | 91.52266 |
| rs3906814 | G | C | 3 | 17011474 | -0.1144 | 0.0161 | 1.30E-12 | 0.000197 | 9.35E-05 | 0.035 | 0.006543 | 45.43733 |
| rs39841 | A | G | 5 | 96120170 | -0.1624 | 0.0176 | 2.56E-20 | 3.75E-05 | 0.000105 | 0.72 | 0.010447 | 72.84102 |
| rs438650 | C | T | 1 | 8290082 | 0.1244 | 0.0195 | 1.76E-10 | 2.69E-05 | 0.000112 | 0.81 | 0.005463 | 37.89548 |
| rs4712520 | C | T | 6 | 20640871 | 0.1301 | 0.0212 | 8.27E-10 | 0.000163 | 0.000121 | 0.18 | 0.005063 | 35.11242 |
| rs4889526 | A | C | 16 | 31030344 | 0.1216 | 0.0165 | 1.62E-13 | -0.00013 | 9.66E-05 | 0.17 | 0.006924 | 48.10189 |
| rs4978343 | T | G | 9 | 1.11E+08 | -0.0988 | 0.0164 | 1.77E-09 | 9.96E-05 | 9.54E-05 | 0.3 | 0.00472 | 32.72265 |
| rs55868394 | A | C | 17 | 73851113 | 0.1389 | 0.0246 | 1.71E-08 | 0.000199 | 0.000142 | 0.16 | 0.004257 | 29.49502 |
| rs559406 | T | G | 18 | 12857002 | -0.0924 | 0.0161 | 1.02E-08 | -7.08E-06 | 9.38E-05 | 0.94 | 0.004232 | 29.3228 |
| rs582757 | T | C | 6 | 1.38E+08 | -0.1846 | 0.0176 | 1.15E-25 | -7.81E-05 | 0.000105 | 0.46 | 0.013563 | 94.86574 |
| rs59960858 | A | C | 1 | 24519437 | -0.2086 | 0.0243 | 1.06E-17 | 9.54E-05 | 0.000137 | 0.49 | 0.010129 | 70.59714 |
| rs6894840 | G | T | 5 | 1.59E+08 | 0.1152 | 0.0166 | 3.42E-12 | 6.33E-05 | 9.63E-05 | 0.51 | 0.00627 | 43.53191 |
| rs7141014 | C | T | 14 | 98667928 | -0.1123 | 0.0203 | 3.07E-08 | 3.14E-05 | 0.000115 | 0.79 | 0.004173 | 28.91037 |
| rs73986523 | C | T | 17 | 40302271 | -0.2122 | 0.038 | 2.41E-08 | -0.00012 | 0.000195 | 0.54 | 0.005164 | 35.81192 |
| rs771576 | C | T | 3 | 1.02E+08 | 0.1029 | 0.0169 | 1.08E-09 | 4.28E-05 | 9.63E-05 | 0.66 | 0.005001 | 34.67425 |
| rs8016947 | G | T | 14 | 35832666 | 0.1476 | 0.0162 | 8.18E-20 | 0.00013749 | 9.41E-05 | 0.36 | 0.010726 | 74.80533 |
| rs9258357 | C | T | 6 | 29751129 | 0.2839 | 0.0233 | 3.48E-34 | -2.01E-05 | 0.000127 | 0.87 | 0.021924 | 154.654 |
| rs9264277 | C | T | 6 | 31224667 | 0.3705 | 0.018 | 1.91E-94 | 3.47E-06 | 9.71E-05 | 0.97 | 0.063721 | 469.5559 |
| rs9277939 | A | C | 6 | 33189873 | 0.228 | 0.0253 | 2.16E-19 | 0.000181 | 0.000159 | 0.26 | 0.009052 | 63.0248 |
| rs9468618 | T | C | 6 | 29750776 | -0.1856 | 0.03 | 6.49E-10 | 0.000106 | 0.000164 | 0.52 | 0.005617 | 38.97465 |
| rs9481169 | T | G | 6 | 1.12E+08 | 0.3716 | 0.0263 | 2.47E-45 | -0.00018 | 0.000167 | 0.27 | 0.021616 | 152.4297 |
| rs9513593 | A | G | 13 | 99950260 | -0.1197 | 0.0205 | 5.33E-09 | -0.00011 | 0.000125 | 0.39 | 0.004004 | 27.73422 |
| rs957970 | G | A | 17 | 40519890 | 0.0929 | 0.0168 | 3.05E-08 | 8.95E-05 | 9.79E-05 | 0.36 | 0.003925 | 27.18707 |
| rs9591325 | C | T | 13 | 50811220 | -0.19 | 0.0324 | 4.50E-09 | -0.0002 | 0.000186 | 0.29 | 0.004575 | 31.71194 |
| rs9695923 | T | C | 9 | 1.11E+08 | -0.0955 | 0.0165 | 7.08E-09 | 0.000137 | 9.55E-05 | 0.15 | 0.004389 | 30.41522 |

Supplementary Table 2. Genetic instrumental tools used in Mendelian randomization analysis on psoriasis and head and neck cancer, based on Stuart PE et al.

| SNP | Effect allele | Other allele | chr | pos | Exposure | | | Outcome | | | R^2^ | F |
| --- | --- | --- | --- | --- | --- | --- | --- | --- | --- | --- | --- | --- |
|  |  |  |  |  | beta | se | pval | beta | se | pval |  |  |
| rs10888503 | T | C | 1 | 1.53E+08 | 0.1866 | 0.0171 | 1.44E-27 | 1.33E-05 | 0.000133 | 0.92 | 0.015673 | 108.0024 |
| rs10893884 | C | T | 11 | 1.28E+08 | -0.106 | 0.016 | 3.99E-11 | -4.03E-05 | 0.000126 | 0.75 | 0.005614 | 38.29788 |
| rs11085744 | T | C | 19 | 10819967 | 0.1146 | 0.0166 | 5.21E-12 | -3.45E-05 | 0.000127 | 0.79 | 0.006475 | 44.20969 |
| rs1108618 | G | A | 10 | 81043743 | -0.1098 | 0.0165 | 3.25E-11 | 0.000232 | 0.000127 | 0.068 | 0.005911 | 40.33407 |
| rs11135059 | A | G | 5 | 1.59E+08 | -0.3078 | 0.0178 | 4.41E-67 | -3.89E-05 | 0.000134 | 0.77 | 0.041839 | 296.1867 |
| rs11249215 | A | G | 1 | 25297184 | 0.1403 | 0.016 | 2.14E-18 | -3.61E-05 | 0.000127 | 0.780001 | 0.009815 | 67.2345 |
| rs115059666 | A | G | 5 | 1.5E+08 | 0.5147 | 0.0744 | 4.47E-12 | 0.000149 | 0.000598 | 0.8 | 0.005997 | 40.92397 |
| rs11581607 | A | G | 1 | 67707690 | -0.3647 | 0.036 | 4.56E-24 | -0.00023 | 0.000252 | 0.36 | 0.01658 | 114.3606 |
| rs11767350 | G | A | 7 | 37385365 | -0.0998 | 0.0164 | 1.19E-09 | -6.11E-05 | 0.000127 | 0.630001 | 0.00487 | 33.19525 |
| rs11795343 | C | T | 9 | 32523737 | -0.1062 | 0.0164 | 1.02E-10 | 0.000126 | 0.000129 | 0.33 | 0.00542 | 36.96564 |
| rs118002009 | T | G | 11 | 64124980 | -0.102 | 0.0169 | 1.62E-09 | 0.000128 | 0.000127 | 0.31 | 0.005145 | 35.07972 |
| rs12133684 | A | G | 1 | 2.07E+08 | 0.1218 | 0.0206 | 3.26E-09 | 8.61E-05 | 0.000159 | 0.59 | 0.004754 | 32.40076 |
| rs12188300 | T | A | 5 | 1.59E+08 | 0.5005 | 0.0264 | 5.39E-80 | 0.000156 | 0.000216 | 0.47 | 0.042434 | 300.5882 |
| rs12211087 | A | T | 6 | 31269946 | 1.3769 | 0.0253 | 1.00E-200 | 0.000288 | 0.000219 | 0.19 | 0.312265 | 3079.823 |
| rs1295685 | G | A | 5 | 1.32E+08 | 0.1759 | 0.0206 | 1.16E-17 | -4.21E-05 | 0.000163 | 0.8 | 0.009166 | 62.74995 |
| rs131656 | A | G | 22 | 21917450 | 0.114 | 0.0202 | 1.69E-08 | 7.00E-05 | 0.00016 | 0.66 | 0.004028 | 27.43076 |
| rs142502677 | A | T | 18 | 51791307 | 0.1035 | 0.0185 | 2.22E-08 | 0.000294 | 0.000144 | 0.041 | 0.004251 | 28.95687 |
| rs1611236 | A | G | 6 | 29748690 | 0.123 | 0.0172 | 7.90E-13 | 0.000207 | 0.000135 | 0.12 | 0.006625 | 45.23954 |
| rs1648153 | A | G | 11 | 1.1E+08 | -0.1388 | 0.0164 | 2.93E-17 | 5.00E-05 | 0.000127 | 0.69 | 0.009405 | 64.40279 |
| rs2021511 | T | C | 16 | 11344903 | -0.1163 | 0.0181 | 1.20E-10 | 9.26E-05 | 0.00014 | 0.51 | 0.005427 | 37.01215 |
| rs2066819 | T | C | 12 | 56750204 | -0.3283 | 0.0348 | 4.28E-21 | 0.000433 | 0.000253 | 0.087 | 0.013297 | 91.41106 |
| rs2111485 | G | A | 2 | 1.63E+08 | 0.1616 | 0.0166 | 1.76E-22 | 6.25E-05 | 0.000129 | 0.630001 | 0.012452 | 85.52845 |
| rs2451258 | T | C | 6 | 1.6E+08 | -0.0985 | 0.0169 | 5.26E-09 | -1.65E-05 | 0.00013 | 0.9 | 0.004535 | 30.90416 |
| rs2638281 | G | A | 19 | 49210869 | 0.0976 | 0.0164 | 2.46E-09 | 9.69E-05 | 0.000129 | 0.450001 | 0.004561 | 31.07838 |
| rs2675662 | G | A | 10 | 75599127 | -0.1184 | 0.0167 | 1.49E-12 | -0.0001 | 0.000127 | 0.41 | 0.006894 | 47.08942 |
| rs2735009 | A | G | 6 | 29807853 | -0.2952 | 0.0204 | 1.63E-47 | -0.00024 | 0.000155 | 0.12 | 0.028584 | 199.5931 |
| rs28510484 | C | G | 15 | 31637569 | -0.1398 | 0.0231 | 1.37E-09 | -0.00011 | 0.000168 | 0.53 | 0.005516 | 37.61929 |
| rs28752856 | G | C | 6 | 31298421 | 1.1163 | 0.0225 | 1.00E-200 | 0.000266 | 0.000198 | 0.18 | 0.26017 | 2385.323 |
| rs28998802 | A | G | 17 | 26124908 | 0.2113 | 0.0222 | 1.89E-21 | 0.000295 | 0.000183 | 0.11 | 0.010794 | 74.01575 |
| rs34536443 | C | G | 19 | 10463118 | -0.6799 | 0.0504 | 2.02E-41 | -3.39E-05 | 0.000312 | 0.91 | 0.039823 | 281.3254 |
| rs35741374 | T | C | 2 | 61072567 | 0.1634 | 0.0163 | 1.08E-23 | 0.000118 | 0.000127 | 0.36 | 0.013091 | 89.97565 |
| rs3906814 | G | C | 3 | 17011474 | -0.1144 | 0.0161 | 1.30E-12 | 3.98E-08 | 0.000126 | 1 | 0.006543 | 44.67106 |
| rs39841 | A | G | 5 | 96120170 | -0.1624 | 0.0176 | 2.56E-20 | -0.00028 | 0.000141 | 0.05 | 0.010449 | 71.62241 |
| rs438650 | C | T | 1 | 8290082 | 0.1244 | 0.0195 | 1.76E-10 | 0.00013 | 0.000151 | 0.39 | 0.005463 | 37.2586 |
| rs4712520 | C | T | 6 | 20640871 | 0.1301 | 0.0212 | 8.27E-10 | -0.0002 | 0.000163 | 0.21 | 0.005065 | 34.52842 |
| rs4889526 | A | C | 16 | 31030344 | 0.1216 | 0.0165 | 1.62E-13 | 0.000141 | 0.00013 | 0.28 | 0.006924 | 47.29395 |
| rs4978343 | T | G | 9 | 1.11E+08 | -0.0988 | 0.0164 | 1.77E-09 | 1.92E-05 | 0.000129 | 0.88 | 0.00472 | 32.17021 |
| rs55868394 | A | C | 17 | 73851113 | 0.1389 | 0.0246 | 1.71E-08 | -0.00013 | 0.000191 | 0.51 | 0.004256 | 28.99083 |
| rs559406 | T | G | 18 | 12857002 | -0.0924 | 0.0161 | 1.02E-08 | -0.00014 | 0.000126 | 0.26 | 0.004232 | 28.82858 |
| rs582757 | T | C | 6 | 1.38E+08 | -0.1846 | 0.0176 | 1.15E-25 | -6.79E-05 | 0.000141 | 0.630001 | 0.013563 | 93.26523 |
| rs59960858 | A | C | 1 | 24519437 | -0.2086 | 0.0243 | 1.06E-17 | -0.00016 | 0.000184 | 0.38 | 0.010127 | 69.39274 |
| rs6894840 | G | T | 5 | 1.59E+08 | 0.1152 | 0.0166 | 3.42E-12 | 0.000142 | 0.00013 | 0.27 | 0.00627 | 42.7969 |
| rs7141014 | C | T | 14 | 98667928 | -0.1123 | 0.0203 | 3.07E-08 | 2.17E-05 | 0.000155 | 0.89 | 0.004173 | 28.42274 |
| rs73986523 | C | T | 17 | 40302271 | -0.2122 | 0.038 | 2.41E-08 | -0.00012 | 0.000263 | 0.64 | 0.005164 | 35.20789 |
| rs771576 | C | T | 3 | 1.02E+08 | 0.1029 | 0.0169 | 1.08E-09 | -7.74E-05 | 0.00013 | 0.55 | 0.005001 | 34.09019 |
| rs8016947 | G | T | 14 | 35832666 | 0.1476 | 0.0162 | 8.18E-20 | 9.46E-05 | 0.000127 | 0.46 | 0.010726 | 73.54188 |
| rs9258357 | C | T | 6 | 29751129 | 0.2839 | 0.0233 | 3.48E-34 | -3.48E-05 | 0.00017 | 0.84 | 0.021924 | 152.0478 |
| rs9264277 | C | T | 6 | 31224667 | 0.3705 | 0.018 | 1.91E-94 | 0.000109 | 0.000131 | 0.4 | 0.063719 | 461.6246 |
| rs9277939 | A | C | 6 | 33189873 | 0.228 | 0.0253 | 2.16E-19 | -0.00024 | 0.000215 | 0.25 | 0.009049 | 61.93974 |
| rs9468618 | T | C | 6 | 29750776 | -0.1856 | 0.03 | 6.49E-10 | -0.00026 | 0.00022 | 0.23 | 0.005616 | 38.30524 |
| rs9481169 | T | G | 6 | 1.12E+08 | 0.3716 | 0.0263 | 2.47E-45 | 0.000212 | 0.000225 | 0.35 | 0.021623 | 149.9073 |
| rs9513593 | A | G | 13 | 99950260 | -0.1197 | 0.0205 | 5.33E-09 | -0.00035 | 0.000168 | 0.036 | 0.004004 | 27.2706 |
| rs957970 | G | A | 17 | 40519890 | 0.0929 | 0.0168 | 3.05E-08 | -0.0002 | 0.000132 | 0.13 | 0.003925 | 26.72617 |
| rs9591325 | C | T | 13 | 50811220 | -0.19 | 0.0324 | 4.50E-09 | -0.00015 | 0.000251 | 0.55 | 0.004575 | 31.17749 |
| rs9695923 | T | C | 9 | 1.11E+08 | -0.0955 | 0.0165 | 7.08E-09 | 7.69E-05 | 0.000129 | 0.55 | 0.004389 | 29.9018 |

Supplementary Table 3. Genetic instrumental tools used in Mendelian randomization analysis on psoriasis and skin cancer, based on Stuart PE et al.

| SNP | Effect allele | Other allele | Chr | Pos | Exposure | | | Outcome | | | R^2^ | F |
| --- | --- | --- | --- | --- | --- | --- | --- | --- | --- | --- | --- | --- |
|  |  |  |  |  | beta | se | pval | beta | se | pval |  |  |
| rs10888503 | T | C | 1 | 1.53E+08 | 0.1866 | 0.0171 | 1.44E-27 | 0.000625 | 0.000249 | 0.012 | 0.015678 | 141.8012 |
| rs10893884 | C | T | 11 | 1.28E+08 | -0.106 | 0.016 | 3.99E-11 | 0.000363 | 0.000237 | 0.12 | 0.005614 | 50.26353 |
| rs11085744 | T | C | 19 | 10819967 | 0.1146 | 0.0166 | 5.21E-12 | 0.000257 | 0.000239 | 0.28 | 0.006476 | 58.02896 |
| rs1108618 | G | A | 10 | 81043743 | -0.1098 | 0.0165 | 3.25E-11 | -0.00023 | 0.000238 | 0.33 | 0.005912 | 52.94328 |
| rs11135059 | A | G | 5 | 1.59E+08 | -0.3078 | 0.0178 | 4.41E-67 | -0.00036 | 0.000251 | 0.16 | 0.041834 | 388.7114 |
| rs11249215 | A | G | 1 | 25297184 | 0.1403 | 0.016 | 2.14E-18 | -3.43E-05 | 0.000237 | 0.88 | 0.009814 | 88.24365 |
| rs11581607 | A | G | 1 | 67707690 | -0.3647 | 0.036 | 4.56E-24 | -6.92E-05 | 0.000472 | 0.88 | 0.016573 | 150.0335 |
| rs11767350 | G | A | 7 | 37385365 | -0.0998 | 0.0164 | 1.19E-09 | -0.00012 | 0.000238 | 0.630001 | 0.004871 | 43.58248 |
| rs11795343 | C | T | 9 | 32523737 | -0.1062 | 0.0164 | 1.02E-10 | 0.000658 | 0.000241 | 0.0064 | 0.00542 | 48.51383 |
| rs118002009 | T | G | 11 | 64124980 | -0.102 | 0.0169 | 1.62E-09 | 0.000695 | 0.000237 | 0.0034 | 0.005146 | 46.04872 |
| rs12133684 | A | G | 1 | 2.07E+08 | 0.1218 | 0.0206 | 3.26E-09 | -0.00015 | 0.000299 | 0.61 | 0.004751 | 42.49885 |
| rs12188300 | T | A | 5 | 1.59E+08 | 0.5005 | 0.0264 | 5.39E-80 | -0.00026 | 0.000405 | 0.53 | 0.042454 | 394.7255 |
| rs12211087 | A | T | 6 | 31289714 | 1.3769 | 0.0253 | 1.00E-200 | 0.000167 | 0.00041 | 0.68 | 0.312604 | 4048.78 |
| rs1295685 | G | A | 5 | 1.32E+08 | 0.1759 | 0.0206 | 1.16E-17 | -0.0004 | 0.000306 | 0.19 | 0.009172 | 82.41445 |
| rs131656 | A | G | 22 | 21917450 | 0.114 | 0.0202 | 1.69E-08 | -0.00037 | 0.000299 | 0.21 | 0.004021 | 35.94208 |
| rs1611236 | A | G | 6 | 29752278 | 0.123 | 0.0172 | 7.90E-13 | 0.000628 | 0.000252 | 0.013 | 0.006632 | 59.43647 |
| rs1648153 | A | G | 11 | 1.1E+08 | -0.1388 | 0.0164 | 2.93E-17 | 0.000257 | 0.000239 | 0.28 | 0.009395 | 84.43494 |
| rs2021511 | T | C | 16 | 11344903 | -0.1163 | 0.0181 | 1.20E-10 | 0.000231 | 0.000263 | 0.38 | 0.005426 | 48.56843 |
| rs2066819 | T | C | 12 | 56750204 | -0.3283 | 0.0348 | 4.28E-21 | 0.000374 | 0.000474 | 0.43 | 0.013339 | 120.3643 |
| rs2111485 | G | A | 2 | 1.63E+08 | 0.1616 | 0.0166 | 1.76E-22 | -0.00027 | 0.000241 | 0.27 | 0.012462 | 112.3526 |
| rs2451258 | T | C | 6 | 1.6E+08 | -0.0985 | 0.0169 | 5.26E-09 | -0.00025 | 0.000244 | 0.31 | 0.004538 | 40.58791 |
| rs2638281 | G | A | 19 | 49210869 | 0.0976 | 0.0164 | 2.46E-09 | -0.00025 | 0.000241 | 0.31 | 0.004562 | 40.79876 |
| rs2675662 | G | A | 10 | 75599127 | -0.1184 | 0.0167 | 1.49E-12 | -0.00068 | 0.000238 | 0.0044 | 0.006894 | 61.80201 |
| rs2735009 | A | G | 6 | 29807853 | -0.2952 | 0.0204 | 1.63E-47 | -0.00064 | 0.000291 | 0.028 | 0.028574 | 261.8814 |
| rs28510484 | C | G | 15 | 31637569 | -0.1398 | 0.0231 | 1.37E-09 | 2.60E-05 | 0.000314 | 0.93 | 0.005526 | 49.4745 |
| rs28752856 | G | C | 6 | 31298547 | 1.1163 | 0.0225 | 1.00E-200 | -0.00055 | 0.00042 | 0.19 | 0.341404 | 4615.17 |
| rs28998802 | A | G | 17 | 26124908 | 0.2113 | 0.0222 | 1.89E-21 | 0.000226 | 0.000344 | 0.51 | 0.010782 | 97.03924 |
| rs35741374 | T | C | 2 | 61072567 | 0.1634 | 0.0163 | 1.08E-23 | 8.14E-05 | 0.000238 | 0.73 | 0.013092 | 118.103 |
| rs3906814 | G | C | 3 | 17011474 | -0.1144 | 0.0161 | 1.30E-12 | 0.00023 | 0.000236 | 0.33 | 0.006543 | 58.63267 |
| rs39841 | A | G | 5 | 96120170 | -0.1624 | 0.0176 | 2.56E-20 | -0.00053 | 0.000264 | 0.044 | 0.010466 | 94.16586 |
| rs438650 | C | T | 1 | 8290082 | 0.1244 | 0.0195 | 1.76E-10 | 0.000128 | 0.000283 | 0.649999 | 0.005461 | 48.88971 |
| rs4712520 | C | T | 6 | 20640871 | 0.1301 | 0.0212 | 8.27E-10 | -0.00035 | 0.000305 | 0.24 | 0.005073 | 45.39917 |
| rs4889526 | A | C | 16 | 31030344 | 0.1216 | 0.0165 | 1.62E-13 | -0.00012 | 0.000244 | 0.61 | 0.006928 | 62.1117 |
| rs4978343 | T | G | 9 | 1.11E+08 | -0.0988 | 0.0164 | 1.77E-09 | -0.00023 | 0.000241 | 0.34 | 0.004721 | 42.23334 |
| rs55868394 | A | C | 17 | 73873394 | 0.1389 | 0.0246 | 1.71E-08 | 8.52E-06 | 0.000331 | 0.98 | 0.004895 | 43.79826 |
| rs559406 | T | G | 18 | 12857002 | -0.0924 | 0.0161 | 1.02E-08 | 0.000476 | 0.000237 | 0.045 | 0.004231 | 37.82963 |
| rs582757 | T | C | 6 | 1.38E+08 | -0.1846 | 0.0176 | 1.15E-25 | 0.000339 | 0.000264 | 0.2 | 0.01355 | 122.2953 |
| rs59960858 | A | C | 1 | 24519437 | -0.2086 | 0.0243 | 1.06E-17 | 0.000189 | 0.000345 | 0.58 | 0.010117 | 90.9967 |
| rs6063454 | T | G | 20 | 48590791 | -0.1551 | 0.0166 | 1.08E-20 | 0.000152 | 0.000241 | 0.53 | 0.011671 | 105.13 |
| rs6894840 | G | T | 5 | 1.59E+08 | 0.1152 | 0.0166 | 3.42E-12 | 0.000179 | 0.000243 | 0.46 | 0.006271 | 56.18634 |
| rs7141014 | C | T | 14 | 98667928 | -0.1123 | 0.0203 | 3.07E-08 | -0.00061 | 0.000291 | 0.036 | 0.004171 | 37.29218 |
| rs73986523 | C | T | 17 | 40302271 | -0.2122 | 0.038 | 2.41E-08 | -6.76E-05 | 0.000493 | 0.89 | 0.005163 | 46.2084 |
| rs771576 | C | T | 3 | 1.02E+08 | 0.1029 | 0.0169 | 1.08E-09 | -0.00027 | 0.000243 | 0.27 | 0.005001 | 44.74918 |
| rs8016947 | G | T | 14 | 35832666 | 0.1476 | 0.0162 | 8.18E-20 | 0.000335 | 0.000238 | 0.16 | 0.010726 | 96.53196 |
| rs9264277 | C | T | 6 | 31227901 | 0.3705 | 0.018 | 1.91E-94 | -0.00027 | 0.000245 | 0.28 | 0.063748 | 606.1924 |
| rs9277939 | A | C | 6 | 33189873 | 0.228 | 0.0253 | 2.16E-19 | 0.000291 | 0.000402 | 0.47 | 0.009048 | 81.29416 |
| rs9468618 | T | C | 6 | 29750776 | -0.1856 | 0.03 | 6.49E-10 | -1.84E-05 | 0.000412 | 0.96 | 0.00563 | 50.41126 |
| rs9481169 | T | G | 6 | 1.12E+08 | 0.3716 | 0.0263 | 2.47E-45 | -0.00011 | 0.000423 | 0.79 | 0.021579 | 196.3599 |
| rs9504361 | G | A | 6 | 577820 | -0.1068 | 0.0164 | 6.44E-11 | -0.00083 | 0.000243 | 0.00059 | 0.005638 | 50.48321 |
| rs9513593 | A | G | 13 | 99950260 | -0.1197 | 0.0205 | 5.33E-09 | -0.00072 | 0.000315 | 0.023 | 0.004007 | 35.81678 |
| rs957970 | G | A | 17 | 40519890 | 0.0929 | 0.0168 | 3.05E-08 | -0.0002 | 0.000247 | 0.42 | 0.003923 | 35.06639 |
| rs9591325 | C | T | 13 | 50811220 | -0.19 | 0.0324 | 4.50E-09 | 0.00035 | 0.000469 | 0.46 | 0.004576 | 40.92803 |

Supplementary Table 4. Genetic instrumental tools used in Mendelian randomization analysis on psoriasis and melanoma, based on Stuart PE et al.

| SNP | Effect allele | Other allele | Chr | Pos | Exposure | | | Outcome | | | R^2^ | F |
| --- | --- | --- | --- | --- | --- | --- | --- | --- | --- | --- | --- | --- |
|  |  |  |  |  | beta | se | pval | beta | se | pval |  |  |
| rs10888503 | T | C | 1 | 1.53E+08 | 0.1866 | 0.0171 | 1.44E-27 | -0.00016 | 0.000105 | 0.13 | 0.015678 | 283.6184 |
| rs10893884 | C | T | 11 | 1.28E+08 | -0.106 | 0.016 | 3.99E-11 | 2.73E-05 | 9.95E-05 | 0.780001 | 0.005614 | 100.5327 |
| rs11085744 | T | C | 19 | 10819967 | 0.1146 | 0.0166 | 5.21E-12 | -1.07E-05 | 0.0001 | 0.92 | 0.006476 | 116.0644 |
| rs1108618 | G | A | 10 | 81043743 | -0.1098 | 0.0165 | 3.25E-11 | 1.48E-05 | 0.0001 | 0.88 | 0.005912 | 105.8925 |
| rs11249215 | A | G | 1 | 25297184 | 0.1403 | 0.016 | 2.14E-18 | -8.70E-05 | 9.98E-05 | 0.38 | 0.009814 | 176.4972 |
| rs11767350 | G | A | 7 | 37385365 | -0.0998 | 0.0164 | 1.19E-09 | 0.000113 | 0.0001 | 0.26 | 0.004871 | 87.16985 |
| rs11795343 | C | T | 9 | 32523737 | -0.1062 | 0.0164 | 1.02E-10 | 0.000108 | 0.000102 | 0.29 | 0.00542 | 97.03311 |
| rs118002009 | T | G | 11 | 64124980 | -0.102 | 0.0169 | 1.62E-09 | 0.000125 | 9.98E-05 | 0.21 | 0.005146 | 92.10261 |
| rs1611236 | A | G | 6 | 29753454 | 0.123 | 0.0172 | 7.90E-13 | 5.32E-05 | 0.000103 | 0.61 | 0.006969 | 124.9743 |
| rs1648153 | A | G | 11 | 1.1E+08 | -0.1388 | 0.0164 | 2.93E-17 | -1.37E-05 | 0.0001 | 0.89 | 0.009395 | 168.8794 |
| rs2111485 | G | A | 2 | 1.63E+08 | 0.1616 | 0.0166 | 1.76E-22 | -0.00016 | 0.000101 | 0.11 | 0.012462 | 224.7178 |
| rs2451258 | T | C | 6 | 1.6E+08 | -0.0985 | 0.0169 | 5.26E-09 | -8.73E-06 | 0.000103 | 0.93 | 0.004538 | 81.18039 |
| rs2638281 | G | A | 19 | 49210869 | 0.0976 | 0.0164 | 2.46E-09 | -9.52E-05 | 0.000101 | 0.35 | 0.004562 | 81.60211 |
| rs2675662 | G | A | 10 | 75599127 | -0.1184 | 0.0167 | 1.49E-12 | 8.11E-05 | 0.0001 | 0.42 | 0.006894 | 123.611 |
| rs35741374 | T | C | 2 | 61072567 | 0.1634 | 0.0163 | 1.08E-23 | 0.000176 | 0.0001 | 0.079001 | 0.013092 | 236.2193 |
| rs3906814 | G | C | 3 | 17011474 | -0.1144 | 0.0161 | 1.30E-12 | 5.63E-05 | 9.94E-05 | 0.57 | 0.006543 | 117.2719 |
| rs4889526 | A | C | 16 | 31030344 | 0.1216 | 0.0165 | 1.62E-13 | -0.00025 | 0.000103 | 0.014 | 0.006928 | 124.2304 |
| rs4978343 | T | G | 9 | 1.11E+08 | -0.0988 | 0.0164 | 1.77E-09 | -4.46E-05 | 0.000101 | 0.66 | 0.004721 | 84.47143 |
| rs559406 | T | G | 18 | 12857002 | -0.0924 | 0.0161 | 1.02E-08 | 7.16E-05 | 9.97E-05 | 0.47 | 0.004231 | 75.66351 |
| rs6063454 | T | G | 20 | 48590791 | -0.1551 | 0.0166 | 1.08E-20 | -7.98E-05 | 0.000101 | 0.43 | 0.011671 | 210.2718 |
| rs6894840 | G | T | 5 | 1.59E+08 | 0.1152 | 0.0166 | 3.42E-12 | -6.71E-05 | 0.000102 | 0.51 | 0.006271 | 112.379 |
| rs771576 | C | T | 3 | 1.02E+08 | 0.1029 | 0.0169 | 1.08E-09 | 2.00E-05 | 0.000102 | 0.84 | 0.005001 | 89.50339 |
| rs8016947 | G | T | 14 | 35832666 | 0.1476 | 0.0162 | 8.18E-20 | -1.56E-06 | 0.0001 | 0.99 | 0.010726 | 193.0748 |
| rs9264277 | C | T | 6 | 31227901 | 0.3705 | 0.018 | 1.91E-94 | -9.18E-05 | 0.000103 | 0.37 | 0.063748 | 1212.453 |
| rs9504361 | G | A | 6 | 577820 | -0.1068 | 0.0164 | 6.44E-11 | -2.95E-05 | 0.000102 | 0.77 | 0.005638 | 100.9721 |
| rs957970 | G | A | 17 | 40519890 | 0.0929 | 0.0168 | 3.05E-08 | 5.53E-05 | 0.000104 | 0.6 | 0.003923 | 70.13672 |

Supplementary Table 5. Genetic instrumental tools used in Mendelian randomization analysis on psoriasis and multiple myeloma, based on Stuart PE et al.

| SNP | Effect allele | Other allele | Chr | Pos | Exposure | | | Outcome | | | R^2^ | F |
| --- | --- | --- | --- | --- | --- | --- | --- | --- | --- | --- | --- | --- |
|  |  |  |  |  | beta | se | pval | beta | se | pval |  |  |
| rs10888503 | T | C | 1 | 1.53E+08 | 0.1866 | 0.0171 | 1.44E-27 | -2.85E-05 | 9.83E-05 | 0.77 | 0.015673 | 109.8557 |
| rs10893884 | C | T | 11 | 1.28E+08 | -0.106 | 0.016 | 3.99E-11 | -2.88E-05 | 9.34E-05 | 0.760001 | 0.005614 | 38.9544 |
| rs11085744 | T | C | 19 | 10819967 | 0.1146 | 0.0166 | 5.21E-12 | -0.00017 | 9.41E-05 | 0.073 | 0.006476 | 44.96823 |
| rs1108618 | G | A | 10 | 81043743 | -0.1098 | 0.0165 | 3.25E-11 | 1.54E-05 | 9.40E-05 | 0.87 | 0.005911 | 41.02411 |
| rs11135059 | A | G | 5 | 1.59E+08 | -0.3078 | 0.0178 | 4.41E-67 | -6.34E-05 | 9.92E-05 | 0.52 | 0.041839 | 301.2607 |
| rs11249215 | A | G | 1 | 25297184 | 0.1403 | 0.016 | 2.14E-18 | -6.78E-06 | 9.37E-05 | 0.94 | 0.009815 | 68.38715 |
| rs11581607 | A | G | 1 | 67707690 | -0.3647 | 0.036 | 4.56E-24 | -1.68E-05 | 0.000186 | 0.93 | 0.016584 | 116.344 |
| rs11767350 | G | A | 7 | 37385365 | -0.0998 | 0.0164 | 1.19E-09 | 4.67E-05 | 9.41E-05 | 0.62 | 0.00487 | 33.76479 |
| rs11795343 | C | T | 9 | 32523737 | -0.1062 | 0.0164 | 1.02E-10 | -5.04E-06 | 9.53E-05 | 0.96 | 0.00542 | 37.59845 |
| rs118002009 | T | G | 11 | 64124980 | -0.102 | 0.0169 | 1.62E-09 | -0.00018 | 9.36E-05 | 0.058 | 0.005145 | 35.67995 |
| rs12133684 | A | G | 1 | 2.07E+08 | 0.1218 | 0.0206 | 3.26E-09 | -0.0001 | 0.000118 | 0.39 | 0.004753 | 32.95235 |
| rs12188300 | T | A | 5 | 1.59E+08 | 0.5005 | 0.0264 | 5.39E-80 | -3.97E-05 | 0.00016 | 0.8 | 0.042427 | 305.6828 |
| rs12211087 | A | T | 6 | 31269946 | 1.3769 | 0.0253 | 1.00E-200 | 1.18E-06 | 0.000162 | 0.99 | 0.312191 | 3131.532 |
| rs1295685 | G | A | 5 | 1.32E+08 | 0.1759 | 0.0206 | 1.16E-17 | 0.000114 | 0.000121 | 0.34 | 0.009165 | 63.82009 |
| rs131656 | A | G | 22 | 21917450 | 0.114 | 0.0202 | 1.69E-08 | -0.00013 | 0.000118 | 0.27 | 0.004027 | 27.89809 |
| rs142502677 | A | T | 18 | 51791307 | 0.1035 | 0.0185 | 2.22E-08 | -0.00017 | 0.000106 | 0.1 | 0.00425 | 29.44717 |
| rs1611236 | A | G | 6 | 29748690 | 0.123 | 0.0172 | 7.90E-13 | 0.000109 | 9.96E-05 | 0.27 | 0.006625 | 46.01335 |
| rs1648153 | A | G | 11 | 1.1E+08 | -0.1388 | 0.0164 | 2.93E-17 | -0.00011 | 9.41E-05 | 0.26 | 0.009405 | 65.50519 |
| rs2021511 | T | C | 16 | 11344903 | -0.1163 | 0.0181 | 1.20E-10 | 1.63E-05 | 0.000104 | 0.88 | 0.005427 | 37.64546 |
| rs2066819 | T | C | 12 | 56750204 | -0.3283 | 0.0348 | 4.28E-21 | 0.000295 | 0.000187 | 0.12 | 0.013295 | 92.96349 |
| rs2111485 | G | A | 2 | 1.63E+08 | 0.1616 | 0.0166 | 1.76E-22 | -5.86E-05 | 9.52E-05 | 0.54 | 0.012453 | 86.99725 |
| rs2451258 | T | C | 6 | 1.6E+08 | -0.0985 | 0.0169 | 5.26E-09 | -9.78E-06 | 9.64E-05 | 0.92 | 0.004535 | 31.43379 |
| rs2638281 | G | A | 19 | 49210869 | 0.0976 | 0.0164 | 2.46E-09 | 1.59E-05 | 9.51E-05 | 0.87 | 0.004561 | 31.61188 |
| rs2675662 | G | A | 10 | 75599127 | -0.1184 | 0.0167 | 1.49E-12 | -8.24E-05 | 9.39E-05 | 0.38 | 0.006894 | 47.89676 |
| rs2735009 | A | G | 6 | 29807853 | -0.2952 | 0.0204 | 1.63E-47 | 3.22E-05 | 0.000115 | 0.780001 | 0.028589 | 203.052 |
| rs28510484 | C | G | 15 | 31637569 | -0.1398 | 0.0231 | 1.37E-09 | 4.43E-05 | 0.000124 | 0.719999 | 0.005516 | 38.26795 |
| rs28752856 | G | C | 6 | 31298421 | 1.1163 | 0.0225 | 1.00E-200 | 6.48E-05 | 0.000146 | 0.66 | 0.260135 | 2425.782 |
| rs28998802 | A | G | 17 | 26124908 | 0.2113 | 0.0222 | 1.89E-21 | 4.79E-05 | 0.000136 | 0.719999 | 0.010792 | 75.27054 |
| rs34536443 | C | G | 19 | 10463118 | -0.6799 | 0.0504 | 2.02E-41 | -0.00014 | 0.000231 | 0.55 | 0.03982 | 286.1228 |
| rs35741374 | T | C | 2 | 61072567 | 0.1634 | 0.0163 | 1.08E-23 | 5.19E-05 | 9.41E-05 | 0.58 | 0.013091 | 91.51901 |
| rs3906814 | G | C | 3 | 17011474 | -0.1144 | 0.0161 | 1.30E-12 | -1.19E-05 | 9.32E-05 | 0.9 | 0.006543 | 45.43684 |
| rs39841 | A | G | 5 | 96120170 | -0.1624 | 0.0176 | 2.56E-20 | -0.00014 | 0.000104 | 0.18 | 0.010448 | 72.84614 |
| rs438650 | C | T | 1 | 8290082 | 0.1244 | 0.0195 | 1.76E-10 | -0.00012 | 0.000112 | 0.29 | 0.005462 | 37.89204 |
| rs4712520 | C | T | 6 | 20640871 | 0.1301 | 0.0212 | 8.27E-10 | 7.19E-05 | 0.00012 | 0.55 | 0.005064 | 35.11389 |
| rs4889526 | A | C | 16 | 31030344 | 0.1216 | 0.0165 | 1.62E-13 | 3.67E-05 | 9.62E-05 | 0.7 | 0.006924 | 48.10328 |
| rs4978343 | T | G | 9 | 1.11E+08 | -0.0988 | 0.0164 | 1.77E-09 | 2.11E-06 | 9.51E-05 | 0.98 | 0.00472 | 32.72164 |
| rs55868394 | A | C | 17 | 73851113 | 0.1389 | 0.0246 | 1.71E-08 | 0.000304 | 0.000141 | 0.031 | 0.004257 | 29.49683 |
| rs559406 | T | G | 18 | 12857002 | -0.0924 | 0.0161 | 1.02E-08 | 0.00011 | 9.35E-05 | 0.24 | 0.004232 | 29.32208 |
| rs582757 | T | C | 6 | 1.38E+08 | -0.1846 | 0.0176 | 1.15E-25 | 3.55E-05 | 0.000104 | 0.73 | 0.013563 | 94.85966 |
| rs59960858 | A | C | 1 | 24519437 | -0.2086 | 0.0243 | 1.06E-17 | 3.84E-05 | 0.000136 | 0.780001 | 0.010128 | 70.59306 |
| rs6894840 | G | T | 5 | 1.59E+08 | 0.1152 | 0.0166 | 3.42E-12 | -1.55E-05 | 9.60E-05 | 0.87 | 0.00627 | 43.53216 |
| rs7141014 | C | T | 14 | 98667928 | -0.1123 | 0.0203 | 3.07E-08 | 3.82E-05 | 0.000115 | 0.74 | 0.004173 | 28.91008 |
| rs73986523 | C | T | 17 | 40302271 | -0.2122 | 0.038 | 2.41E-08 | -8.14E-05 | 0.000195 | 0.68 | 0.005164 | 35.81254 |
| rs771576 | C | T | 3 | 1.02E+08 | 0.1029 | 0.0169 | 1.08E-09 | 9.34E-05 | 9.59E-05 | 0.33 | 0.005001 | 34.6734 |
| rs8016947 | G | T | 14 | 35832666 | 0.1476 | 0.0162 | 8.18E-20 | -7.96E-05 | 9.38E-05 | 0.4 | 0.010726 | 74.80425 |
| rs9258357 | C | T | 6 | 29751129 | 0.2839 | 0.0233 | 3.48E-34 | -0.0001 | 0.000126 | 0.42 | 0.021925 | 154.6606 |
| rs9264277 | C | T | 6 | 31224667 | 0.3705 | 0.018 | 1.91E-94 | -4.31E-05 | 9.68E-05 | 0.66 | 0.063722 | 469.556 |
| rs9277939 | A | C | 6 | 33189873 | 0.228 | 0.0253 | 2.16E-19 | 3.36E-05 | 0.000159 | 0.83 | 0.009051 | 63.01688 |
| rs9468618 | T | C | 6 | 29750776 | -0.1856 | 0.03 | 6.49E-10 | -0.00026 | 0.000163 | 0.11 | 0.005616 | 38.96228 |
| rs9481169 | T | G | 6 | 1.12E+08 | 0.3716 | 0.0263 | 2.47E-45 | 0.000243 | 0.000167 | 0.15 | 0.021623 | 152.4821 |
| rs9513593 | A | G | 13 | 99950260 | -0.1197 | 0.0205 | 5.33E-09 | -0.00014 | 0.000125 | 0.27 | 0.004004 | 27.73438 |
| rs957970 | G | A | 17 | 40519890 | 0.0929 | 0.0168 | 3.05E-08 | -0.00011 | 9.76E-05 | 0.28 | 0.003925 | 27.18512 |
| rs9591325 | C | T | 13 | 50811220 | -0.19 | 0.0324 | 4.50E-09 | -0.00016 | 0.000185 | 0.38 | 0.004575 | 31.71238 |
| rs9695923 | T | C | 9 | 1.11E+08 | -0.0955 | 0.0165 | 7.08E-09 | -3.18E-05 | 9.52E-05 | 0.74 | 0.004389 | 30.4138 |

Supplementary Table 6. Genetic instrumental tools used in Mendelian randomization analysis on psoriasis and breast cancer, based on Stuart PE et al.

| SNP | Effect allele | Other allele | Chr | Pos | Exposure | | | Outcome | | | R^2^ | F |
| --- | --- | --- | --- | --- | --- | --- | --- | --- | --- | --- | --- | --- |
|  |  |  |  |  | beta | se | pval | beta | se | pval |  |  |
| rs10888503 | T | C | 1 | 1.53E+08 | 0.1866 | 0.0171 | 1.44E-27 | 0.0033 | 0.0068 | 0.6298 | 0.015984 | 67.92738 |
| rs10893884 | C | T | 11 | 1.28E+08 | -0.106 | 0.016 | 3.99E-11 | -0.0088 | 0.0064 | 0.169 | 0.005613 | 24.10604 |
| rs11085744 | T | C | 19 | 10819967 | 0.1146 | 0.0166 | 5.21E-12 | -0.0137 | 0.0066 | 0.03682 | 0.006461 | 27.72356 |
| rs1108618 | G | A | 10 | 81043743 | -0.1098 | 0.0165 | 3.25E-11 | -0.0069 | 0.007 | 0.3198 | 0.005836 | 25.05821 |
| rs11135059 | A | G | 5 | 1.59E+08 | -0.3078 | 0.0178 | 4.41E-67 | 0.0268 | 0.0066 | 4.82E-05 | 0.041797 | 172.9698 |
| rs11249215 | A | G | 1 | 25297184 | 0.1403 | 0.016 | 2.14E-18 | 0.0182 | 0.0064 | 0.004653 | 0.009797 | 41.89716 |
| rs115059666 | A | G | 5 | 1.5E+08 | 0.5147 | 0.0744 | 4.47E-12 | -0.0049 | 0.0363 | 0.892 | 0.005868 | 25.19261 |
| rs11581607 | A | G | 1 | 67707690 | -0.3647 | 0.036 | 4.56E-24 | -0.0071 | 0.0126 | 0.5699 | 0.016005 | 68.01526 |
| rs11767350 | G | A | 7 | 37385365 | -0.0998 | 0.0164 | 1.19E-09 | -8.00E-04 | 0.0068 | 0.9064 | 0.004809 | 20.6686 |
| rs11795343 | C | T | 9 | 32523737 | -0.1062 | 0.0164 | 1.02E-10 | 0.0217 | 0.0063 | 0.000589 | 0.005436 | 23.34975 |
| rs118002009 | T | G | 11 | 64122279 | -0.102 | 0.0169 | 1.62E-09 | -5.00E-04 | 0.0063 | 0.9417 | 0.005117 | 21.98643 |
| rs12133684 | A | G | 1 | 2.07E+08 | 0.1218 | 0.0206 | 3.26E-09 | -0.0031 | 0.0082 | 0.7085 | 0.00485 | 20.84264 |
| rs12188300 | T | A | 5 | 1.59E+08 | 0.5005 | 0.0264 | 5.39E-80 | 0.0056 | 0.0121 | 0.643101 | 0.038006 | 157.9034 |
| rs12211087 | A | T | 6 | 31269946 | 1.3769 | 0.0253 | 1.00E-200 | 0.0213 | 0.0109 | 0.051591 | 0.305556 | 916.4158 |
| rs1295685 | G | A | 5 | 1.32E+08 | 0.1759 | 0.0206 | 1.16E-17 | 0.0096 | 0.0077 | 0.2129 | 0.010063 | 43.02383 |
| rs131656 | A | G | 22 | 21917450 | 0.114 | 0.0202 | 1.69E-08 | 0.0098 | 0.0077 | 0.2036 | 0.004235 | 18.21062 |
| rs1611236 | A | G | 6 | 29752932 | 0.123 | 0.0172 | 7.90E-13 | -0.0146 | 0.0068 | 0.03217 | 0.006618 | 28.39111 |
| rs1648153 | A | G | 11 | 1.1E+08 | -0.1388 | 0.0164 | 2.93E-17 | 0.003 | 0.0069 | 0.667799 | 0.009353 | 40.01614 |
| rs2021511 | T | C | 16 | 11344903 | -0.1163 | 0.0181 | 1.20E-10 | -0.0107 | 0.0072 | 0.1392 | 0.005407 | 23.22438 |
| rs2111485 | G | A | 2 | 1.63E+08 | 0.1616 | 0.0166 | 1.76E-22 | -0.018 | 0.0065 | 0.005437 | 0.012451 | 53.10592 |
| rs2301368 | A | G | 17 | 26130118 | 0.1073 | 0.0168 | 1.61E-10 | 0.0045 | 0.0064 | 0.4828 | 0.005552 | 23.84626 |
| rs2451258 | T | C | 6 | 1.6E+08 | -0.0985 | 0.0169 | 5.26E-09 | -0.012 | 0.0068 | 0.07651 | 0.004329 | 18.61342 |
| rs2638281 | G | A | 19 | 49210869 | 0.0976 | 0.0164 | 2.46E-09 | 0.01 | 0.0064 | 0.1186 | 0.004714 | 20.26251 |
| rs2675662 | G | A | 10 | 75599127 | -0.1184 | 0.0167 | 1.49E-12 | -0.0075 | 0.0063 | 0.234 | 0.006905 | 29.61528 |
| rs2735009 | A | G | 6 | 29807853 | -0.2952 | 0.0204 | 1.63E-47 | -8.00E-04 | 0.0074 | 0.9129 | 0.030362 | 127.1479 |
| rs28510484 | C | G | 15 | 31637569 | -0.1398 | 0.0231 | 1.37E-09 | -0.001 | 0.0094 | 0.9183 | 0.005341 | 22.9424 |
| rs28752856 | G | C | 6 | 31298421 | 1.1163 | 0.0225 | 1.00E-200 | 0.0064 | 0.0097 | 0.5072 | 0.266395 | 844.0188 |
| rs28998802 | A | G | 17 | 26124908 | 0.2113 | 0.0222 | 1.89E-21 | -0.0058 | 0.0093 | 0.5317 | 0.010388 | 44.39914 |
| rs34536443 | C | G | 19 | 10463118 | -0.6799 | 0.0504 | 2.02E-41 | -0.007 | 0.0175 | 0.6899 | 0.035076 | 146.1745 |
| rs35741374 | T | C | 2 | 61072567 | 0.1634 | 0.0163 | 1.08E-23 | -7.00E-04 | 0.0062 | 0.912 | 0.013161 | 56.09069 |
| rs3906814 | G | C | 3 | 17011474 | -0.1144 | 0.0161 | 1.30E-12 | 0.0064 | 0.0062 | 0.3011 | 0.006528 | 28.00792 |
| rs39841 | A | G | 5 | 96120170 | -0.1624 | 0.0176 | 2.56E-20 | 0.0037 | 0.007 | 0.597601 | 0.010673 | 45.60356 |
| rs438650 | C | T | 1 | 8290082 | 0.1244 | 0.0195 | 1.76E-10 | -0.0236 | 0.0076 | 0.001855 | 0.005414 | 23.25556 |
| rs4712520 | C | T | 6 | 20640871 | 0.1301 | 0.0212 | 8.27E-10 | -0.0327 | 0.0081 | 5.35E-05 | 0.004999 | 21.48063 |
| rs4889526 | A | C | 16 | 31030344 | 0.1216 | 0.0165 | 1.62E-13 | -0.0146 | 0.0065 | 0.02548 | 0.006919 | 29.67636 |
| rs4978343 | T | G | 9 | 1.11E+08 | -0.0988 | 0.0164 | 1.77E-09 | 0.0548 | 0.0063 | 2.23E-18 | 0.004713 | 20.25998 |
| rs55868394 | A | C | 17 | 73851113 | 0.1389 | 0.0246 | 1.71E-08 | 0.0114 | 0.0105 | 0.2739 | 0.004048 | 17.41307 |
| rs559406 | T | G | 18 | 12857002 | -0.0924 | 0.0161 | 1.02E-08 | 0.0106 | 0.0062 | 0.08843 | 0.004221 | 18.15394 |
| rs582757 | T | C | 6 | 1.38E+08 | -0.1846 | 0.0176 | 1.15E-25 | -0.0077 | 0.0073 | 0.294 | 0.013652 | 58.15705 |
| rs59960858 | A | C | 1 | 24519437 | -0.2086 | 0.0243 | 1.06E-17 | -0.0189 | 0.0092 | 0.03943 | 0.010472 | 44.75253 |
| rs6063454 | T | G | 20 | 48590791 | -0.1551 | 0.0166 | 1.08E-20 | 0.0036 | 0.0065 | 0.5775 | 0.011613 | 49.57098 |
| rs6894840 | G | T | 5 | 1.59E+08 | 0.1152 | 0.0166 | 3.42E-12 | -0.0214 | 0.0065 | 0.000935 | 0.00628 | 26.9501 |
| rs7141014 | C | T | 14 | 98667928 | -0.1123 | 0.0203 | 3.07E-08 | 0.008 | 0.008 | 0.3146 | 0.004066 | 17.48794 |
| rs73986523 | C | T | 17 | 40270062 | -0.2122 | 0.038 | 2.41E-08 | -0.0221 | 0.0135 | 0.1023 | 0.005119 | 21.99421 |
| rs771576 | C | T | 3 | 1.02E+08 | 0.1029 | 0.0169 | 1.08E-09 | 0.0023 | 0.0065 | 0.7229 | 0.005059 | 21.74002 |
| rs8016947 | G | T | 14 | 35832666 | 0.1476 | 0.0162 | 8.18E-20 | -0.0085 | 0.0063 | 0.1795 | 0.010724 | 45.81726 |
| rs9264277 | C | T | 6 | 31228593 | 0.3705 | 0.018 | 1.91E-94 | -0.0096 | 0.0066 | 0.146 | 0.061097 | 247.7458 |
| rs9277939 | A | C | 6 | 33189873 | 0.228 | 0.0253 | 2.16E-19 | -0.0291 | 0.0106 | 0.005921 | 0.00977 | 41.78421 |
| rs9481169 | T | G | 6 | 1.12E+08 | 0.3716 | 0.0263 | 2.47E-45 | -0.0158 | 0.0113 | 0.1625 | 0.021685 | 91.62438 |
| rs9504361 | G | A | 6 | 577820 | -0.1068 | 0.0164 | 6.44E-11 | -0.0124 | 0.0063 | 0.05168 | 0.005674 | 24.36399 |
| rs9513593 | A | G | 13 | 99950260 | -0.1197 | 0.0205 | 5.33E-09 | 1.00E-04 | 0.0081 | 0.9925 | 0.004197 | 18.04804 |
| rs957970 | G | A | 17 | 40519890 | 0.0929 | 0.0168 | 3.05E-08 | -0.001 | 0.0064 | 0.8795 | 0.003949 | 16.98671 |
| rs9591325 | C | T | 13 | 50811220 | -0.19 | 0.0324 | 4.50E-09 | -0.0045 | 0.0121 | 0.709601 | 0.004855 | 20.86602 |

Supplementary Table 7. Genetic instrumental tools used in Mendelian randomization analysis on psoriasis and lung cancer, based on Stuart PE et al.

| SNP | Effect allele | Other allele | Chr | Pos | Exposure | | | Outcome | | | R^2^ | F |
| --- | --- | --- | --- | --- | --- | --- | --- | --- | --- | --- | --- | --- |
|  |  |  |  |  | beta | se | pval | beta | se | pval |  |  |
| rs10888503 | T | C | 1 | 1.53E+08 | 0.1866 | 0.0171 | 1.44E-27 | -0.00022 | 0.000205 | 0.28 | 0.015674 | 108.463 |
| rs10893884 | C | T | 11 | 1.28E+08 | -0.106 | 0.016 | 3.99E-11 | 0.000227 | 0.000195 | 0.24 | 0.005614 | 38.45826 |
| rs11085744 | T | C | 19 | 10819967 | 0.1146 | 0.0166 | 5.21E-12 | 0.000398 | 0.000197 | 0.043 | 0.006475 | 44.39293 |
| rs1108618 | G | A | 10 | 81043743 | -0.1098 | 0.0165 | 3.25E-11 | -2.65E-05 | 0.000196 | 0.89 | 0.005911 | 40.50188 |
| rs11135059 | A | G | 5 | 1.59E+08 | -0.3078 | 0.0178 | 4.41E-67 | -0.00052 | 0.000207 | 0.012 | 0.041832 | 297.3788 |
| rs11249215 | A | G | 1 | 25297184 | 0.1403 | 0.016 | 2.14E-18 | 0.00021 | 0.000196 | 0.28 | 0.009815 | 67.51746 |
| rs115059666 | A | G | 5 | 1.5E+08 | 0.5147 | 0.0744 | 4.47E-12 | 0.000664 | 0.000924 | 0.47 | 0.006001 | 41.12063 |
| rs11581607 | A | G | 1 | 67707690 | -0.3647 | 0.036 | 4.56E-24 | -0.00024 | 0.000389 | 0.54 | 0.01658 | 114.8387 |
| rs11767350 | G | A | 7 | 37385365 | -0.0998 | 0.0164 | 1.19E-09 | 4.72E-05 | 0.000197 | 0.81 | 0.00487 | 33.33503 |
| rs11795343 | C | T | 9 | 32523737 | -0.1062 | 0.0164 | 1.02E-10 | 6.67E-06 | 0.000199 | 0.97 | 0.00542 | 37.11967 |
| rs118002009 | T | G | 11 | 64124980 | -0.102 | 0.0169 | 1.62E-09 | -9.29E-05 | 0.000196 | 0.630001 | 0.005145 | 35.22602 |
| rs12133684 | A | G | 1 | 2.07E+08 | 0.1218 | 0.0206 | 3.26E-09 | 0.000207 | 0.000246 | 0.4 | 0.004754 | 32.53876 |
| rs12188300 | T | A | 5 | 1.59E+08 | 0.5005 | 0.0264 | 5.39E-80 | -0.00052 | 0.000334 | 0.12 | 0.042412 | 301.6827 |
| rs12211087 | A | T | 6 | 31269946 | 1.3769 | 0.0253 | 1.00E-200 | 0.001077 | 0.000339 | 0.0015 | 0.312473 | 3095.739 |
| rs1295685 | G | A | 5 | 1.32E+08 | 0.1759 | 0.0206 | 1.16E-17 | 0.000177 | 0.000252 | 0.48 | 0.009165 | 63.00332 |
| rs131656 | A | G | 22 | 21917450 | 0.114 | 0.0202 | 1.69E-08 | 0.000172 | 0.000247 | 0.49 | 0.004028 | 27.54726 |
| rs142502677 | A | T | 18 | 51791307 | 0.1035 | 0.0185 | 2.22E-08 | 0.00014 | 0.000222 | 0.53 | 0.004251 | 29.07647 |
| rs1611236 | A | G | 6 | 29748690 | 0.123 | 0.0172 | 7.90E-13 | 0.000432 | 0.000208 | 0.038 | 0.006626 | 45.43322 |
| rs1648153 | A | G | 11 | 1.1E+08 | -0.1388 | 0.0164 | 2.93E-17 | 5.88E-05 | 0.000197 | 0.760001 | 0.009405 | 64.67304 |
| rs2021511 | T | C | 16 | 11344903 | -0.1163 | 0.0181 | 1.20E-10 | -0.0004 | 0.000217 | 0.069 | 0.005426 | 37.15905 |
| rs2066819 | T | C | 12 | 56750204 | -0.3283 | 0.0348 | 4.28E-21 | 0.000332 | 0.000391 | 0.4 | 0.013296 | 91.78798 |
| rs2111485 | G | A | 2 | 1.63E+08 | 0.1616 | 0.0166 | 1.76E-22 | -4.43E-06 | 0.000199 | 0.98 | 0.012452 | 85.88826 |
| rs2451258 | T | C | 6 | 1.6E+08 | -0.0985 | 0.0169 | 5.26E-09 | 0.000142 | 0.000201 | 0.48 | 0.004535 | 31.03268 |
| rs2638281 | G | A | 19 | 49210869 | 0.0976 | 0.0164 | 2.46E-09 | -6.11E-05 | 0.000199 | 0.760001 | 0.004561 | 31.20951 |
| rs2675662 | G | A | 10 | 75599127 | -0.1184 | 0.0167 | 1.49E-12 | 0.000121 | 0.000196 | 0.54 | 0.006895 | 47.28835 |
| rs2735009 | A | G | 6 | 29807853 | -0.2952 | 0.0204 | 1.63E-47 | -0.00042 | 0.00024 | 0.083 | 0.028581 | 200.4045 |
| rs28510484 | C | G | 15 | 31637569 | -0.1398 | 0.0231 | 1.37E-09 | 8.65E-05 | 0.000259 | 0.74 | 0.005516 | 37.78207 |
| rs28752856 | G | C | 6 | 31298421 | 1.1163 | 0.0225 | 1.00E-200 | -0.00058 | 0.000306 | 0.058 | 0.260004 | 2393.27 |
| rs28998802 | A | G | 17 | 26124908 | 0.2113 | 0.0222 | 1.89E-21 | -0.00018 | 0.000283 | 0.52 | 0.01079 | 74.30078 |
| rs34536443 | C | G | 19 | 10463118 | -0.6799 | 0.0504 | 2.02E-41 | 0.001252 | 0.000482 | 0.0094 | 0.039867 | 282.8287 |
| rs35741374 | T | C | 2 | 61072567 | 0.1634 | 0.0163 | 1.08E-23 | -0.00016 | 0.000197 | 0.41 | 0.013092 | 90.35658 |
| rs3906814 | G | C | 3 | 17011474 | -0.1144 | 0.0161 | 1.30E-12 | 0.000721 | 0.000195 | 0.00021 | 0.006543 | 44.85803 |
| rs39841 | A | G | 5 | 96120170 | -0.1624 | 0.0176 | 2.56E-20 | -0.00042 | 0.000218 | 0.056 | 0.010449 | 71.92721 |
| rs438650 | C | T | 1 | 8290082 | 0.1244 | 0.0195 | 1.76E-10 | 0.000481 | 0.000233 | 0.039 | 0.005464 | 37.42218 |
| rs4712520 | C | T | 6 | 20640871 | 0.1301 | 0.0212 | 8.27E-10 | -0.00036 | 0.000251 | 0.15 | 0.005065 | 34.67725 |
| rs4889526 | A | C | 16 | 31030344 | 0.1216 | 0.0165 | 1.62E-13 | -7.12E-05 | 0.000201 | 0.719999 | 0.006924 | 47.48983 |
| rs4978343 | T | G | 9 | 1.11E+08 | -0.0988 | 0.0164 | 1.77E-09 | 0.000336 | 0.000199 | 0.091 | 0.004721 | 32.30699 |
| rs55868394 | A | C | 17 | 73851113 | 0.1389 | 0.0246 | 1.71E-08 | 0.000219 | 0.000295 | 0.46 | 0.004257 | 29.11997 |
| rs559406 | T | G | 18 | 12857002 | -0.0924 | 0.0161 | 1.02E-08 | -0.00059 | 0.000195 | 0.0027 | 0.004232 | 28.95073 |
| rs582757 | T | C | 6 | 1.38E+08 | -0.1846 | 0.0176 | 1.15E-25 | -1.09E-05 | 0.000218 | 0.96 | 0.013563 | 93.65345 |
| rs59960858 | A | C | 1 | 24519437 | -0.2086 | 0.0243 | 1.06E-17 | -0.00026 | 0.000285 | 0.36 | 0.010126 | 69.67853 |
| rs6894840 | G | T | 5 | 1.59E+08 | 0.1152 | 0.0166 | 3.42E-12 | 0.000301 | 0.000201 | 0.13 | 0.00627 | 42.97475 |
| rs7141014 | C | T | 14 | 98667928 | -0.1123 | 0.0203 | 3.07E-08 | 0.000469 | 0.00024 | 0.051 | 0.004174 | 28.54933 |
| rs73986523 | C | T | 17 | 40302271 | -0.2122 | 0.038 | 2.41E-08 | 0.000104 | 0.000407 | 0.8 | 0.005165 | 35.36266 |
| rs771576 | C | T | 3 | 1.02E+08 | 0.1029 | 0.0169 | 1.08E-09 | 0.000162 | 0.0002 | 0.42 | 0.005 | 34.231 |
| rs8016947 | G | T | 14 | 35832666 | 0.1476 | 0.0162 | 8.18E-20 | 0.000226 | 0.000196 | 0.25 | 0.010726 | 73.84911 |
| rs9258357 | C | T | 6 | 29751129 | 0.2839 | 0.0233 | 3.48E-34 | -0.00043 | 0.000263 | 0.11 | 0.02193 | 152.7283 |
| rs9264277 | C | T | 6 | 31224667 | 0.3705 | 0.018 | 1.91E-94 | -0.00029 | 0.000202 | 0.15 | 0.063727 | 463.6182 |
| rs9277939 | A | C | 6 | 33189873 | 0.228 | 0.0253 | 2.16E-19 | -0.00026 | 0.000332 | 0.43 | 0.009049 | 62.19841 |
| rs9468618 | T | C | 6 | 29750776 | -0.1856 | 0.03 | 6.49E-10 | -0.00046 | 0.000341 | 0.18 | 0.005615 | 38.4597 |
| rs9481169 | T | G | 6 | 1.12E+08 | 0.3716 | 0.0263 | 2.47E-45 | -0.00049 | 0.000348 | 0.16 | 0.02161 | 150.445 |
| rs9513593 | A | G | 13 | 99950260 | -0.1197 | 0.0205 | 5.33E-09 | -6.67E-05 | 0.00026 | 0.8 | 0.004004 | 27.37964 |
| rs957970 | G | A | 17 | 40519890 | 0.0929 | 0.0168 | 3.05E-08 | 0.000337 | 0.000204 | 0.099001 | 0.003925 | 26.8426 |
| rs9591325 | C | T | 13 | 50811220 | -0.19 | 0.0324 | 4.50E-09 | -0.00053 | 0.000387 | 0.17 | 0.004574 | 31.29755 |
| rs9695923 | T | C | 9 | 1.11E+08 | -0.0955 | 0.0165 | 7.08E-09 | 0.000223 | 0.000199 | 0.26 | 0.004389 | 30.02806 |

Supplementary Table 8. Genetic instrumental tools used in Mendelian randomization analysis on psoriasis and esophageal cancer, based on Stuart PE et al.

| SNP | Effect allele | Other allele | Chr | Pos | Exposure | | | Outcome | | | R^2^ | F |
| --- | --- | --- | --- | --- | --- | --- | --- | --- | --- | --- | --- | --- |
|  |  |  |  |  | beta | se | pval | beta | se | pval |  |  |
| rs10888503 | T | C | 1 | 1.53E+08 | 0.1866 | 0.0171 | 1.44E-27 | 0.00018 | 0.000109 | 0.098001 | 0.015672 | 109.889 |
| rs10893884 | C | T | 11 | 1.28E+08 | -0.106 | 0.016 | 3.99E-11 | -1.08E-06 | 0.000103 | 0.99 | 0.005614 | 38.9689 |
| rs11085744 | T | C | 19 | 10819967 | 0.1146 | 0.0166 | 5.21E-12 | -8.38E-05 | 0.000104 | 0.42 | 0.006476 | 44.98457 |
| rs1108618 | G | A | 10 | 81043743 | -0.1098 | 0.0165 | 3.25E-11 | 0.000218 | 0.000104 | 0.037 | 0.005911 | 41.04066 |
| rs11135059 | A | G | 5 | 1.59E+08 | -0.3078 | 0.0178 | 4.41E-67 | 3.72E-05 | 0.00011 | 0.73 | 0.04184 | 301.3843 |
| rs11249215 | A | G | 1 | 25297184 | 0.1403 | 0.016 | 2.14E-18 | -9.76E-05 | 0.000104 | 0.35 | 0.009815 | 68.41233 |
| rs11581607 | A | G | 1 | 67707690 | -0.3647 | 0.036 | 4.56E-24 | 7.52E-07 | 0.000206 | 1 | 0.016584 | 116.3891 |
| rs11767350 | G | A | 7 | 37385365 | -0.0998 | 0.0164 | 1.19E-09 | 1.17E-05 | 0.000104 | 0.91 | 0.00487 | 33.77725 |
| rs11795343 | C | T | 9 | 32523737 | -0.1062 | 0.0164 | 1.02E-10 | -0.00013 | 0.000106 | 0.2 | 0.00542 | 37.61149 |
| rs118002009 | T | G | 11 | 64124980 | -0.102 | 0.0169 | 1.62E-09 | 0.000114 | 0.000104 | 0.27 | 0.005145 | 35.69435 |
| rs12133684 | A | G | 1 | 2.07E+08 | 0.1218 | 0.0206 | 3.26E-09 | -0.0001 | 0.000131 | 0.42 | 0.004754 | 32.96477 |
| rs12188300 | T | A | 5 | 1.59E+08 | 0.5005 | 0.0264 | 5.39E-80 | 1.34E-05 | 0.000177 | 0.94 | 0.042429 | 305.8153 |
| rs12211087 | A | T | 6 | 31269946 | 1.3769 | 0.0253 | 1.00E-200 | 2.71E-06 | 0.00018 | 0.99 | 0.312191 | 3132.7 |
| rs1295685 | G | A | 5 | 1.32E+08 | 0.1759 | 0.0206 | 1.16E-17 | -1.33E-05 | 0.000134 | 0.92 | 0.009166 | 63.84863 |
| rs131656 | A | G | 22 | 21917450 | 0.114 | 0.0202 | 1.69E-08 | -7.31E-05 | 0.000131 | 0.58 | 0.004027 | 27.90917 |
| rs142502677 | A | T | 18 | 51791307 | 0.1035 | 0.0185 | 2.22E-08 | -1.14E-05 | 0.000118 | 0.92 | 0.00425 | 29.46032 |
| rs1611236 | A | G | 6 | 29748690 | 0.123 | 0.0172 | 7.90E-13 | -3.66E-06 | 0.00011 | 0.97 | 0.006625 | 46.02865 |
| rs1648153 | A | G | 11 | 1.1E+08 | -0.1388 | 0.0164 | 2.93E-17 | -0.00023 | 0.000104 | 0.029 | 0.009405 | 65.52842 |
| rs2021511 | T | C | 16 | 11344903 | -0.1163 | 0.0181 | 1.20E-10 | 3.87E-05 | 0.000115 | 0.74 | 0.005427 | 37.65984 |
| rs2066819 | T | C | 12 | 56750204 | -0.3283 | 0.0348 | 4.28E-21 | 2.67E-05 | 0.000208 | 0.9 | 0.013292 | 92.97828 |
| rs2111485 | G | A | 2 | 1.63E+08 | 0.1616 | 0.0166 | 1.76E-22 | -0.00017 | 0.000105 | 0.1 | 0.012453 | 87.0317 |
| rs2451258 | T | C | 6 | 1.6E+08 | -0.0985 | 0.0169 | 5.26E-09 | -0.00013 | 0.000107 | 0.21 | 0.004536 | 31.44656 |
| rs2638281 | G | A | 19 | 49210869 | 0.0976 | 0.0164 | 2.46E-09 | 8.24E-05 | 0.000105 | 0.43 | 0.004561 | 31.62316 |
| rs2675662 | G | A | 10 | 75599127 | -0.1184 | 0.0167 | 1.49E-12 | -0.00013 | 0.000104 | 0.2 | 0.006894 | 47.91435 |
| rs2735009 | A | G | 6 | 29807853 | -0.2952 | 0.0204 | 1.63E-47 | -0.00013 | 0.000127 | 0.3 | 0.028586 | 203.1053 |
| rs28510484 | C | G | 15 | 31637569 | -0.1398 | 0.0231 | 1.37E-09 | -7.71E-05 | 0.000138 | 0.58 | 0.005516 | 38.27917 |
| rs28752856 | G | C | 6 | 31298421 | 1.1163 | 0.0225 | 1.00E-200 | 0.000122 | 0.000162 | 0.450001 | 0.260145 | 2426.807 |
| rs28998802 | A | G | 17 | 26124908 | 0.2113 | 0.0222 | 1.89E-21 | 0.000151 | 0.00015 | 0.31 | 0.010793 | 75.30496 |
| rs34536443 | C | G | 19 | 10463118 | -0.6799 | 0.0504 | 2.02E-41 | 0.000343 | 0.000256 | 0.18 | 0.039836 | 286.3492 |
| rs35741374 | T | C | 2 | 61072567 | 0.1634 | 0.0163 | 1.08E-23 | 6.49E-05 | 0.000104 | 0.53 | 0.013091 | 91.55295 |
| rs3906814 | G | C | 3 | 17011474 | -0.1144 | 0.0161 | 1.30E-12 | -6.88E-05 | 0.000103 | 0.51 | 0.006543 | 45.45383 |
| rs39841 | A | G | 5 | 96120170 | -0.1624 | 0.0176 | 2.56E-20 | -4.93E-05 | 0.000116 | 0.67 | 0.010448 | 72.8701 |
| rs438650 | C | T | 1 | 8290082 | 0.1244 | 0.0195 | 1.76E-10 | 0.000181 | 0.000124 | 0.14 | 0.005463 | 37.9125 |
| rs4712520 | C | T | 6 | 20640871 | 0.1301 | 0.0212 | 8.27E-10 | -6.55E-05 | 0.000133 | 0.62 | 0.005064 | 35.13028 |
| rs4889526 | A | C | 16 | 31030344 | 0.1216 | 0.0165 | 1.62E-13 | 8.11E-05 | 0.000107 | 0.450001 | 0.006924 | 48.12185 |
| rs4978343 | T | G | 9 | 1.11E+08 | -0.0988 | 0.0164 | 1.77E-09 | 1.52E-06 | 0.000105 | 0.99 | 0.00472 | 32.73382 |
| rs55868394 | A | C | 17 | 73851113 | 0.1389 | 0.0246 | 1.71E-08 | -9.01E-05 | 0.000157 | 0.57 | 0.004256 | 29.4992 |
| rs559406 | T | G | 18 | 12857002 | -0.0924 | 0.0161 | 1.02E-08 | 6.33E-05 | 0.000104 | 0.54 | 0.004232 | 29.33314 |
| rs582757 | T | C | 6 | 1.38E+08 | -0.1846 | 0.0176 | 1.15E-25 | -0.00019 | 0.000116 | 0.099001 | 0.013564 | 94.90488 |
| rs59960858 | A | C | 1 | 24519437 | -0.2086 | 0.0243 | 1.06E-17 | -0.00014 | 0.000151 | 0.34 | 0.010127 | 70.60999 |
| rs6894840 | G | T | 5 | 1.59E+08 | 0.1152 | 0.0166 | 3.42E-12 | -3.45E-05 | 0.000106 | 0.75 | 0.00627 | 43.54861 |
| rs7141014 | C | T | 14 | 98667928 | -0.1123 | 0.0203 | 3.07E-08 | 2.42E-06 | 0.000127 | 0.98 | 0.004173 | 28.92036 |
| rs73986523 | C | T | 17 | 40302271 | -0.2122 | 0.038 | 2.41E-08 | -0.00016 | 0.000216 | 0.450001 | 0.005164 | 35.8237 |
| rs771576 | C | T | 3 | 1.02E+08 | 0.1029 | 0.0169 | 1.08E-09 | 8.49E-05 | 0.000106 | 0.42 | 0.005001 | 34.6863 |
| rs8016947 | G | T | 14 | 35832666 | 0.1476 | 0.0162 | 8.18E-20 | -4.20E-05 | 0.000104 | 0.69 | 0.010726 | 74.83178 |
| rs9258357 | C | T | 6 | 29751129 | 0.2839 | 0.0233 | 3.48E-34 | 1.69E-05 | 0.00014 | 0.9 | 0.021924 | 154.7057 |
| rs9264277 | C | T | 6 | 31224667 | 0.3705 | 0.018 | 1.91E-94 | 5.54E-05 | 0.000107 | 0.61 | 0.06372 | 469.719 |
| rs9277939 | A | C | 6 | 33189873 | 0.228 | 0.0253 | 2.16E-19 | -6.37E-05 | 0.000176 | 0.719999 | 0.00905 | 63.03508 |
| rs9468618 | T | C | 6 | 29750776 | -0.1856 | 0.03 | 6.49E-10 | 0.000207 | 0.000181 | 0.25 | 0.005618 | 38.99182 |
| rs9481169 | T | G | 6 | 1.12E+08 | 0.3716 | 0.0263 | 2.47E-45 | -0.00023 | 0.000185 | 0.21 | 0.021615 | 152.4779 |
| rs9513593 | A | G | 13 | 99950260 | -0.1197 | 0.0205 | 5.33E-09 | -0.00016 | 0.000138 | 0.24 | 0.004004 | 27.74499 |
| rs957970 | G | A | 17 | 40519890 | 0.0929 | 0.0168 | 3.05E-08 | -0.00012 | 0.000108 | 0.28 | 0.003925 | 27.19515 |
| rs9591325 | C | T | 13 | 50811220 | -0.19 | 0.0324 | 4.50E-09 | -0.00017 | 0.000205 | 0.4 | 0.004575 | 31.72378 |
| rs9695923 | T | C | 9 | 1.11E+08 | -0.0955 | 0.0165 | 7.08E-09 | -1.33E-05 | 0.000105 | 0.9 | 0.004389 | 30.42525 |

Supplementary Table 9. Genetic instrumental tools used in Mendelian randomization analysis on psoriasis and liver cancer, based on Stuart PE et al.

| SNP | Effect allele | Other allele | Chr | Pos | Exposure | | | Outcome | | | R^2^ | F |
| --- | --- | --- | --- | --- | --- | --- | --- | --- | --- | --- | --- | --- |
|  |  |  |  |  | beta | se | pval | beta | se | pval |  |  |
| rs10888503 | T | C | 1 | 1.53E+08 | 0.1866 | 0.0171 | 1.44E-27 | 2.89E-05 | 5.20E-05 | 0.58 | 0.015673 | 120.9241 |
| rs10893884 | C | T | 11 | 1.28E+08 | -0.106 | 0.016 | 3.99E-11 | -6.17E-05 | 4.94E-05 | 0.21 | 0.005614 | 42.88005 |
| rs11085744 | T | C | 19 | 10819967 | 0.1146 | 0.0166 | 5.21E-12 | -3.07E-05 | 4.98E-05 | 0.54 | 0.006475 | 49.49911 |
| rs1108618 | G | A | 10 | 81043743 | -0.1098 | 0.0165 | 3.25E-11 | 3.90E-05 | 4.98E-05 | 0.43 | 0.005911 | 45.15846 |
| rs11135059 | A | G | 5 | 1.59E+08 | -0.3078 | 0.0178 | 4.41E-67 | 7.43E-05 | 5.25E-05 | 0.16 | 0.041841 | 331.6368 |
| rs11249215 | A | G | 1 | 25297184 | 0.1403 | 0.016 | 2.14E-18 | -1.71E-05 | 4.96E-05 | 0.73 | 0.009815 | 75.27881 |
| rs11767350 | G | A | 7 | 37385365 | -0.0998 | 0.0164 | 1.19E-09 | 4.45E-05 | 4.98E-05 | 0.37 | 0.00487 | 37.16743 |
| rs11795343 | C | T | 9 | 32523737 | -0.1062 | 0.0164 | 1.02E-10 | 4.19E-05 | 5.04E-05 | 0.41 | 0.00542 | 41.3878 |
| rs118002009 | T | G | 11 | 64124980 | -0.102 | 0.0169 | 1.62E-09 | 2.09E-05 | 4.95E-05 | 0.67 | 0.005145 | 39.27639 |
| rs12133684 | A | G | 1 | 2.07E+08 | 0.1218 | 0.0206 | 3.26E-09 | 2.00E-05 | 6.24E-05 | 0.75 | 0.004754 | 36.2757 |
| rs12188300 | T | A | 5 | 1.59E+08 | 0.5005 | 0.0264 | 5.39E-80 | -0.0001 | 8.46E-05 | 0.23 | 0.042425 | 336.4711 |
| rs12211087 | A | T | 6 | 31269946 | 1.3769 | 0.0253 | 1.00E-200 | 4.18E-05 | 8.58E-05 | 0.630001 | 0.312203 | 3447.31 |
| rs1295685 | G | A | 5 | 1.32E+08 | 0.1759 | 0.0206 | 1.16E-17 | -3.99E-06 | 6.39E-05 | 0.95 | 0.009166 | 70.25675 |
| rs131656 | A | G | 22 | 21917450 | 0.114 | 0.0202 | 1.69E-08 | -1.88E-05 | 6.25E-05 | 0.760001 | 0.004028 | 30.71148 |
| rs142502677 | A | T | 18 | 51791307 | 0.1035 | 0.0185 | 2.22E-08 | 3.05E-06 | 5.63E-05 | 0.96 | 0.00425 | 32.41723 |
| rs1611236 | A | G | 6 | 29748690 | 0.123 | 0.0172 | 7.90E-13 | 2.45E-05 | 5.27E-05 | 0.64 | 0.006625 | 50.64886 |
| rs1648153 | A | G | 11 | 1.1E+08 | -0.1388 | 0.0164 | 2.93E-17 | 4.18E-05 | 4.98E-05 | 0.4 | 0.009405 | 72.10807 |
| rs2021511 | T | C | 16 | 11344903 | -0.1163 | 0.0181 | 1.20E-10 | -2.79E-05 | 5.49E-05 | 0.61 | 0.005427 | 41.43834 |
| rs2111485 | G | A | 2 | 1.63E+08 | 0.1616 | 0.0166 | 1.76E-22 | 4.84E-05 | 5.04E-05 | 0.34 | 0.012452 | 95.7621 |
| rs2451258 | T | C | 6 | 1.6E+08 | -0.0985 | 0.0169 | 5.26E-09 | -3.42E-05 | 5.10E-05 | 0.5 | 0.004535 | 34.60176 |
| rs2638281 | G | A | 19 | 49210869 | 0.0976 | 0.0164 | 2.46E-09 | 5.35E-05 | 5.03E-05 | 0.29 | 0.004561 | 34.79726 |
| rs2675662 | G | A | 10 | 75599127 | -0.1184 | 0.0167 | 1.49E-12 | 3.81E-06 | 4.97E-05 | 0.94 | 0.006894 | 52.72412 |
| rs2735009 | A | G | 6 | 29807853 | -0.2952 | 0.0204 | 1.63E-47 | -6.76E-05 | 6.07E-05 | 0.27 | 0.028588 | 223.5005 |
| rs28510484 | C | G | 15 | 31637569 | -0.1398 | 0.0231 | 1.37E-09 | -5.02E-05 | 6.57E-05 | 0.44 | 0.005516 | 42.12163 |
| rs28752856 | G | C | 6 | 31298421 | 1.1163 | 0.0225 | 1.00E-200 | 0.000184 | 7.74E-05 | 0.018 | 0.260158 | 2670.556 |
| rs28998802 | A | G | 17 | 26124908 | 0.2113 | 0.0222 | 1.89E-21 | -2.83E-05 | 7.17E-05 | 0.69 | 0.010792 | 82.85193 |
| rs35741374 | T | C | 2 | 61072567 | 0.1634 | 0.0163 | 1.08E-23 | -4.70E-05 | 4.98E-05 | 0.35 | 0.013092 | 100.7432 |
| rs3906814 | G | C | 3 | 17011474 | -0.1144 | 0.0161 | 1.30E-12 | 8.75E-05 | 4.93E-05 | 0.075999 | 0.006543 | 50.01566 |
| rs39841 | A | G | 5 | 96120170 | -0.1624 | 0.0176 | 2.56E-20 | 0.000103 | 5.53E-05 | 0.063 | 0.010447 | 80.17806 |
| rs438650 | C | T | 1 | 8290082 | 0.1244 | 0.0195 | 1.76E-10 | 4.73E-07 | 5.91E-05 | 0.99 | 0.005463 | 41.71331 |
| rs4712520 | C | T | 6 | 20640871 | 0.1301 | 0.0212 | 8.27E-10 | -0.0001 | 6.36E-05 | 0.11 | 0.005064 | 38.65677 |
| rs4889526 | A | C | 16 | 31030344 | 0.1216 | 0.0165 | 1.62E-13 | -4.50E-05 | 5.09E-05 | 0.38 | 0.006924 | 52.94985 |
| rs4978343 | T | G | 9 | 1.11E+08 | -0.0988 | 0.0164 | 1.77E-09 | 5.98E-05 | 5.03E-05 | 0.23 | 0.00472 | 36.01951 |
| rs55868394 | A | C | 17 | 73851113 | 0.1389 | 0.0246 | 1.71E-08 | 0.00015 | 7.48E-05 | 0.045 | 0.004257 | 32.46561 |
| rs559406 | T | G | 18 | 12857002 | -0.0924 | 0.0161 | 1.02E-08 | 9.01E-05 | 4.95E-05 | 0.069 | 0.004232 | 32.27707 |
| rs582757 | T | C | 6 | 1.38E+08 | -0.1846 | 0.0176 | 1.15E-25 | 0.000102 | 5.52E-05 | 0.065 | 0.013562 | 104.4158 |
| rs59960858 | A | C | 1 | 24519437 | -0.2086 | 0.0243 | 1.06E-17 | -3.57E-05 | 7.21E-05 | 0.62 | 0.010128 | 77.70312 |
| rs6894840 | G | T | 5 | 1.59E+08 | 0.1152 | 0.0166 | 3.42E-12 | 2.39E-05 | 5.08E-05 | 0.64 | 0.00627 | 47.91867 |
| rs7141014 | C | T | 14 | 98667928 | -0.1123 | 0.0203 | 3.07E-08 | 9.04E-05 | 6.08E-05 | 0.14 | 0.004173 | 31.8245 |
| rs771576 | C | T | 3 | 1.02E+08 | 0.1029 | 0.0169 | 1.08E-09 | -3.24E-06 | 5.08E-05 | 0.95 | 0.005001 | 38.16845 |
| rs8016947 | G | T | 14 | 35832666 | 0.1476 | 0.0162 | 8.18E-20 | -4.81E-05 | 4.96E-05 | 0.33 | 0.010726 | 82.34229 |
| rs9258357 | C | T | 6 | 29751129 | 0.2839 | 0.0233 | 3.48E-34 | -3.43E-05 | 6.67E-05 | 0.61 | 0.021924 | 170.2387 |
| rs9264277 | C | T | 6 | 31224667 | 0.3705 | 0.018 | 1.91E-94 | -1.82E-05 | 5.12E-05 | 0.719999 | 0.063721 | 516.8715 |
| rs9277939 | A | C | 6 | 33189873 | 0.228 | 0.0253 | 2.16E-19 | -0.00013 | 8.40E-05 | 0.12 | 0.00905 | 69.3583 |
| rs9468618 | T | C | 6 | 29750776 | -0.1856 | 0.03 | 6.49E-10 | -0.00012 | 8.63E-05 | 0.18 | 0.005616 | 42.89348 |
| rs9481169 | T | G | 6 | 1.12E+08 | 0.3716 | 0.0263 | 2.47E-45 | 5.67E-05 | 8.82E-05 | 0.52 | 0.02162 | 167.823 |
| rs9513593 | A | G | 13 | 99950260 | -0.1197 | 0.0205 | 5.33E-09 | 2.12E-05 | 6.59E-05 | 0.75 | 0.004003 | 30.5261 |
| rs957970 | G | A | 17 | 40519890 | 0.0929 | 0.0168 | 3.05E-08 | 0.000116 | 5.16E-05 | 0.024 | 0.003925 | 29.92668 |
| rs9695923 | T | C | 9 | 1.11E+08 | -0.0955 | 0.0165 | 7.08E-09 | 9.24E-05 | 5.04E-05 | 0.065999 | 0.004389 | 33.47956 |

Supplementary Table 10. Genetic instrumental tools used in Mendelian randomization analysis on psoriasis and bile duct cancer, based on Stuart PE et al.

| SNP | Effect allele | Other allele | Chr | Pos | Exposure | | | Outcome | | | R^2^ | F |
| --- | --- | --- | --- | --- | --- | --- | --- | --- | --- | --- | --- | --- |
|  |  |  |  |  | beta | se | pval | beta | se | pval |  |  |
| rs10888503 | T | C | 1 | 1.53E+08 | 0.1866 | 0.0171 | 1.44E-27 | 1.38E-05 | 7.51E-05 | 0.85 | 0.015673 | 109.7801 |
| rs10893884 | C | T | 11 | 1.28E+08 | -0.106 | 0.016 | 3.99E-11 | -2.37E-05 | 7.13E-05 | 0.74 | 0.005614 | 38.92815 |
| rs11085744 | T | C | 19 | 10819967 | 0.1146 | 0.0166 | 5.21E-12 | -0.00014 | 7.18E-05 | 0.06 | 0.006476 | 44.93778 |
| rs1108618 | G | A | 10 | 81043743 | -0.1098 | 0.0165 | 3.25E-11 | -3.64E-05 | 7.18E-05 | 0.61 | 0.005911 | 40.99619 |
| rs11135059 | A | G | 5 | 1.59E+08 | -0.3078 | 0.0178 | 4.41E-67 | 8.93E-05 | 7.57E-05 | 0.24 | 0.041841 | 301.0743 |
| rs11249215 | A | G | 1 | 25297184 | 0.1403 | 0.016 | 2.14E-18 | -7.46E-05 | 7.15E-05 | 0.3 | 0.009815 | 68.34083 |
| rs11581607 | A | G | 1 | 67707690 | -0.3647 | 0.036 | 4.56E-24 | -8.28E-05 | 0.000142 | 0.56 | 0.016582 | 116.2575 |
| rs11767350 | G | A | 7 | 37385365 | -0.0998 | 0.0164 | 1.19E-09 | 2.58E-05 | 7.18E-05 | 0.719999 | 0.00487 | 33.74194 |
| rs11795343 | C | T | 9 | 32523737 | -0.1062 | 0.0164 | 1.02E-10 | 1.06E-05 | 7.27E-05 | 0.88 | 0.00542 | 37.57322 |
| rs118002009 | T | G | 11 | 64124980 | -0.102 | 0.0169 | 1.62E-09 | 0.00013 | 7.14E-05 | 0.069 | 0.005145 | 35.65705 |
| rs12133684 | A | G | 1 | 2.07E+08 | 0.1218 | 0.0206 | 3.26E-09 | 0.000124 | 9.00E-05 | 0.17 | 0.004754 | 32.93448 |
| rs12188300 | T | A | 5 | 1.59E+08 | 0.5005 | 0.0264 | 5.39E-80 | -5.57E-05 | 0.000122 | 0.649999 | 0.042426 | 305.4738 |
| rs12211087 | A | T | 6 | 31269946 | 1.3769 | 0.0253 | 1.00E-200 | 0.000188 | 0.000124 | 0.13 | 0.31224 | 3130.146 |
| rs1295685 | G | A | 5 | 1.32E+08 | 0.1759 | 0.0206 | 1.16E-17 | -1.55E-05 | 9.21E-05 | 0.87 | 0.009166 | 63.78237 |
| rs131656 | A | G | 22 | 21917450 | 0.114 | 0.0202 | 1.69E-08 | 4.48E-05 | 9.02E-05 | 0.62 | 0.004028 | 27.88219 |
| rs142502677 | TTG | T | 18 | 51791307 | 0.1035 | 0.0185 | 2.22E-08 | 1.84E-05 | 8.12E-05 | 0.82 | 0.00425 | 29.42983 |
| rs1611236 | A | G | 6 | 29748690 | 0.123 | 0.0172 | 7.90E-13 | 2.96E-05 | 7.60E-05 | 0.7 | 0.006625 | 45.98108 |
| rs1648153 | A | G | 11 | 1.1E+08 | -0.1388 | 0.0164 | 2.93E-17 | -3.66E-05 | 7.18E-05 | 0.61 | 0.009405 | 65.46173 |
| rs2021511 | T | C | 16 | 11344903 | -0.1163 | 0.0181 | 1.20E-10 | -4.85E-05 | 7.93E-05 | 0.54 | 0.005427 | 37.619 |
| rs2066819 | T | C | 12 | 56750204 | -0.3283 | 0.0348 | 4.28E-21 | -0.00023 | 0.000143 | 0.11 | 0.013289 | 92.85846 |
| rs2111485 | G | A | 2 | 1.63E+08 | 0.1616 | 0.0166 | 1.76E-22 | 5.82E-05 | 7.27E-05 | 0.42 | 0.012452 | 86.93633 |
| rs2451258 | T | C | 6 | 1.6E+08 | -0.0985 | 0.0169 | 5.26E-09 | 2.00E-05 | 7.36E-05 | 0.79 | 0.004535 | 31.41241 |
| rs2638281 | G | A | 19 | 49210869 | 0.0976 | 0.0164 | 2.46E-09 | 6.01E-05 | 7.26E-05 | 0.41 | 0.004561 | 31.59026 |
| rs2675662 | G | A | 10 | 75599127 | -0.1184 | 0.0167 | 1.49E-12 | -6.65E-07 | 7.17E-05 | 0.99 | 0.006894 | 47.86499 |
| rs2735009 | A | G | 6 | 29807853 | -0.2952 | 0.0204 | 1.63E-47 | -0.00013 | 8.76E-05 | 0.13 | 0.028587 | 202.8943 |
| rs28510484 | C | G | 15 | 31637569 | -0.1398 | 0.0231 | 1.37E-09 | -3.49E-05 | 9.48E-05 | 0.709999 | 0.005516 | 38.24019 |
| rs28752856 | G | C | 6 | 31298421 | 1.1163 | 0.0225 | 1.00E-200 | 0.000107 | 0.000112 | 0.34 | 0.260143 | 2424.243 |
| rs28998802 | A | G | 17 | 26124908 | 0.2113 | 0.0222 | 1.89E-21 | -2.93E-05 | 0.000103 | 0.780001 | 0.010792 | 75.21622 |
| rs34536443 | C | G | 19 | 10463118 | -0.6799 | 0.0504 | 2.02E-41 | -0.00014 | 0.000176 | 0.44 | 0.03982 | 285.9301 |
| rs35741374 | T | C | 2 | 61072567 | 0.1634 | 0.0163 | 1.08E-23 | -2.64E-05 | 7.18E-05 | 0.709999 | 0.013091 | 91.45835 |
| rs3906814 | G | C | 3 | 17011474 | -0.1144 | 0.0161 | 1.30E-12 | 6.38E-05 | 7.11E-05 | 0.37 | 0.006543 | 45.40618 |
| rs39841 | A | G | 5 | 96120170 | -0.1624 | 0.0176 | 2.56E-20 | 4.14E-05 | 7.97E-05 | 0.6 | 0.010447 | 72.7908 |
| rs438650 | C | T | 1 | 8290082 | 0.1244 | 0.0195 | 1.76E-10 | 4.22E-05 | 8.52E-05 | 0.62 | 0.005463 | 37.86979 |
| rs4712520 | C | T | 6 | 20640871 | 0.1301 | 0.0212 | 8.27E-10 | -4.74E-05 | 9.18E-05 | 0.61 | 0.005064 | 35.09292 |
| rs4889526 | A | C | 16 | 31030344 | 0.1216 | 0.0165 | 1.62E-13 | -3.62E-05 | 7.35E-05 | 0.62 | 0.006924 | 48.07004 |
| rs4978343 | T | G | 9 | 1.11E+08 | -0.0988 | 0.0164 | 1.77E-09 | 0.000103 | 7.26E-05 | 0.15 | 0.00472 | 32.70016 |
| rs55868394 | A | C | 17 | 73851113 | 0.1389 | 0.0246 | 1.71E-08 | 0.000122 | 0.000108 | 0.26 | 0.004257 | 29.47295 |
| rs559406 | T | G | 18 | 12857002 | -0.0924 | 0.0161 | 1.02E-08 | 0.000108 | 7.14E-05 | 0.13 | 0.004232 | 29.30233 |
| rs582757 | T | C | 6 | 1.38E+08 | -0.1846 | 0.0176 | 1.15E-25 | 4.61E-05 | 7.96E-05 | 0.56 | 0.013563 | 94.7951 |
| rs59960858 | A | C | 1 | 24519437 | -0.2086 | 0.0243 | 1.06E-17 | 6.94E-05 | 0.000104 | 0.5 | 0.010129 | 70.54729 |
| rs6894840 | G | T | 5 | 1.59E+08 | 0.1152 | 0.0166 | 3.42E-12 | 7.99E-05 | 7.33E-05 | 0.28 | 0.00627 | 43.50187 |
| rs7141014 | C | T | 14 | 98667928 | -0.1123 | 0.0203 | 3.07E-08 | -6.07E-05 | 8.77E-05 | 0.49 | 0.004173 | 28.88897 |
| rs73986523 | C | T | 17 | 40302271 | -0.2122 | 0.038 | 2.41E-08 | -6.54E-05 | 0.000149 | 0.66 | 0.005164 | 35.78897 |
| rs771576 | C | T | 3 | 1.02E+08 | 0.1029 | 0.0169 | 1.08E-09 | 1.15E-05 | 7.32E-05 | 0.88 | 0.005001 | 34.65067 |
| rs8016947 | G | T | 14 | 35832666 | 0.1476 | 0.0162 | 8.18E-20 | -1.00E-04 | 7.16E-05 | 0.16 | 0.010726 | 74.75404 |
| rs9258357 | C | T | 6 | 29751129 | 0.2839 | 0.0233 | 3.48E-34 | 0.000104 | 9.62E-05 | 0.28 | 0.021922 | 154.5344 |
| rs9264277 | C | T | 6 | 31224667 | 0.3705 | 0.018 | 1.91E-94 | -7.32E-05 | 7.38E-05 | 0.32 | 0.063722 | 469.2437 |
| rs9277939 | A | C | 6 | 33189873 | 0.228 | 0.0253 | 2.16E-19 | -0.00015 | 0.000121 | 0.22 | 0.00905 | 62.96499 |
| rs9468618 | T | C | 6 | 29750776 | -0.1856 | 0.03 | 6.49E-10 | -6.04E-05 | 0.000124 | 0.630001 | 0.005616 | 38.94234 |
| rs9481169 | T | G | 6 | 1.12E+08 | 0.3716 | 0.0263 | 2.47E-45 | -4.90E-05 | 0.000127 | 0.7 | 0.021618 | 152.3431 |
| rs9513593 | A | G | 13 | 99950260 | -0.1197 | 0.0205 | 5.33E-09 | 4.10E-05 | 9.51E-05 | 0.67 | 0.004003 | 27.71239 |
| rs957970 | G | A | 17 | 40519890 | 0.0929 | 0.0168 | 3.05E-08 | 0.000209 | 7.45E-05 | 0.0049 | 0.003925 | 27.1694 |
| rs9591325 | C | T | 13 | 50811220 | -0.19 | 0.0324 | 4.50E-09 | 9.86E-05 | 0.000141 | 0.49 | 0.004576 | 31.69796 |
| rs9695923 | T | C | 9 | 1.11E+08 | -0.0955 | 0.0165 | 7.08E-09 | 0.000111 | 7.26E-05 | 0.13 | 0.004389 | 30.39415 |

Supplementary Table 11. Genetic instrumental tools used in Mendelian randomization analysis on psoriasis and pancreatic cancer, based on Stuart PE et al.

| SNP | Effect allele | Other allele | Chr | Pos | Exposure | | | Outcome | | |
| --- | --- | --- | --- | --- | --- | --- | --- | --- | --- | --- |
|  |  |  |  |  | beta | se | pval | beta | se | pval |
| rs11085744 | T | C | 19 | 10840201 | 0.1146 | 0.0166 | 5.21E-12 | 0.057841 | 0.048603 | 0.234345 |
| rs11135059 | A | G | 5 | 1.59E+08 | -0.3078 | 0.0178 | 4.41E-67 | 0.0752 | 0.051475 | 0.144013 |
| rs11249215 | A | G | 1 | 25301869 | 0.1403 | 0.016 | 2.14E-18 | 0.067846 | 0.047512 | 0.153481 |
| rs11795343 | C | T | 9 | 32523737 | -0.1062 | 0.0164 | 1.02E-10 | -0.04531 | 0.048138 | 0.346203 |
| rs118002009 | T | G | 11 | 64125142 | -0.102 | 0.0169 | 1.62E-09 | -0.06369 | 0.048779 | 0.191918 |
| rs12133684 | A | G | 1 | 2.07E+08 | 0.1218 | 0.0206 | 3.26E-09 | -0.0031 | 0.05705 | 0.956376 |
| rs131656 | A | G | 22 | 21939675 | 0.114 | 0.0202 | 1.69E-08 | 0.01784 | 0.058084 | 0.758529 |
| rs1611236 | A | G | 6 | 29776322 | 0.123 | 0.0172 | 7.90E-13 | -0.00321 | 0.053468 | 0.951903 |
| rs1648153 | A | G | 11 | 1.1E+08 | -0.1388 | 0.0164 | 2.93E-17 | -0.01542 | 0.04915 | 0.753033 |
| rs2021511 | T | C | 16 | 11361202 | -0.1163 | 0.0181 | 1.20E-10 | 0.015086 | 0.0523 | 0.77332 |
| rs2111485 | G | A | 2 | 1.63E+08 | 0.1616 | 0.0166 | 1.76E-22 | -0.00688 | 0.048139 | 0.885896 |
| rs2451258 | T | C | 6 | 1.6E+08 | -0.0985 | 0.0169 | 5.26E-09 | 0.025113 | 0.051917 | 0.628125 |
| rs2675662 | G | A | 10 | 75632760 | -0.1184 | 0.0167 | 1.49E-12 | -0.0024 | 0.047776 | 0.959142 |
| rs2735009 | A | G | 6 | 29807923 | -0.2952 | 0.0204 | 1.63E-47 | -0.06433 | 0.057825 | 0.265759 |
| rs35741374 | T | C | 2 | 61081542 | 0.1634 | 0.0163 | 1.08E-23 | -0.0026 | 0.048044 | 0.956827 |
| rs3906814 | G | C | 3 | 17083709 | -0.1144 | 0.0161 | 1.30E-12 | 0.002503 | 0.047781 | 0.957896 |
| rs39841 | A | G | 5 | 96188666 | -0.1624 | 0.0176 | 2.56E-20 | -0.01784 | 0.053703 | 0.739827 |
| rs4712520 | C | T | 6 | 20663335 | 0.1301 | 0.0212 | 8.27E-10 | 0.0494 | 0.059022 | 0.402511 |
| rs4889526 | A | C | 16 | 31102321 | 0.1216 | 0.0165 | 1.62E-13 | 0.016759 | 0.049094 | 0.732556 |
| rs4978343 | T | G | 9 | 1.11E+08 | -0.0988 | 0.0164 | 1.77E-09 | 0.059589 | 0.048117 | 0.215583 |
| rs8016947 | G | T | 14 | 35832666 | 0.1476 | 0.0162 | 8.18E-20 | 0.070423 | 0.047695 | 0.139741 |
| rs9504361 | G | A | 6 | 577820 | -0.1068 | 0.0164 | 6.44E-11 | 0.056947 | 0.04714 | 0.227076 |
| rs9513593 | A | G | 13 | 1E+08 | -0.1197 | 0.0205 | 5.33E-09 | 0.065286 | 0.061424 | 0.2875 |

Supplementary Table 12. Genetic instrumental tools used in Mendelian randomization analysis on psoriasis and colorectal cancer, based on Stuart PE et al.

| SNP | Effect allele | Other allele | Chr | Pos | Exposure | | | Outcome | | | R^2^ | F |
| --- | --- | --- | --- | --- | --- | --- | --- | --- | --- | --- | --- | --- |
|  |  |  |  |  | beta | se | pval | beta | se | pval |  |  |
| rs10888503 | T | C | 1 | 1.53E+08 | 0.1866 | 0.0171 | 1.44E-27 | 0.000109 | 0.000295 | 0.709999 | 0.015672 | 109.3151 |
| rs10893884 | C | T | 11 | 1.28E+08 | -0.106 | 0.016 | 3.99E-11 | 0.00076 | 0.00028 | 0.0068 | 0.005614 | 38.76426 |
| rs11085744 | T | C | 19 | 10819967 | 0.1146 | 0.0166 | 5.21E-12 | 0.00022 | 0.000283 | 0.44 | 0.006475 | 44.74771 |
| rs1108618 | G | A | 10 | 81043743 | -0.1098 | 0.0165 | 3.25E-11 | 0.000465 | 0.000282 | 0.1 | 0.005911 | 40.82778 |
| rs11135059 | A | G | 5 | 1.59E+08 | -0.3078 | 0.0178 | 4.41E-67 | -0.00073 | 0.000298 | 0.014 | 0.041829 | 299.7277 |
| rs11249215 | A | G | 1 | 25297184 | 0.1403 | 0.016 | 2.14E-18 | -0.00011 | 0.000281 | 0.69 | 0.009815 | 68.05441 |
| rs115059666 | A | G | 5 | 1.5E+08 | 0.5147 | 0.0744 | 4.47E-12 | 5.03E-05 | 0.001328 | 0.97 | 0.005997 | 41.4196 |
| rs11581607 | A | G | 1 | 67707690 | -0.3647 | 0.036 | 4.56E-24 | -0.00039 | 0.00056 | 0.49 | 0.016578 | 115.7409 |
| rs11767350 | G | A | 7 | 37385365 | -0.0998 | 0.0164 | 1.19E-09 | 0.000181 | 0.000283 | 0.52 | 0.00487 | 33.60153 |
| rs11795343 | C | T | 9 | 32523737 | -0.1062 | 0.0164 | 1.02E-10 | -0.00028 | 0.000286 | 0.32 | 0.00542 | 37.4133 |
| rs118002009 | T | G | 11 | 64124980 | -0.102 | 0.0169 | 1.62E-09 | 0.00043 | 0.000281 | 0.13 | 0.005145 | 35.50883 |
| rs12133684 | A | G | 1 | 2.07E+08 | 0.1218 | 0.0206 | 3.26E-09 | -2.86E-05 | 0.000354 | 0.94 | 0.004754 | 32.79392 |
| rs12188300 | T | A | 5 | 1.59E+08 | 0.5005 | 0.0264 | 5.39E-80 | -0.00026 | 0.000481 | 0.59 | 0.042422 | 304.1605 |
| rs12211087 | A | T | 6 | 31269946 | 1.3769 | 0.0253 | 1.00E-200 | 0.000651 | 0.000487 | 0.18 | 0.312365 | 3118.835 |
| rs1295685 | G | A | 5 | 1.32E+08 | 0.1759 | 0.0206 | 1.16E-17 | 0.000644 | 0.000363 | 0.075999 | 0.009162 | 63.48477 |
| rs131656 | A | G | 22 | 21917450 | 0.114 | 0.0202 | 1.69E-08 | 0.000112 | 0.000355 | 0.75 | 0.004028 | 27.76505 |
| rs142502677 | A | T | 18 | 51791307 | 0.1035 | 0.0185 | 2.22E-08 | -8.68E-05 | 0.00032 | 0.79 | 0.00425 | 29.30526 |
| rs1611236 | A | G | 6 | 29748690 | 0.123 | 0.0172 | 7.90E-13 | 0.000658 | 0.000299 | 0.028 | 0.006626 | 45.79905 |
| rs1648153 | A | G | 11 | 1.1E+08 | -0.1388 | 0.0164 | 2.93E-17 | 0.000282 | 0.000283 | 0.32 | 0.009406 | 65.19117 |
| rs2021511 | T | C | 16 | 11344903 | -0.1163 | 0.0181 | 1.20E-10 | -0.0001 | 0.000312 | 0.75 | 0.005426 | 37.45998 |
| rs2066819 | T | C | 12 | 56750204 | -0.3283 | 0.0348 | 4.28E-21 | 0.000327 | 0.000563 | 0.56 | 0.013297 | 92.52618 |
| rs2111485 | G | A | 2 | 1.63E+08 | 0.1616 | 0.0166 | 1.76E-22 | -0.00019 | 0.000286 | 0.5 | 0.012453 | 86.57584 |
| rs2451258 | T | C | 6 | 1.6E+08 | -0.0985 | 0.0169 | 5.26E-09 | -5.33E-05 | 0.00029 | 0.85 | 0.004536 | 31.2817 |
| rs2638281 | G | A | 19 | 49210869 | 0.0976 | 0.0164 | 2.46E-09 | 0.000672 | 0.000286 | 0.019 | 0.00456 | 31.45334 |
| rs2675662 | G | A | 10 | 75599127 | -0.1184 | 0.0167 | 1.49E-12 | -0.00027 | 0.000282 | 0.33 | 0.006894 | 47.66296 |
| rs2735009 | A | G | 6 | 29807853 | -0.2952 | 0.0204 | 1.63E-47 | 0.0003 | 0.000345 | 0.38 | 0.028592 | 202.0807 |
| rs28510484 | C | G | 15 | 31637569 | -0.1398 | 0.0231 | 1.37E-09 | -0.00037 | 0.000373 | 0.32 | 0.005515 | 38.07175 |
| rs28752856 | G | C | 6 | 31298421 | 1.1163 | 0.0225 | 1.00E-200 | -0.00132 | 0.00044 | 0.0027 | 0.259848 | 2410.39 |
| rs28998802 | A | G | 17 | 26124908 | 0.2113 | 0.0222 | 1.89E-21 | 0.000464 | 0.000407 | 0.25 | 0.010796 | 74.92947 |
| rs34536443 | C | G | 19 | 10463118 | -0.6799 | 0.0504 | 2.02E-41 | 0.000767 | 0.000694 | 0.27 | 0.039852 | 284.9702 |
| rs35741374 | T | C | 2 | 61072567 | 0.1634 | 0.0163 | 1.08E-23 | 0.00032 | 0.000283 | 0.26 | 0.013091 | 91.07053 |
| rs3906814 | G | C | 3 | 17011474 | -0.1144 | 0.0161 | 1.30E-12 | -0.00029 | 0.00028 | 0.31 | 0.006543 | 45.21617 |
| rs39841 | A | G | 5 | 96120170 | -0.1624 | 0.0176 | 2.56E-20 | -1.35E-05 | 0.000314 | 0.97 | 0.010447 | 72.48652 |
| rs438650 | C | T | 1 | 8290082 | 0.1244 | 0.0195 | 1.76E-10 | 0.000225 | 0.000335 | 0.5 | 0.005463 | 37.7158 |
| rs4712520 | C | T | 6 | 20640871 | 0.1301 | 0.0212 | 8.27E-10 | 0.000271 | 0.000361 | 0.450001 | 0.005063 | 34.9398 |
| rs4889526 | A | C | 16 | 31030344 | 0.1216 | 0.0165 | 1.62E-13 | 0.000121 | 0.000289 | 0.68 | 0.006924 | 47.87104 |
| rs4978343 | T | G | 9 | 1.11E+08 | -0.0988 | 0.0164 | 1.77E-09 | 0.000199 | 0.000286 | 0.49 | 0.004721 | 32.56363 |
| rs55868394 | A | C | 17 | 73851113 | 0.1389 | 0.0246 | 1.71E-08 | 0.000974 | 0.000425 | 0.022 | 0.004259 | 29.36905 |
| rs559406 | T | G | 18 | 12857002 | -0.0924 | 0.0161 | 1.02E-08 | 0.000403 | 0.000281 | 0.15 | 0.004232 | 29.17872 |
| rs582757 | T | C | 6 | 1.38E+08 | -0.1846 | 0.0176 | 1.15E-25 | 0.000731 | 0.000313 | 0.02 | 0.013558 | 94.36733 |
| rs59960858 | A | C | 1 | 24519437 | -0.2086 | 0.0243 | 1.06E-17 | -0.00079 | 0.000409 | 0.055 | 0.010122 | 70.20492 |
| rs6894840 | G | T | 5 | 1.59E+08 | 0.1152 | 0.0166 | 3.42E-12 | -5.72E-05 | 0.000288 | 0.84 | 0.00627 | 43.32127 |
| rs7141014 | C | T | 14 | 98667928 | -0.1123 | 0.0203 | 3.07E-08 | 0.000192 | 0.000345 | 0.58 | 0.004173 | 28.77252 |
| rs73986523 | C | T | 17 | 40302271 | -0.2122 | 0.038 | 2.41E-08 | -0.00055 | 0.000585 | 0.35 | 0.005162 | 35.62422 |
| rs771576 | C | T | 3 | 1.02E+08 | 0.1029 | 0.0169 | 1.08E-09 | 0.000136 | 0.000288 | 0.64 | 0.005 | 34.50374 |
| rs8016947 | G | T | 14 | 35832666 | 0.1476 | 0.0162 | 8.18E-20 | -5.40E-05 | 0.000282 | 0.85 | 0.010726 | 74.44045 |
| rs9258357 | C | T | 6 | 29751129 | 0.2839 | 0.0233 | 3.48E-34 | 0.000855 | 0.000379 | 0.024 | 0.021911 | 153.8089 |
| rs9264277 | C | T | 6 | 31224667 | 0.3705 | 0.018 | 1.91E-94 | -9.39E-06 | 0.000291 | 0.97 | 0.063722 | 467.2772 |
| rs9277939 | A | C | 6 | 33189873 | 0.228 | 0.0253 | 2.16E-19 | -0.00049 | 0.000477 | 0.31 | 0.009047 | 62.68067 |
| rs9468618 | T | C | 6 | 29750776 | -0.1856 | 0.03 | 6.49E-10 | -1.57E-05 | 0.00049 | 0.97 | 0.005617 | 38.78038 |
| rs9481169 | T | G | 6 | 1.12E+08 | 0.3716 | 0.0263 | 2.47E-45 | -0.00116 | 0.000501 | 0.021 | 0.021596 | 151.5489 |
| rs9513593 | A | G | 13 | 99950260 | -0.1197 | 0.0205 | 5.33E-09 | 0.000642 | 0.000374 | 0.086 | 0.004002 | 27.58377 |
| rs957970 | G | A | 17 | 40519890 | 0.0929 | 0.0168 | 3.05E-08 | 0.000143 | 0.000293 | 0.630001 | 0.003925 | 27.05482 |
| rs9591325 | C | T | 13 | 50811220 | -0.19 | 0.0324 | 4.50E-09 | -0.0009 | 0.000557 | 0.11 | 0.004572 | 31.53491 |
| rs9695923 | T | C | 9 | 1.11E+08 | -0.0955 | 0.0165 | 7.08E-09 | 7.19E-05 | 0.000286 | 0.8 | 0.004389 | 30.26642 |

Supplementary Table 13. Genetic instrumental tools used in Mendelian randomization analysis on psoriasis and kidney cancer, based on Stuart PE et al.

| SNP | Effect allele | Other allele | Chr | Pos | Exposure | | | Outcome | | | R^2^ | F |
| --- | --- | --- | --- | --- | --- | --- | --- | --- | --- | --- | --- | --- |
|  |  |  |  |  | beta | se | pval | beta | se | pval |  |  |
| rs10888503 | T | C | 1 | 1.53E+08 | 0.1866 | 0.0171 | 1.44E-27 | 4.75E-05 | 0.000108 | 0.66 | 0.015678 | 273.1134 |
| rs10893884 | C | T | 11 | 1.28E+08 | -0.106 | 0.016 | 3.99E-11 | -0.00032 | 0.000102 | 0.0019 | 0.005614 | 96.80906 |
| rs11085744 | T | C | 19 | 10819967 | 0.1146 | 0.0166 | 5.21E-12 | -0.00011 | 0.000103 | 0.29 | 0.006476 | 111.7655 |
| rs1108618 | G | A | 10 | 81043743 | -0.1098 | 0.0165 | 3.25E-11 | -4.33E-05 | 0.000103 | 0.67 | 0.005912 | 101.9703 |
| rs11135059 | A | G | 5 | 1.59E+08 | -0.3078 | 0.0178 | 4.41E-67 | -1.89E-05 | 0.000109 | 0.86 | 0.041834 | 748.6699 |
| rs11249215 | A | G | 1 | 25297184 | 0.1403 | 0.016 | 2.14E-18 | 7.04E-05 | 0.000102 | 0.49 | 0.009814 | 169.9599 |
| rs11767350 | G | A | 7 | 37385365 | -0.0998 | 0.0164 | 1.19E-09 | 3.47E-07 | 0.000103 | 1 | 0.004871 | 83.94115 |
| rs11795343 | C | T | 9 | 32523737 | -0.1062 | 0.0164 | 1.02E-10 | -5.31E-05 | 0.000104 | 0.61 | 0.00542 | 93.43909 |
| rs118002009 | T | G | 11 | 64124980 | -0.102 | 0.0169 | 1.62E-09 | -6.99E-05 | 0.000102 | 0.5 | 0.005146 | 88.69121 |
| rs1611236 | A | G | 6 | 29752278 | 0.123 | 0.0172 | 7.90E-13 | 3.13E-05 | 0.000109 | 0.77 | 0.006632 | 114.4764 |
| rs1648153 | A | G | 11 | 1.1E+08 | -0.1388 | 0.0164 | 2.93E-17 | -2.79E-05 | 0.000103 | 0.79 | 0.009395 | 162.6242 |
| rs2111485 | G | A | 2 | 1.63E+08 | 0.1616 | 0.0166 | 1.76E-22 | -0.00033 | 0.000104 | 0.0016 | 0.012462 | 216.3944 |
| rs2451258 | T | C | 6 | 1.6E+08 | -0.0985 | 0.0169 | 5.26E-09 | 0.000102 | 0.000105 | 0.34 | 0.004538 | 78.17354 |
| rs2638281 | G | A | 19 | 49210869 | 0.0976 | 0.0164 | 2.46E-09 | 3.07E-05 | 0.000104 | 0.77 | 0.004562 | 78.57964 |
| rs2675662 | G | A | 10 | 75599127 | -0.1184 | 0.0167 | 1.49E-12 | 6.42E-05 | 0.000103 | 0.53 | 0.006894 | 119.0325 |
| rs35741374 | T | C | 2 | 61072567 | 0.1634 | 0.0163 | 1.08E-23 | 9.83E-05 | 0.000103 | 0.34 | 0.013092 | 227.47 |
| rs3906814 | G | C | 3 | 17011474 | -0.1144 | 0.0161 | 1.30E-12 | -0.00014 | 0.000102 | 0.17 | 0.006543 | 112.9283 |
| rs4889526 | A | C | 16 | 31030344 | 0.1216 | 0.0165 | 1.62E-13 | 5.44E-07 | 0.000105 | 1 | 0.006928 | 119.629 |
| rs4978343 | T | G | 9 | 1.11E+08 | -0.0988 | 0.0164 | 1.77E-09 | 7.09E-05 | 0.000104 | 0.5 | 0.004721 | 81.34268 |
| rs559406 | T | G | 18 | 12857002 | -0.0924 | 0.0161 | 1.02E-08 | -6.39E-05 | 0.000102 | 0.53 | 0.004231 | 72.861 |
| rs6063454 | T | G | 20 | 48590791 | -0.1551 | 0.0166 | 1.08E-20 | 8.42E-05 | 0.000104 | 0.42 | 0.011671 | 202.4835 |
| rs6894840 | G | T | 5 | 1.59E+08 | 0.1152 | 0.0166 | 3.42E-12 | -3.88E-05 | 0.000105 | 0.709999 | 0.006271 | 108.2166 |
| rs771576 | C | T | 3 | 1.02E+08 | 0.1029 | 0.0169 | 1.08E-09 | -0.00012 | 0.000105 | 0.27 | 0.005001 | 86.18826 |
| rs8016947 | G | T | 14 | 35832666 | 0.1476 | 0.0162 | 8.18E-20 | 1.66E-05 | 0.000103 | 0.87 | 0.010726 | 185.9234 |
| rs9264277 | C | T | 6 | 31227901 | 0.3705 | 0.018 | 1.91E-94 | -3.50E-05 | 0.000106 | 0.74 | 0.063748 | 1167.545 |
| rs9504361 | G | A | 6 | 577820 | -0.1068 | 0.0164 | 6.44E-11 | -2.78E-05 | 0.000105 | 0.79 | 0.005638 | 97.23217 |
| rs957970 | G | A | 17 | 40519890 | 0.0929 | 0.0168 | 3.05E-08 | -8.44E-05 | 0.000107 | 0.43 | 0.003923 | 67.53892 |

Supplementary Table 14. Genetic instrumental tools used in Mendelian randomization analysis on psoriasis and prostate cancer, based on Stuart PE et al.

| SNP | Effect allele | Other allele | Chr | Pos | Exposure | | | Outcome | | | R^2^ | F |
| --- | --- | --- | --- | --- | --- | --- | --- | --- | --- | --- | --- | --- |
|  |  |  |  |  | beta | se | pval | beta | se | pval |  |  |
| rs10888503 | T | C | 1 | 1.53E+08 | 0.1866 | 0.0171 | 1.44E-27 | -0.00044 | 0.000761 | 0.56 | 0.015665 | 52.82517 |
| rs10893884 | C | T | 11 | 1.28E+08 | -0.106 | 0.016 | 3.99E-11 | 0.000312 | 0.000723 | 0.67 | 0.005615 | 18.7428 |
| rs11085744 | T | C | 19 | 10819967 | 0.1146 | 0.0166 | 5.21E-12 | -0.00062 | 0.000727 | 0.4 | 0.006476 | 21.63805 |
| rs1108618 | G | A | 10 | 81043743 | -0.1098 | 0.0165 | 3.25E-11 | 0.000235 | 0.000729 | 0.75 | 0.005913 | 19.74409 |
| rs11135059 | A | G | 5 | 1.59E+08 | -0.3078 | 0.0178 | 4.41E-67 | 0.000326 | 0.000768 | 0.67 | 0.041864 | 145.0351 |
| rs11249215 | A | G | 1 | 25297184 | 0.1403 | 0.016 | 2.14E-18 | 0.000742 | 0.000725 | 0.31 | 0.009814 | 32.89933 |
| rs115059666 | A | G | 5 | 1.5E+08 | 0.5147 | 0.0744 | 4.47E-12 | -0.00025 | 0.003452 | 0.94 | 0.005879 | 19.62881 |
| rs11581607 | A | G | 1 | 67707690 | -0.3647 | 0.036 | 4.56E-24 | 0.001108 | 0.001439 | 0.44 | 0.016704 | 56.39134 |
| rs11767350 | G | A | 7 | 37385365 | -0.0998 | 0.0164 | 1.19E-09 | 0.000197 | 0.000728 | 0.79 | 0.004869 | 16.24255 |
| rs11795343 | C | T | 9 | 32523737 | -0.1062 | 0.0164 | 1.02E-10 | -0.00082 | 0.000737 | 0.27 | 0.005418 | 18.0831 |
| rs118002009 | T | G | 11 | 64124980 | -0.102 | 0.0169 | 1.62E-09 | -0.00038 | 0.000724 | 0.6 | 0.005144 | 17.16398 |
| rs12133684 | A | G | 1 | 2.07E+08 | 0.1218 | 0.0206 | 3.26E-09 | 0.000284 | 0.000911 | 0.760001 | 0.004767 | 15.90098 |
| rs12188300 | T | A | 5 | 1.59E+08 | 0.5005 | 0.0264 | 5.39E-80 | -0.00068 | 0.001237 | 0.58 | 0.042439 | 147.1179 |
| rs12211087 | A | T | 6 | 31269946 | 1.3769 | 0.0253 | 1.00E-200 | -0.0003 | 0.001255 | 0.81 | 0.312722 | 1510.392 |
| rs1295685 | G | A | 5 | 1.32E+08 | 0.1759 | 0.0206 | 1.16E-17 | 0.000591 | 0.000937 | 0.53 | 0.009148 | 30.64528 |
| rs131656 | A | G | 22 | 21917450 | 0.114 | 0.0202 | 1.69E-08 | -0.00131 | 0.000914 | 0.15 | 0.004016 | 13.38418 |
| rs142502677 | TTG | T | 18 | 51791307 | 0.1035 | 0.0185 | 2.22E-08 | -0.0003 | 0.000823 | 0.719999 | 0.004254 | 14.18055 |
| rs1611236 | A | G | 6 | 29748690 | 0.123 | 0.0172 | 7.90E-13 | -0.00284 | 0.000772 | 0.00024 | 0.006615 | 22.10557 |
| rs1648153 | A | G | 11 | 1.1E+08 | -0.1388 | 0.0164 | 2.93E-17 | 0.000899 | 0.000729 | 0.22 | 0.009409 | 31.52969 |
| rs2021511 | T | C | 16 | 11344903 | -0.1163 | 0.0181 | 1.20E-10 | 0.00123 | 0.000804 | 0.13 | 0.005438 | 18.1488 |
| rs2066819 | T | C | 12 | 56750204 | -0.3283 | 0.0348 | 4.28E-21 | 0.001033 | 0.001444 | 0.47 | 0.013417 | 45.14414 |
| rs2111485 | G | A | 2 | 1.63E+08 | 0.1616 | 0.0166 | 1.76E-22 | -0.00119 | 0.000737 | 0.11 | 0.012452 | 41.85479 |
| rs2451258 | T | C | 6 | 1.6E+08 | -0.0985 | 0.0169 | 5.26E-09 | 4.80E-05 | 0.000747 | 0.95 | 0.004531 | 15.10851 |
| rs2638281 | G | A | 19 | 49210869 | 0.0976 | 0.0164 | 2.46E-09 | -0.00051 | 0.000737 | 0.49 | 0.00456 | 15.20723 |
| rs2675662 | G | A | 10 | 75599127 | -0.1184 | 0.0167 | 1.49E-12 | -0.00027 | 0.000727 | 0.709999 | 0.006895 | 23.04504 |
| rs2735009 | A | G | 6 | 29807853 | -0.2952 | 0.0204 | 1.63E-47 | 0.001478 | 0.000888 | 0.096 | 0.02861 | 97.76527 |
| rs28510484 | C | G | 15 | 31637569 | -0.1398 | 0.0231 | 1.37E-09 | 0.001903 | 0.000961 | 0.048 | 0.005525 | 18.44159 |
| rs28752856 | G | C | 6 | 31298421 | 1.1163 | 0.0225 | 1.00E-200 | 5.43E-05 | 0.001132 | 0.96 | 0.260215 | 1167.593 |
| rs28998802 | A | G | 17 | 26124908 | 0.2113 | 0.0222 | 1.89E-21 | -0.00016 | 0.001048 | 0.88 | 0.010793 | 36.21751 |
| rs34536443 | C | G | 19 | 10463118 | -0.6799 | 0.0504 | 2.02E-41 | 0.003406 | 0.001794 | 0.058 | 0.039638 | 137.0072 |
| rs35741374 | T | C | 2 | 61072567 | 0.1634 | 0.0163 | 1.08E-23 | -0.00061 | 0.000728 | 0.4 | 0.013093 | 44.03834 |
| rs3906814 | G | C | 3 | 17011474 | -0.1144 | 0.0161 | 1.30E-12 | 0.001266 | 0.000722 | 0.079001 | 0.006543 | 21.86122 |
| rs39841 | A | G | 5 | 96120170 | -0.1624 | 0.0176 | 2.56E-20 | -0.00084 | 0.000809 | 0.3 | 0.010435 | 35.00466 |
| rs438650 | C | T | 1 | 8290082 | 0.1244 | 0.0195 | 1.76E-10 | -1.16E-05 | 0.000864 | 0.99 | 0.005456 | 18.2116 |
| rs4712520 | C | T | 6 | 20640871 | 0.1301 | 0.0212 | 8.27E-10 | 0.001255 | 0.000932 | 0.18 | 0.005058 | 16.87567 |
| rs4889526 | A | C | 16 | 31030344 | 0.1216 | 0.0165 | 1.62E-13 | 0.000643 | 0.000746 | 0.39 | 0.00692 | 23.13011 |
| rs4978343 | T | G | 9 | 1.11E+08 | -0.0988 | 0.0164 | 1.77E-09 | -0.00067 | 0.000736 | 0.36 | 0.004719 | 15.73943 |
| rs55868394 | A | C | 17 | 73851113 | 0.1389 | 0.0246 | 1.71E-08 | 0.000248 | 0.00109 | 0.82 | 0.004272 | 14.24228 |
| rs559406 | T | G | 18 | 12857002 | -0.0924 | 0.0161 | 1.02E-08 | -6.40E-05 | 0.000724 | 0.93 | 0.004232 | 14.10717 |
| rs582757 | T | C | 6 | 1.38E+08 | -0.1846 | 0.0176 | 1.15E-25 | 0.001661 | 0.000807 | 0.04 | 0.013565 | 45.64593 |
| rs59960858 | A | C | 1 | 24519437 | -0.2086 | 0.0243 | 1.06E-17 | 0.000794 | 0.001055 | 0.450001 | 0.010116 | 33.92109 |
| rs6894840 | G | T | 5 | 1.59E+08 | 0.1152 | 0.0166 | 3.42E-12 | -0.00095 | 0.000744 | 0.2 | 0.00627 | 20.94486 |
| rs7141014 | C | T | 14 | 98667928 | -0.1123 | 0.0203 | 3.07E-08 | -8.20E-05 | 0.000892 | 0.93 | 0.004173 | 13.91005 |
| rs73986523 | C | T | 17 | 40302271 | -0.2122 | 0.038 | 2.41E-08 | 0.000191 | 0.001505 | 0.9 | 0.005178 | 17.27915 |
| rs771576 | C | T | 3 | 1.02E+08 | 0.1029 | 0.0169 | 1.08E-09 | -0.00064 | 0.000743 | 0.39 | 0.005002 | 16.68594 |
| rs8016947 | G | T | 14 | 35832666 | 0.1476 | 0.0162 | 8.18E-20 | 0.000465 | 0.000727 | 0.52 | 0.010725 | 35.98852 |
| rs9258357 | C | T | 6 | 29751129 | 0.2839 | 0.0233 | 3.48E-34 | -0.00023 | 0.000977 | 0.81 | 0.021917 | 74.38264 |
| rs9264277 | C | T | 6 | 31224667 | 0.3705 | 0.018 | 1.91E-94 | 0.000326 | 0.000749 | 0.66 | 0.06373 | 225.9463 |
| rs9277939 | A | C | 6 | 33189873 | 0.228 | 0.0253 | 2.16E-19 | 0.001011 | 0.001229 | 0.41 | 0.009075 | 30.40108 |
| rs9468618 | T | C | 6 | 29750776 | -0.1856 | 0.03 | 6.49E-10 | 0.000673 | 0.00126 | 0.59 | 0.005615 | 18.74428 |
| rs9481169 | T | G | 6 | 1.12E+08 | 0.3716 | 0.0263 | 2.47E-45 | -0.00236 | 0.001292 | 0.068 | 0.021609 | 73.31479 |
| rs9513593 | A | G | 13 | 99950260 | -0.1197 | 0.0205 | 5.33E-09 | -0.00052 | 0.000964 | 0.59 | 0.003998 | 13.32426 |
| rs957970 | G | A | 17 | 40519890 | 0.0929 | 0.0168 | 3.05E-08 | -0.00142 | 0.000756 | 0.061 | 0.003921 | 13.06584 |
| rs9591325 | C | T | 13 | 50811220 | -0.19 | 0.0324 | 4.50E-09 | 0.00184 | 0.001438 | 0.2 | 0.004575 | 15.25726 |
| rs9695923 | T | C | 9 | 1.11E+08 | -0.0955 | 0.0165 | 7.08E-09 | -0.00094 | 0.000736 | 0.2 | 0.004387 | 14.62727 |

Supplementary Table 15. Genetic instrumental tools used in Mendelian randomization analysis on psoriasis and bladder cancer, based on Stuart PE et al.

| SNP | Effect allele | Other allele | Chr | Pos | Exposure | | | Outcome | | | R^2^ | F |
| --- | --- | --- | --- | --- | --- | --- | --- | --- | --- | --- | --- | --- |
|  |  |  |  |  | beta | se | pval | beta | se | pval |  |  |
| rs10888503 | T | C | 1 | 1.53E+08 | 0.1866 | 0.0171 | 1.44E-27 | -6.12E-05 | 0.000143 | 0.67 | 0.015673 | 108.0554 |
| rs10893884 | C | T | 11 | 1.28E+08 | -0.106 | 0.016 | 3.99E-11 | 0.000231 | 0.000136 | 0.088 | 0.005614 | 38.31539 |
| rs11085744 | T | C | 19 | 10819967 | 0.1146 | 0.0166 | 5.21E-12 | -0.00011 | 0.000137 | 0.43 | 0.006476 | 44.23056 |
| rs1108618 | G | A | 10 | 81043743 | -0.1098 | 0.0165 | 3.25E-11 | -0.00021 | 0.000137 | 0.13 | 0.005911 | 40.35014 |
| rs11135059 | A | G | 5 | 1.59E+08 | -0.3078 | 0.0178 | 4.41E-67 | -8.82E-05 | 0.000144 | 0.54 | 0.041838 | 296.3183 |
| rs11249215 | A | G | 1 | 25297184 | 0.1403 | 0.016 | 2.14E-18 | 0.000141 | 0.000136 | 0.3 | 0.009815 | 67.26626 |
| rs115059666 | A | G | 5 | 1.5E+08 | 0.5147 | 0.0744 | 4.47E-12 | 0.000662 | 0.000642 | 0.3 | 0.006 | 40.96073 |
| rs11581607 | A | G | 1 | 67707690 | -0.3647 | 0.036 | 4.56E-24 | -0.00018 | 0.000271 | 0.5 | 0.016581 | 114.4201 |
| rs11767350 | G | A | 7 | 37385365 | -0.0998 | 0.0164 | 1.19E-09 | 4.27E-05 | 0.000137 | 0.75 | 0.00487 | 33.21111 |
| rs11795343 | C | T | 9 | 32523737 | -0.1062 | 0.0164 | 1.02E-10 | -0.00015 | 0.000138 | 0.28 | 0.00542 | 36.98086 |
| rs118002009 | T | G | 11 | 64124980 | -0.102 | 0.0169 | 1.62E-09 | 8.95E-05 | 0.000136 | 0.51 | 0.005145 | 35.09586 |
| rs12133684 | A | G | 1 | 2.07E+08 | 0.1218 | 0.0206 | 3.26E-09 | 0.000321 | 0.000171 | 0.061 | 0.004755 | 32.42029 |
| rs12188300 | T | A | 5 | 1.59E+08 | 0.5005 | 0.0264 | 5.39E-80 | 0.000252 | 0.000232 | 0.28 | 0.042437 | 300.7457 |
| rs12211087 | A | T | 6 | 31269946 | 1.3769 | 0.0253 | 1.00E-200 | -0.00014 | 0.000236 | 0.55 | 0.312157 | 3079.692 |
| rs1295685 | G | A | 5 | 1.32E+08 | 0.1759 | 0.0206 | 1.16E-17 | -8.67E-05 | 0.000175 | 0.62 | 0.009167 | 62.78178 |
| rs131656 | A | G | 22 | 21917450 | 0.114 | 0.0202 | 1.69E-08 | -8.52E-05 | 0.000172 | 0.62 | 0.004027 | 27.44128 |
| rs142502677 | A | T | 18 | 51791307 | 0.1035 | 0.0185 | 2.22E-08 | -4.38E-05 | 0.000155 | 0.780001 | 0.00425 | 28.96603 |
| rs1611236 | A | G | 6 | 29748690 | 0.123 | 0.0172 | 7.90E-13 | 0.000203 | 0.000145 | 0.16 | 0.006625 | 45.2603 |
| rs1648153 | A | G | 11 | 1.1E+08 | -0.1388 | 0.0164 | 2.93E-17 | -0.00013 | 0.000137 | 0.35 | 0.009405 | 64.43085 |
| rs2021511 | T | C | 16 | 11344903 | -0.1163 | 0.0181 | 1.20E-10 | -0.00013 | 0.000151 | 0.4 | 0.005426 | 37.02593 |
| rs2066819 | T | C | 12 | 56750204 | -0.3283 | 0.0348 | 4.28E-21 | -4.85E-05 | 0.000272 | 0.86 | 0.013291 | 91.41302 |
| rs2111485 | G | A | 2 | 1.63E+08 | 0.1616 | 0.0166 | 1.76E-22 | 2.32E-05 | 0.000138 | 0.87 | 0.012452 | 85.56905 |
| rs2451258 | T | C | 6 | 1.6E+08 | -0.0985 | 0.0169 | 5.26E-09 | -4.58E-05 | 0.00014 | 0.74 | 0.004535 | 30.91862 |
| rs2638281 | G | A | 19 | 49210869 | 0.0976 | 0.0164 | 2.46E-09 | -7.29E-05 | 0.000138 | 0.6 | 0.004561 | 31.09411 |
| rs2675662 | G | A | 10 | 75599127 | -0.1184 | 0.0167 | 1.49E-12 | -0.00018 | 0.000136 | 0.2 | 0.006894 | 47.11079 |
| rs2735009 | A | G | 6 | 29807853 | -0.2952 | 0.0204 | 1.63E-47 | 0.000339 | 0.000167 | 0.042 | 0.028595 | 199.7599 |
| rs28510484 | C | G | 15 | 31637569 | -0.1398 | 0.0231 | 1.37E-09 | 8.05E-05 | 0.00018 | 0.66 | 0.005516 | 37.64133 |
| rs28752856 | G | C | 6 | 31298421 | 1.1163 | 0.0225 | 1.00E-200 | -0.00017 | 0.000213 | 0.42 | 0.26009 | 2385.439 |
| rs28998802 | A | G | 17 | 26124908 | 0.2113 | 0.0222 | 1.89E-21 | -0.00013 | 0.000197 | 0.52 | 0.010791 | 74.02738 |
| rs34536443 | C | G | 19 | 10463118 | -0.6799 | 0.0504 | 2.02E-41 | 0.000463 | 0.000336 | 0.17 | 0.03984 | 281.5796 |
| rs35741374 | T | C | 2 | 61072567 | 0.1634 | 0.0163 | 1.08E-23 | 5.82E-05 | 0.000137 | 0.67 | 0.013091 | 90.01821 |
| rs3906814 | G | C | 3 | 17011474 | -0.1144 | 0.0161 | 1.30E-12 | 9.92E-05 | 0.000135 | 0.46 | 0.006543 | 44.69172 |
| rs39841 | A | G | 5 | 96120170 | -0.1624 | 0.0176 | 2.56E-20 | -0.00018 | 0.000152 | 0.23 | 0.010448 | 71.65279 |
| rs438650 | C | T | 1 | 8290082 | 0.1244 | 0.0195 | 1.76E-10 | -3.33E-05 | 0.000162 | 0.84 | 0.005462 | 37.27231 |
| rs4712520 | C | T | 6 | 20640871 | 0.1301 | 0.0212 | 8.27E-10 | -0.00024 | 0.000175 | 0.17 | 0.005065 | 34.54487 |
| rs4889526 | A | C | 16 | 31030344 | 0.1216 | 0.0165 | 1.62E-13 | 0.000175 | 0.00014 | 0.21 | 0.006924 | 47.31614 |
| rs4978343 | T | G | 9 | 1.11E+08 | -0.0988 | 0.0164 | 1.77E-09 | -0.00011 | 0.000138 | 0.44 | 0.00472 | 32.18443 |
| rs55868394 | A | C | 17 | 73851113 | 0.1389 | 0.0246 | 1.71E-08 | -0.00014 | 0.000205 | 0.5 | 0.004256 | 29.00387 |
| rs559406 | T | G | 18 | 12857002 | -0.0924 | 0.0161 | 1.02E-08 | 6.66E-05 | 0.000136 | 0.62 | 0.004232 | 28.84138 |
| rs582757 | T | C | 6 | 1.38E+08 | -0.1846 | 0.0176 | 1.15E-25 | -5.27E-05 | 0.000152 | 0.73 | 0.013563 | 93.30805 |
| rs59960858 | A | C | 1 | 24519437 | -0.2086 | 0.0243 | 1.06E-17 | -0.0006 | 0.000198 | 0.0023 | 0.010124 | 69.40244 |
| rs6894840 | G | T | 5 | 1.59E+08 | 0.1152 | 0.0166 | 3.42E-12 | -0.00015 | 0.000139 | 0.28 | 0.00627 | 42.81965 |
| rs7141014 | C | T | 14 | 98667928 | -0.1123 | 0.0203 | 3.07E-08 | 0.000134 | 0.000167 | 0.42 | 0.004173 | 28.43763 |
| rs73986523 | C | T | 17 | 40302271 | -0.2122 | 0.038 | 2.41E-08 | -9.62E-05 | 0.000283 | 0.73 | 0.005164 | 35.2253 |
| rs771576 | C | T | 3 | 1.02E+08 | 0.1029 | 0.0169 | 1.08E-09 | -0.00026 | 0.000139 | 0.059 | 0.005001 | 34.10774 |
| rs8016947 | G | T | 14 | 35832666 | 0.1476 | 0.0162 | 8.18E-20 | -0.00012 | 0.000136 | 0.38 | 0.010726 | 73.578 |
| rs9258357 | C | T | 6 | 29751129 | 0.2839 | 0.0233 | 3.48E-34 | 0.000227 | 0.000183 | 0.22 | 0.02192 | 152.089 |
| rs9264277 | C | T | 6 | 31224667 | 0.3705 | 0.018 | 1.91E-94 | -6.89E-05 | 0.000141 | 0.62 | 0.063722 | 461.8592 |
| rs9277939 | A | C | 6 | 33189873 | 0.228 | 0.0253 | 2.16E-19 | -8.34E-05 | 0.000231 | 0.719999 | 0.00905 | 61.97775 |
| rs9468618 | T | C | 6 | 29750776 | -0.1856 | 0.03 | 6.49E-10 | 0.000216 | 0.000237 | 0.36 | 0.005618 | 38.33814 |
| rs9481169 | T | G | 6 | 1.12E+08 | 0.3716 | 0.0263 | 2.47E-45 | -6.68E-05 | 0.000242 | 0.780001 | 0.021618 | 149.9428 |
| rs9513593 | A | G | 13 | 99950260 | -0.1197 | 0.0205 | 5.33E-09 | -0.00015 | 0.000181 | 0.42 | 0.004004 | 27.27973 |
| rs957970 | G | A | 17 | 40519890 | 0.0929 | 0.0168 | 3.05E-08 | 1.13E-06 | 0.000142 | 0.99 | 0.003925 | 26.7402 |
| rs9591325 | C | T | 13 | 50811220 | -0.19 | 0.0324 | 4.50E-09 | 0.000466 | 0.000269 | 0.083 | 0.004578 | 31.20946 |
| rs9695923 | T | C | 9 | 1.11E+08 | -0.0955 | 0.0165 | 7.08E-09 | -0.00012 | 0.000138 | 0.38 | 0.004389 | 29.91455 |

Supplementary Table 16. Genetic instrumental tools used in Mendelian randomization analysis on psoriasis and cervical carcinoma, based on Stuart PE et al.

| SNP | Effect allele | Other allele | Chr | Pos | Exposure | | | Outcome | | | R^2^ | F |
| --- | --- | --- | --- | --- | --- | --- | --- | --- | --- | --- | --- | --- |
|  |  |  |  |  | beta | se | pval | beta | se | pval |  |  |
| rs10888503 | T | C | 1 | 1.53E+08 | 0.1866 | 0.0171 | 1.44E-27 | -0.00018 | 0.000178 | 0.32 | 0.015683 | 58.72531 |
| rs10893884 | C | T | 11 | 1.28E+08 | -0.106 | 0.016 | 3.99E-11 | 4.04E-05 | 0.000169 | 0.81 | 0.005614 | 20.80928 |
| rs11085744 | T | C | 19 | 10819967 | 0.1146 | 0.0166 | 5.21E-12 | -6.69E-06 | 0.000171 | 0.97 | 0.006475 | 24.02104 |
| rs1108618 | G | A | 10 | 81043743 | -0.1098 | 0.0165 | 3.25E-11 | 0.000278 | 0.00017 | 0.1 | 0.00591 | 21.91156 |
| rs11135059 | A | G | 5 | 1.59E+08 | -0.3078 | 0.0178 | 4.41E-67 | 8.72E-06 | 0.00018 | 0.96 | 0.041822 | 160.8752 |
| rs11249215 | A | G | 1 | 25297184 | 0.1403 | 0.016 | 2.14E-18 | -4.33E-05 | 0.000169 | 0.8 | 0.009816 | 36.53838 |
| rs11581607 | A | G | 1 | 67707690 | -0.3647 | 0.036 | 4.56E-24 | -0.00019 | 0.000338 | 0.58 | 0.01649 | 61.79862 |
| rs11767350 | G | A | 7 | 37385365 | -0.0998 | 0.0164 | 1.19E-09 | -0.0001 | 0.00017 | 0.54 | 0.004871 | 18.04079 |
| rs11795343 | C | T | 9 | 32523737 | -0.1062 | 0.0164 | 1.02E-10 | -0.00022 | 0.000172 | 0.2 | 0.005421 | 20.08865 |
| rs118002009 | T | G | 11 | 64124980 | -0.102 | 0.0169 | 1.62E-09 | -1.93E-05 | 0.000169 | 0.91 | 0.005146 | 19.06378 |
| rs12133684 | A | G | 1 | 2.07E+08 | 0.1218 | 0.0206 | 3.26E-09 | -0.00064 | 0.000214 | 0.0029 | 0.004741 | 17.55599 |
| rs12188300 | T | A | 5 | 1.59E+08 | 0.5005 | 0.0264 | 5.39E-80 | -0.00021 | 0.00029 | 0.46 | 0.042391 | 163.1585 |
| rs12211087 | A | T | 6 | 31269946 | 1.3769 | 0.0253 | 1.00E-200 | 0.000246 | 0.000293 | 0.4 | 0.311684 | 1668.993 |
| rs1295685 | G | A | 5 | 1.32E+08 | 0.1759 | 0.0206 | 1.16E-17 | 0.000156 | 0.000218 | 0.48 | 0.009178 | 34.14201 |
| rs131656 | A | G | 22 | 21917450 | 0.114 | 0.0202 | 1.69E-08 | 2.10E-05 | 0.000214 | 0.92 | 0.004035 | 14.93217 |
| rs142502677 | A | T | 18 | 51791307 | 0.1035 | 0.0185 | 2.22E-08 | 0.000257 | 0.000193 | 0.18 | 0.004247 | 15.72113 |
| rs1611236 | A | G | 6 | 29748690 | 0.123 | 0.0172 | 7.90E-13 | 0.000124 | 0.00018 | 0.49 | 0.006627 | 24.58845 |
| rs1648153 | A | G | 11 | 1.1E+08 | -0.1388 | 0.0164 | 2.93E-17 | -0.00016 | 0.00017 | 0.36 | 0.009403 | 34.98628 |
| rs2021511 | T | C | 16 | 11344903 | -0.1163 | 0.0181 | 1.20E-10 | -0.00019 | 0.000188 | 0.32 | 0.005419 | 20.08377 |
| rs2066819 | T | C | 12 | 56750204 | -0.3283 | 0.0348 | 4.28E-21 | 0.000871 | 0.00034 | 0.01 | 0.013202 | 49.311 |
| rs2111485 | G | A | 2 | 1.63E+08 | 0.1616 | 0.0166 | 1.76E-22 | -0.00022 | 0.000172 | 0.2 | 0.012456 | 46.48912 |
| rs2451258 | T | C | 6 | 1.6E+08 | -0.0985 | 0.0169 | 5.26E-09 | -0.00011 | 0.000174 | 0.51 | 0.004539 | 16.80736 |
| rs2638281 | G | A | 19 | 49210869 | 0.0976 | 0.0164 | 2.46E-09 | 0.000389 | 0.000172 | 0.024 | 0.004562 | 16.8908 |
| rs2675662 | G | A | 10 | 75599127 | -0.1184 | 0.0167 | 1.49E-12 | 0.000142 | 0.00017 | 0.4 | 0.006894 | 25.58723 |
| rs2735009 | A | G | 6 | 29807853 | -0.2952 | 0.0204 | 1.63E-47 | 0.000202 | 0.000208 | 0.33 | 0.028595 | 108.4961 |
| rs28510484 | C | G | 15 | 31637569 | -0.1398 | 0.0231 | 1.37E-09 | 0.000421 | 0.000225 | 0.061 | 0.005516 | 20.44197 |
| rs28752856 | G | C | 6 | 31298421 | 1.1163 | 0.0225 | 1.00E-200 | 0.000122 | 0.000265 | 0.649999 | 0.260059 | 1295.395 |
| rs28998802 | A | G | 17 | 26124908 | 0.2113 | 0.0222 | 1.89E-21 | -0.0002 | 0.000245 | 0.42 | 0.010788 | 40.19599 |
| rs34536443 | C | G | 19 | 10463118 | -0.6799 | 0.0504 | 2.02E-41 | -0.0004 | 0.000416 | 0.33 | 0.040086 | 153.9158 |
| rs35741374 | T | C | 2 | 61072567 | 0.1634 | 0.0163 | 1.08E-23 | 5.60E-05 | 0.00017 | 0.74 | 0.013091 | 48.88991 |
| rs3906814 | G | C | 3 | 17011474 | -0.1144 | 0.0161 | 1.30E-12 | -0.00018 | 0.000168 | 0.28 | 0.006542 | 24.27275 |
| rs39841 | A | G | 5 | 96120170 | -0.1624 | 0.0176 | 2.56E-20 | -0.00013 | 0.000189 | 0.51 | 0.010463 | 38.97035 |
| rs438650 | C | T | 1 | 8290082 | 0.1244 | 0.0195 | 1.76E-10 | 5.66E-05 | 0.000202 | 0.780001 | 0.005468 | 20.26453 |
| rs4712520 | C | T | 6 | 20640871 | 0.1301 | 0.0212 | 8.27E-10 | 6.82E-05 | 0.000217 | 0.75 | 0.005065 | 18.76422 |
| rs4889526 | A | C | 16 | 31030344 | 0.1216 | 0.0165 | 1.62E-13 | 0.000254 | 0.000174 | 0.14 | 0.006929 | 25.71585 |
| rs4978343 | T | G | 9 | 1.11E+08 | -0.0988 | 0.0164 | 1.77E-09 | -5.23E-05 | 0.000172 | 0.760001 | 0.004721 | 17.48195 |
| rs55868394 | A | C | 17 | 73851113 | 0.1389 | 0.0246 | 1.71E-08 | -0.00023 | 0.000257 | 0.37 | 0.004242 | 15.70198 |
| rs559406 | T | G | 18 | 12857002 | -0.0924 | 0.0161 | 1.02E-08 | 0.000206 | 0.000169 | 0.22 | 0.004232 | 15.66485 |
| rs582757 | T | C | 6 | 1.38E+08 | -0.1846 | 0.0176 | 1.15E-25 | 9.40E-05 | 0.000189 | 0.62 | 0.013552 | 50.63474 |
| rs59960858 | A | C | 1 | 24519437 | -0.2086 | 0.0243 | 1.06E-17 | -0.00053 | 0.000246 | 0.032 | 0.010141 | 37.76049 |
| rs6894840 | G | T | 5 | 1.59E+08 | 0.1152 | 0.0166 | 3.42E-12 | -1.81E-05 | 0.000173 | 0.92 | 0.006271 | 23.26039 |
| rs7141014 | C | T | 14 | 98667928 | -0.1123 | 0.0203 | 3.07E-08 | 4.55E-05 | 0.000208 | 0.83 | 0.004172 | 15.44311 |
| rs73986523 | C | T | 17 | 40302271 | -0.2122 | 0.038 | 2.41E-08 | -0.00027 | 0.000353 | 0.44 | 0.005151 | 19.08505 |
| rs771576 | C | T | 3 | 1.02E+08 | 0.1029 | 0.0169 | 1.08E-09 | 0.00012 | 0.000173 | 0.49 | 0.005 | 18.52264 |
| rs8016947 | G | T | 14 | 35832666 | 0.1476 | 0.0162 | 8.18E-20 | 2.44E-06 | 0.00017 | 0.99 | 0.010726 | 39.96085 |
| rs9258357 | C | T | 6 | 29751129 | 0.2839 | 0.0233 | 3.48E-34 | -3.98E-05 | 0.000228 | 0.86 | 0.021936 | 82.66257 |
| rs9264277 | C | T | 6 | 31224667 | 0.3705 | 0.018 | 1.91E-94 | -0.00029 | 0.000175 | 0.099001 | 0.063714 | 250.8131 |
| rs9277939 | A | C | 6 | 33189873 | 0.228 | 0.0253 | 2.16E-19 | -0.00039 | 0.000287 | 0.18 | 0.009033 | 33.59602 |
| rs9468618 | T | C | 6 | 29750776 | -0.1856 | 0.03 | 6.49E-10 | -0.00028 | 0.000295 | 0.35 | 0.00562 | 20.83013 |
| rs9481169 | T | G | 6 | 1.12E+08 | 0.3716 | 0.0263 | 2.47E-45 | -0.0002 | 0.000302 | 0.51 | 0.021582 | 81.29981 |
| rs9513593 | A | G | 13 | 99950260 | -0.1197 | 0.0205 | 5.33E-09 | 0.000272 | 0.000225 | 0.23 | 0.004008 | 14.83245 |
| rs957970 | G | A | 17 | 40519890 | 0.0929 | 0.0168 | 3.05E-08 | -5.33E-05 | 0.000176 | 0.760001 | 0.003927 | 14.53114 |
| rs9591325 | C | T | 13 | 50811220 | -0.19 | 0.0324 | 4.50E-09 | 0.000218 | 0.000334 | 0.51 | 0.004584 | 16.97325 |
| rs9695923 | T | C | 9 | 1.11E+08 | -0.0955 | 0.0165 | 7.08E-09 | -1.59E-05 | 0.000172 | 0.93 | 0.00439 | 16.25039 |

Supplementary Table 17. Genetic instrumental tools used in Mendelian randomization analysis on psoriasis and ovarian cancer, based on Stuart PE et al.

| SNP | Effect allele | Other allele | Chr | Pos | Exposure | | | Outcome | | | R^2^ | F |
| --- | --- | --- | --- | --- | --- | --- | --- | --- | --- | --- | --- | --- |
|  |  |  |  |  | beta | se | pval | beta | se | pval |  |  |
| rs10888503 | T | C | 1 | 1.53E+08 | 0.1866 | 0.0171 | 1.44E-27 | -0.00022 | 0.00026 | 0.39 | 0.015683 | 57.84786 |
| rs10893884 | C | T | 11 | 1.28E+08 | -0.106 | 0.016 | 3.99E-11 | -0.00032 | 0.000247 | 0.19 | 0.005614 | 20.49826 |
| rs11085744 | T | C | 19 | 10819967 | 0.1146 | 0.0166 | 5.21E-12 | -0.00017 | 0.00025 | 0.49 | 0.006475 | 23.66226 |
| rs1108618 | G | A | 10 | 81043743 | -0.1098 | 0.0165 | 3.25E-11 | 0.000156 | 0.000249 | 0.53 | 0.00591 | 21.58348 |
| rs11135059 | A | G | 5 | 1.59E+08 | -0.3078 | 0.0178 | 4.41E-67 | -6.91E-05 | 0.000263 | 0.79 | 0.041821 | 158.4649 |
| rs11249215 | A | G | 1 | 25297184 | 0.1403 | 0.016 | 2.14E-18 | -0.00011 | 0.000248 | 0.67 | 0.009816 | 35.99181 |
| rs115059666 | A | G | 5 | 1.5E+08 | 0.5147 | 0.0744 | 4.47E-12 | -0.0013 | 0.001163 | 0.26 | 0.00609 | 22.2471 |
| rs11581607 | A | G | 1 | 67707690 | -0.3647 | 0.036 | 4.56E-24 | 0.00033 | 0.000494 | 0.5 | 0.016498 | 60.90209 |
| rs11767350 | G | A | 7 | 37385365 | -0.0998 | 0.0164 | 1.19E-09 | -0.00023 | 0.000249 | 0.36 | 0.004871 | 17.77066 |
| rs11795343 | C | T | 9 | 32523737 | -0.1062 | 0.0164 | 1.02E-10 | 0.000293 | 0.000252 | 0.25 | 0.005421 | 19.79025 |
| rs118002009 | T | G | 11 | 64124980 | -0.102 | 0.0169 | 1.62E-09 | 6.70E-05 | 0.000248 | 0.79 | 0.005146 | 18.77885 |
| rs12133684 | A | G | 1 | 2.07E+08 | 0.1218 | 0.0206 | 3.26E-09 | 0.000416 | 0.000312 | 0.18 | 0.004744 | 17.30414 |
| rs12188300 | T | A | 5 | 1.59E+08 | 0.5005 | 0.0264 | 5.39E-80 | -0.00055 | 0.000424 | 0.2 | 0.042379 | 160.6732 |
| rs12211087 | A | T | 6 | 31269946 | 1.3769 | 0.0253 | 1.00E-200 | -0.00048 | 0.00043 | 0.26 | 0.311495 | 1642.581 |
| rs1295685 | G | A | 5 | 1.32E+08 | 0.1759 | 0.0206 | 1.16E-17 | -4.88E-05 | 0.000319 | 0.88 | 0.009179 | 33.63592 |
| rs131656 | A | G | 22 | 21917450 | 0.114 | 0.0202 | 1.69E-08 | -0.00011 | 0.000313 | 0.719999 | 0.004035 | 14.70768 |
| rs142502677 | A | T | 18 | 51791307 | 0.1035 | 0.0185 | 2.22E-08 | -0.00025 | 0.000282 | 0.38 | 0.004246 | 15.48254 |
| rs1611236 | A | G | 6 | 29748690 | 0.123 | 0.0172 | 7.90E-13 | 0.000105 | 0.000263 | 0.69 | 0.006627 | 24.22052 |
| rs1648153 | A | G | 11 | 1.1E+08 | -0.1388 | 0.0164 | 2.93E-17 | 4.13E-05 | 0.000249 | 0.87 | 0.009403 | 34.46413 |
| rs2021511 | T | C | 16 | 11344903 | -0.1163 | 0.0181 | 1.20E-10 | -0.00022 | 0.000275 | 0.42 | 0.005419 | 19.78305 |
| rs2066819 | T | C | 12 | 56750204 | -0.3283 | 0.0348 | 4.28E-21 | 0.000139 | 0.000498 | 0.780001 | 0.013194 | 48.5421 |
| rs2111485 | G | A | 2 | 1.63E+08 | 0.1616 | 0.0166 | 1.76E-22 | 0.000388 | 0.000252 | 0.12 | 0.012454 | 45.78771 |
| rs2451258 | T | C | 6 | 1.6E+08 | -0.0985 | 0.0169 | 5.26E-09 | 7.57E-05 | 0.000255 | 0.77 | 0.004539 | 16.55517 |
| rs2638281 | G | A | 19 | 49210869 | 0.0976 | 0.0164 | 2.46E-09 | 5.99E-05 | 0.000252 | 0.81 | 0.004562 | 16.63933 |
| rs2675662 | G | A | 10 | 75599127 | -0.1184 | 0.0167 | 1.49E-12 | -0.0003 | 0.000249 | 0.23 | 0.006894 | 25.20313 |
| rs2735009 | A | G | 6 | 29807853 | -0.2952 | 0.0204 | 1.63E-47 | 0.000402 | 0.000304 | 0.19 | 0.028598 | 106.8864 |
| rs28510484 | C | G | 15 | 31637569 | -0.1398 | 0.0231 | 1.37E-09 | 0.000395 | 0.000329 | 0.23 | 0.005516 | 20.13596 |
| rs28752856 | G | C | 6 | 31298421 | 1.1163 | 0.0225 | 1.00E-200 | -0.00073 | 0.000388 | 0.059 | 0.259894 | 1274.924 |
| rs28998802 | A | G | 17 | 26124908 | 0.2113 | 0.0222 | 1.89E-21 | 0.000347 | 0.000359 | 0.33 | 0.010792 | 39.61007 |
| rs34536443 | C | G | 19 | 10463118 | -0.6799 | 0.0504 | 2.02E-41 | 0.00033 | 0.000609 | 0.59 | 0.040111 | 151.7132 |
| rs35741374 | T | C | 2 | 61072567 | 0.1634 | 0.0163 | 1.08E-23 | -0.00019 | 0.000249 | 0.450001 | 0.013091 | 48.16042 |
| rs3906814 | G | C | 3 | 17011474 | -0.1144 | 0.0161 | 1.30E-12 | -0.00043 | 0.000246 | 0.077999 | 0.006542 | 23.90982 |
| rs39841 | A | G | 5 | 96120170 | -0.1624 | 0.0176 | 2.56E-20 | 0.000228 | 0.000276 | 0.41 | 0.010461 | 38.3813 |
| rs438650 | C | T | 1 | 8290082 | 0.1244 | 0.0195 | 1.76E-10 | -0.0002 | 0.000296 | 0.5 | 0.005467 | 19.9587 |
| rs4712520 | C | T | 6 | 20640871 | 0.1301 | 0.0212 | 8.27E-10 | -0.00023 | 0.000318 | 0.47 | 0.005066 | 18.48705 |
| rs4889526 | A | C | 16 | 31030344 | 0.1216 | 0.0165 | 1.62E-13 | 0.00026 | 0.000255 | 0.31 | 0.006929 | 25.33128 |
| rs4978343 | T | G | 9 | 1.11E+08 | -0.0988 | 0.0164 | 1.77E-09 | -9.56E-05 | 0.000252 | 0.7 | 0.004721 | 17.22037 |
| rs55868394 | A | C | 17 | 73851113 | 0.1389 | 0.0246 | 1.71E-08 | -0.00028 | 0.000376 | 0.450001 | 0.004242 | 15.46651 |
| rs559406 | T | G | 18 | 12857002 | -0.0924 | 0.0161 | 1.02E-08 | -0.00016 | 0.000247 | 0.51 | 0.004232 | 15.43109 |
| rs582757 | T | C | 6 | 1.38E+08 | -0.1846 | 0.0176 | 1.15E-25 | -0.00025 | 0.000277 | 0.37 | 0.013554 | 49.88529 |
| rs59960858 | A | C | 1 | 24519437 | -0.2086 | 0.0243 | 1.06E-17 | -0.00037 | 0.00036 | 0.31 | 0.010142 | 37.20024 |
| rs6894840 | G | T | 5 | 1.59E+08 | 0.1152 | 0.0166 | 3.42E-12 | 4.27E-06 | 0.000254 | 0.99 | 0.006271 | 22.91242 |
| rs7141014 | C | T | 14 | 98667928 | -0.1123 | 0.0203 | 3.07E-08 | -0.00022 | 0.000304 | 0.48 | 0.004172 | 15.20978 |
| rs73986523 | C | T | 17 | 40302271 | -0.2122 | 0.038 | 2.41E-08 | -0.0007 | 0.000516 | 0.17 | 0.005149 | 18.79265 |
| rs771576 | C | T | 3 | 1.02E+08 | 0.1029 | 0.0169 | 1.08E-09 | -0.00027 | 0.000254 | 0.29 | 0.005001 | 18.24732 |
| rs8016947 | G | T | 14 | 35832666 | 0.1476 | 0.0162 | 8.18E-20 | 0.000323 | 0.000248 | 0.19 | 0.010725 | 39.36161 |
| rs9258357 | C | T | 6 | 29751129 | 0.2839 | 0.0233 | 3.48E-34 | 0.000114 | 0.000334 | 0.73 | 0.021933 | 81.41804 |
| rs9264277 | C | T | 6 | 31224667 | 0.3705 | 0.018 | 1.91E-94 | -1.29E-05 | 0.000256 | 0.96 | 0.063709 | 247.0428 |
| rs9277939 | A | C | 6 | 33189873 | 0.228 | 0.0253 | 2.16E-19 | 0.000164 | 0.00042 | 0.7 | 0.009037 | 33.10816 |
| rs9468618 | T | C | 6 | 29750776 | -0.1856 | 0.03 | 6.49E-10 | 0.000358 | 0.000432 | 0.41 | 0.005623 | 20.52919 |
| rs9481169 | T | G | 6 | 1.12E+08 | 0.3716 | 0.0263 | 2.47E-45 | -0.00019 | 0.000442 | 0.68 | 0.021582 | 80.08479 |
| rs9513593 | A | G | 13 | 99950260 | -0.1197 | 0.0205 | 5.33E-09 | 0.000217 | 0.00033 | 0.51 | 0.004008 | 14.61111 |
| rs957970 | G | A | 17 | 40519890 | 0.0929 | 0.0168 | 3.05E-08 | -0.00016 | 0.000258 | 0.55 | 0.003927 | 14.31337 |
| rs9591325 | C | T | 13 | 50811220 | -0.19 | 0.0324 | 4.50E-09 | 0.000117 | 0.000489 | 0.81 | 0.004584 | 16.71804 |
| rs9695923 | T | C | 9 | 1.11E+08 | -0.0955 | 0.0165 | 7.08E-09 | -8.91E-05 | 0.000252 | 0.719999 | 0.00439 | 16.00714 |

Supplementary Table 18. Genetic instrumental tools used in Mendelian randomization analysis on psoriasis and brain cancer, based on FinnGen.

| SNP | Effect allele | Other allele | Chr | Pos | Exposure | | | Outcome | | | R^2^ | F |
| --- | --- | --- | --- | --- | --- | --- | --- | --- | --- | --- | --- | --- |
|  |  |  |  |  | beta | se | pval | beta | se | pval |  |  |
| rs10829130 | A | G | 10 | 27174346 | 0.1965 | 0.0359 | 4.24E-08 | -6.00E-05 | 0.000142 | 0.67 | 0.00756 | 218.3446 |
| rs12188300 | T | A | 5 | 1.59E+08 | 0.4331 | 0.0495 | 2.24E-18 | 9.11E-05 | 0.00016 | 0.57 | 0.020821 | 609.4764 |
| rs12713428 | C | A | 2 | 61118113 | 0.1694 | 0.0261 | 8.11E-11 | -0.00018 | 0.000111 | 0.1 | 0.010724 | 310.6971 |
| rs13210419 | A | G | 6 | 31266977 | 1.1157 | 0.0511 | 1.10E-105 | -4.27E-05 | 0.000178 | 0.81 | 0.137296 | 4561.468 |
| rs1611309 | T | C | 6 | 29902063 | 0.241 | 0.0267 | 1.60E-19 | 1.75E-05 | 0.000109 | 0.87 | 0.027179 | 800.764 |
| rs17728338 | A | G | 5 | 1.5E+08 | 0.3092 | 0.0439 | 1.76E-12 | 0.000128 | 0.000214 | 0.55 | 0.01299 | 377.2193 |
| rs2021511 | T | C | 16 | 11344903 | -0.1387 | 0.0254 | 4.75E-08 | 0.000109 | 0.000104 | 0.3 | 0.007519 | 217.1517 |
| rs28752856 | G | C | 6 | 31298421 | 0.833 | 0.0393 | 5.90E-100 | -1.98E-05 | 0.000147 | 0.89 | 0.136512 | 4531.323 |
| rs28998802 | A | G | 17 | 26124908 | 0.1672 | 0.0289 | 7.41E-09 | -1.42E-05 | 0.000136 | 0.92 | 0.008434 | 243.7813 |
| rs4713605 | A | T | 6 | 32985992 | 0.1526 | 0.0241 | 2.35E-10 | 7.06E-05 | 9.69E-05 | 0.47 | 0.01033 | 299.1837 |
| rs60600003 | G | T | 7 | 37382465 | 0.2128 | 0.0372 | 1.03E-08 | 0.000373 | 0.000156 | 0.017 | 0.008274 | 239.1293 |
| rs674451 | C | T | 6 | 1.38E+08 | 0.1307 | 0.0235 | 2.82E-08 | 7.43E-05 | 9.94E-05 | 0.450001 | 0.007699 | 222.3853 |
| rs9481169 | T | G | 6 | 1.12E+08 | 0.2515 | 0.0422 | 2.47E-09 | -0.00018 | 0.000167 | 0.27 | 0.009116 | 263.6834 |

Supplementary Table 19. Genetic instrumental tools used in Mendelian randomization analysis on psoriasis and head and neck cancer, based on FinnGen.

| SNP | Effect allele | Other allele | Chr | Pos | Exposure | | | Outcome | | | R^2^ | F |
| --- | --- | --- | --- | --- | --- | --- | --- | --- | --- | --- | --- | --- |
|  |  |  |  |  | beta | se | pval | beta | se | pval |  |  |
| rs10829130 | A | G | 10 | 27174346 | 0.1965 | 0.0359 | 4.24E-08 | 0.000269 | 0.000191 | 4.24E-08 | 0.00756 | 218.6376 |
| rs12188300 | T | A | 5 | 158829527 | 0.4331 | 0.0495 | 2.24E-18 | 0.000156 | 0.000216 | 2.24E-18 | 0.020821 | 610.2943 |
| rs12713428 | C | A | 2 | 61118113 | 0.1694 | 0.0261 | 8.11E-11 | -0.00013 | 0.00015 | 8.11E-11 | 0.010724 | 311.114 |
| rs13210419 | A | G | 6 | 31266977 | 1.1157 | 0.0511 | 1.10E-105 | 0.000285 | 0.00024 | 1.10E-105 | 0.137296 | 4567.589 |
| rs1611309 | T | C | 6 | 29902063 | 0.241 | 0.0267 | 1.60E-19 | 0.000289 | 0.000148 | 1.60E-19 | 0.027179 | 801.8385 |
| rs17728338 | A | G | 5 | 150478318 | 0.3092 | 0.0439 | 1.76E-12 | -0.00038 | 0.000288 | 1.76E-12 | 0.01299 | 377.7255 |
| rs2021511 | T | C | 16 | 11344903 | -0.1387 | 0.0254 | 4.75E-08 | 9.26E-05 | 0.00014 | 4.75E-08 | 0.007519 | 217.4431 |
| rs28752856 | G | C | 6 | 31298421 | 0.833 | 0.0393 | 5.90E-100 | 0.000266 | 0.000198 | 5.90E-100 | 0.136512 | 4537.403 |
| rs28998802 | A | G | 17 | 26124908 | 0.1672 | 0.0289 | 7.41E-09 | 0.000295 | 0.000183 | 7.41E-09 | 0.008434 | 244.1084 |
| rs4713605 | A | T | 6 | 32985992 | 0.1526 | 0.0241 | 2.35E-10 | 0.000147 | 0.000131 | 2.35E-10 | 0.01033 | 299.5852 |
| rs60600003 | G | T | 7 | 37382465 | 0.2128 | 0.0372 | 1.03E-08 | 0.000255 | 0.000211 | 1.03E-08 | 0.008274 | 239.4501 |
| rs674451 | C | T | 6 | 138216788 | 0.1307 | 0.0235 | 2.82E-08 | 6.76E-05 | 0.000134 | 2.82E-08 | 0.007699 | 222.6837 |
| rs9481169 | T | G | 6 | 111929862 | 0.2515 | 0.0422 | 2.47E-09 | 0.000212 | 0.000225 | 2.47E-09 | 0.009116 | 264.0373 |

Supplementary Table 20. Genetic instrumental tools used in Mendelian randomization analysis on psoriasis and skin cancer, based on FinnGen.

| SNP | Effect allele | Other allele | Chr | Pos | Exposure | | | Outcome | | | R^2^ | F |
| --- | --- | --- | --- | --- | --- | --- | --- | --- | --- | --- | --- | --- |
|  |  |  |  |  | beta | se | pval | beta | se | pval |  |  |
| rs10829130 | A | G | 10 | 27174346 | 0.1965 | 0.0359 | 4.24E-08 | -0.00022 | 0.000358 | 0.54 | 0.00756 | 320.6418 |
| rs12188300 | T | A | 5 | 1.59E+08 | 0.4331 | 0.0495 | 2.24E-18 | -0.00026 | 0.000405 | 0.53 | 0.020821 | 895.0236 |
| rs12713428 | C | A | 2 | 61118113 | 0.1694 | 0.0261 | 8.11E-11 | 5.97E-05 | 0.000281 | 0.83 | 0.010724 | 456.2625 |
| rs13210419 | A | G | 6 | 31266977 | 1.1157 | 0.0511 | 1.10E-105 | 0.000132 | 0.000449 | 0.77 | 0.137296 | 6698.572 |
| rs2021511 | T | C | 16 | 11344903 | -0.1387 | 0.0254 | 4.75E-08 | 0.000231 | 0.000263 | 0.38 | 0.007519 | 318.8899 |
| rs28752856 | G | C | 6 | 31298547 | 0.833 | 0.0393 | 5.90E-100 | -0.00055 | 0.00042 | 0.19 | 0.136512 | 6654.303 |
| rs28998802 | A | G | 17 | 26124908 | 0.1672 | 0.0289 | 7.41E-09 | 0.000226 | 0.000344 | 0.51 | 0.008434 | 357.9958 |
| rs4713605 | A | T | 6 | 32985992 | 0.1526 | 0.0241 | 2.35E-10 | -0.00019 | 0.000245 | 0.43 | 0.01033 | 439.355 |
| rs60600003 | G | T | 7 | 37382465 | 0.2128 | 0.0372 | 1.03E-08 | -1.53E-05 | 0.000395 | 0.97 | 0.008274 | 351.1643 |
| rs674451 | C | T | 6 | 1.38E+08 | 0.1307 | 0.0235 | 2.82E-08 | -0.00012 | 0.000251 | 0.62 | 0.007699 | 326.5756 |
| rs9481169 | T | G | 6 | 1.12E+08 | 0.2515 | 0.0422 | 2.47E-09 | -0.00011 | 0.000423 | 0.79 | 0.009116 | 387.2224 |

Supplementary Table 21. Genetic instrumental tools used in Mendelian randomization analysis on psoriasis and melanoma, based on FinnGen.

| SNP | Effect allele | Other allele | Chr | Pos | Exposure | | | Outcome | | | R^2^ | F |
| --- | --- | --- | --- | --- | --- | --- | --- | --- | --- | --- | --- | --- |
|  |  |  |  |  | beta | se | pval | beta | se | pval |  |  |
| rs4713605 | A | T | 6 | 32985992 | 0.1526 | 0.0241 | 2.35E-10 | -0.00013 | 0.000103 | 0.21 | 0.01033 | 2416.5 |
| rs674451 | C | T | 6 | 1.38E+08 | 0.1307 | 0.0235 | 2.82E-08 | -0.00011 | 0.000104 | 0.31 | 0.007699 | 1796.201 |

Supplementary Table 22. Genetic instrumental tools used in Mendelian randomization analysis on psoriasis and multiple myeloma, based on FinnGen.

| SNP | Effect allele | Other allele | Chr | Pos | Exposure | | | Outcome | | | R^2^ | F |
| --- | --- | --- | --- | --- | --- | --- | --- | --- | --- | --- | --- | --- |
|  |  |  |  |  | beta | se | pval | beta | se | pval |  |  |
| rs10829130 | A | G | 10 | 27174346 | 0.1965 | 0.0359 | 4.24E-08 | -9.84E-05 | 0.000141 | 0.49 | 0.00756 | 218.3417 |
| rs12188300 | T | A | 5 | 1.59E+08 | 0.4331 | 0.0495 | 2.24E-18 | -3.97E-05 | 0.00016 | 0.8 | 0.020821 | 609.4682 |
| rs12713428 | C | A | 2 | 61118113 | 0.1694 | 0.0261 | 8.11E-11 | 1.44E-05 | 0.000111 | 0.9 | 0.010724 | 310.6929 |
| rs13210419 | A | G | 6 | 31266977 | 1.1157 | 0.0511 | 1.10E-105 | -1.65E-05 | 0.000177 | 0.93 | 0.137296 | 4561.407 |
| rs1611309 | T | C | 6 | 29902063 | 0.241 | 0.0267 | 1.60E-19 | 2.51E-05 | 0.000109 | 0.82 | 0.027179 | 800.7533 |
| rs17728338 | A | G | 5 | 1.5E+08 | 0.3092 | 0.0439 | 1.76E-12 | 0.00021 | 0.000213 | 0.33 | 0.01299 | 377.2142 |
| rs2021511 | T | C | 16 | 11344903 | -0.1387 | 0.0254 | 4.75E-08 | 1.63E-05 | 0.000104 | 0.88 | 0.007519 | 217.1488 |
| rs28752856 | G | C | 6 | 31298421 | 0.833 | 0.0393 | 5.90E-100 | 6.48E-05 | 0.000146 | 0.66 | 0.136512 | 4531.262 |
| rs28998802 | A | G | 17 | 26124908 | 0.1672 | 0.0289 | 7.41E-09 | 4.79E-05 | 0.000136 | 0.719999 | 0.008434 | 243.778 |
| rs4713605 | A | T | 6 | 32985992 | 0.1526 | 0.0241 | 2.35E-10 | 1.69E-05 | 9.65E-05 | 0.86 | 0.01033 | 299.1797 |
| rs60600003 | G | T | 7 | 37382465 | 0.2128 | 0.0372 | 1.03E-08 | -0.0001 | 0.000156 | 0.51 | 0.008274 | 239.1261 |
| rs674451 | C | T | 6 | 1.38E+08 | 0.1307 | 0.0235 | 2.82E-08 | 5.76E-05 | 9.91E-05 | 0.56 | 0.007699 | 222.3823 |
| rs9481169 | T | G | 6 | 1.12E+08 | 0.2515 | 0.0422 | 2.47E-09 | 0.000243 | 0.000167 | 0.15 | 0.009116 | 263.6799 |

Supplementary Table 23. Genetic instrumental tools used in Mendelian randomization analysis on psoriasis and breast cancer, based on FinnGen.

| SNP | Effect allele | Other allele | Chr | Pos | Exposure | | | Outcome | | | R^2^ | F |
| --- | --- | --- | --- | --- | --- | --- | --- | --- | --- | --- | --- | --- |
|  |  |  |  |  | beta | se | pval | beta | se | pval |  |  |
| rs10829130 | A | G | 10 | 27174346 | 0.1965 | 0.0359 | 4.24E-08 | 7.00E-04 | 0.0095 | 0.9417 | 0.00756 | 124.5718 |
| rs12188300 | T | A | 5 | 1.59E+08 | 0.4331 | 0.0495 | 2.24E-18 | 0.0056 | 0.0121 | 0.643101 | 0.020821 | 347.7236 |
| rs12713428 | C | A | 2 | 61118113 | 0.1694 | 0.0261 | 8.11E-11 | 6.00E-04 | 0.0072 | 0.9326 | 0.010724 | 177.2615 |
| rs13210419 | A | G | 6 | 31266977 | 1.1157 | 0.0511 | 1.10E-105 | 0.0219 | 0.0119 | 0.06572 | 0.137296 | 2602.447 |
| rs138009430 | A | C | 16 | 27302897 | 0.2538 | 0.0423 | 1.94E-09 | 0.0302 | 0.0471 | 0.5221 | 0.009318 | 153.8082 |
| rs17728338 | A | G | 5 | 1.5E+08 | 0.3092 | 0.0439 | 1.76E-12 | 0.0031 | 0.0143 | 0.8278 | 0.01299 | 215.2143 |
| rs181316459 | C | G | 7 | 5473610 | 0.3544 | 0.0553 | 1.50E-10 | 0.0049 | 0.0253 | 0.8475 | 0.011333 | 187.4537 |
| rs2021511 | T | C | 16 | 11344903 | -0.1387 | 0.0254 | 4.75E-08 | -0.0107 | 0.0072 | 0.1392 | 0.007519 | 123.8912 |
| rs28752856 | G | C | 6 | 31298421 | 0.833 | 0.0393 | 5.90E-100 | 0.0064 | 0.0097 | 0.5072 | 0.136512 | 2585.248 |
| rs28998802 | A | G | 17 | 26124908 | 0.1672 | 0.0289 | 7.41E-09 | -0.0058 | 0.0093 | 0.5317 | 0.008434 | 139.0841 |
| rs4713605 | A | T | 6 | 32985992 | 0.1526 | 0.0241 | 2.35E-10 | -0.0027 | 0.0065 | 0.677901 | 0.01033 | 170.6928 |
| rs60600003 | G | T | 7 | 37382465 | 0.2128 | 0.0372 | 1.03E-08 | 0.0014 | 0.011 | 0.8986 | 0.008274 | 136.43 |
| rs674451 | C | T | 6 | 1.38E+08 | 0.1307 | 0.0235 | 2.82E-08 | 0.0052 | 0.007 | 0.4517 | 0.007699 | 126.8771 |
| rs9481169 | T | G | 6 | 1.12E+08 | 0.2515 | 0.0422 | 2.47E-09 | -0.0158 | 0.0113 | 0.1625 | 0.009116 | 150.4389 |

Supplementary Table 24. Genetic instrumental tools used in Mendelian randomization analysis on psoriasis and lung cancer, based on FinnGen.

| SNP | Effect allele | Other allele | Chr | Pos | Exposure | | | Outcome | | | R^2^ | F |
| --- | --- | --- | --- | --- | --- | --- | --- | --- | --- | --- | --- | --- |
|  |  |  |  |  | beta | se | pval | beta | se | pval |  |  |
| rs10829130 | A | G | 10 | 27174346 | 0.1965 | 0.0359 | 4.24E-08 | -0.00039 | 0.000295 | 0.19 | 0.00756 | 219.5547 |
| rs12188300 | T | A | 5 | 1.59E+08 | 0.4331 | 0.0495 | 2.24E-18 | -0.00052 | 0.000334 | 0.12 | 0.020821 | 612.8541 |
| rs12713428 | C | A | 2 | 61118113 | 0.1694 | 0.0261 | 8.11E-11 | -0.00043 | 0.000232 | 0.061 | 0.010724 | 312.419 |
| rs13210419 | A | G | 6 | 31266977 | 1.1157 | 0.0511 | 1.10E-105 | 0.001112 | 0.00037 | 0.0027 | 0.137296 | 4586.748 |
| rs1611309 | T | C | 6 | 29902063 | 0.241 | 0.0267 | 1.60E-19 | -0.0003 | 0.000228 | 0.19 | 0.027179 | 805.2018 |
| rs17728338 | A | G | 5 | 1.5E+08 | 0.3092 | 0.0439 | 1.76E-12 | 0.000129 | 0.000445 | 0.77 | 0.01299 | 379.3099 |
| rs2021511 | T | C | 16 | 11344903 | -0.1387 | 0.0254 | 4.75E-08 | -0.0004 | 0.000217 | 0.069 | 0.007519 | 218.3551 |
| rs28752856 | G | C | 6 | 31298421 | 0.833 | 0.0393 | 5.90E-100 | -0.00058 | 0.000306 | 0.058 | 0.136512 | 4556.435 |
| rs28998802 | A | G | 17 | 26124908 | 0.1672 | 0.0289 | 7.41E-09 | -0.00018 | 0.000283 | 0.52 | 0.008434 | 245.1323 |
| rs4713605 | A | T | 6 | 32985992 | 0.1526 | 0.0241 | 2.35E-10 | -0.00011 | 0.000202 | 0.6 | 0.01033 | 300.8418 |
| rs60600003 | G | T | 7 | 37382465 | 0.2128 | 0.0372 | 1.03E-08 | -0.00021 | 0.000326 | 0.52 | 0.008274 | 240.4545 |
| rs674451 | C | T | 6 | 1.38E+08 | 0.1307 | 0.0235 | 2.82E-08 | -4.85E-05 | 0.000207 | 0.81 | 0.007699 | 223.6178 |
| rs9481169 | T | G | 6 | 1.12E+08 | 0.2515 | 0.0422 | 2.47E-09 | -0.00049 | 0.000348 | 0.16 | 0.009116 | 265.1448 |

Supplementary Table 25. Genetic instrumental tools used in Mendelian randomization analysis on psoriasis and esophageal cancer, based on FinnGen.

| SNP | Effect allele | Other allele | Chr | Pos | Exposure | | | Outcome | | | R^2^ | F |
| --- | --- | --- | --- | --- | --- | --- | --- | --- | --- | --- | --- | --- |
|  |  |  |  |  | beta | se | pval | beta | se | pval |  |  |
| rs10829130 | A | G | 10 | 27174346 | 0.1965 | 0.0359 | 4.24E-08 | -2.04E-05 | 0.000156 | 0.9 | 0.00756 | 215.1329 |
| rs12188300 | T | A | 5 | 1.59E+08 | 0.4331 | 0.0495 | 2.24E-18 | 1.34E-05 | 0.000177 | 0.94 | 0.020821 | 584.5705 |
| rs12713428 | C | A | 2 | 61118113 | 0.1694 | 0.0261 | 8.11E-11 | 7.20E-05 | 0.000123 | 0.56 | 0.010724 | 304.1785 |
| rs13210419 | A | G | 6 | 31266977 | 1.1157 | 0.0511 | 1.10E-105 | -6.47E-05 | 0.000197 | 0.74 | 0.137296 | 3396.131 |
| rs1611309 | T | C | 6 | 29902063 | 0.241 | 0.0267 | 1.60E-19 | -3.12E-05 | 0.000121 | 0.8 | 0.027179 | 758.1006 |
| rs17728338 | A | G | 5 | 1.5E+08 | 0.3092 | 0.0439 | 1.76E-12 | -7.94E-05 | 0.000236 | 0.74 | 0.01299 | 367.615 |
| rs2021511 | T | C | 16 | 11344903 | -0.1387 | 0.0254 | 4.75E-08 | 3.87E-05 | 0.000115 | 0.74 | 0.007519 | 213.9752 |
| rs28752856 | G | C | 6 | 31298421 | 0.833 | 0.0393 | 5.90E-100 | 0.000122 | 0.000162 | 0.450001 | 0.136512 | 3379.818 |
| rs28998802 | A | G | 17 | 26124908 | 0.1672 | 0.0289 | 7.41E-09 | 0.000151 | 0.00015 | 0.31 | 0.008434 | 239.7729 |
| rs4713605 | A | T | 6 | 32985992 | 0.1526 | 0.0241 | 2.35E-10 | 2.79E-05 | 0.000107 | 0.79 | 0.01033 | 293.1397 |
| rs60600003 | G | T | 7 | 37382465 | 0.2128 | 0.0372 | 1.03E-08 | 5.29E-05 | 0.000173 | 0.760001 | 0.008274 | 235.2731 |
| rs674451 | C | T | 6 | 1.38E+08 | 0.1307 | 0.0235 | 2.82E-08 | 0.000173 | 0.00011 | 0.11 | 0.007699 | 219.0529 |
| rs9481169 | T | G | 6 | 1.12E+08 | 0.2515 | 0.0422 | 2.47E-09 | -0.00023 | 0.000185 | 0.21 | 0.009116 | 258.9911 |

Supplementary Table 26. Genetic instrumental tools used in Mendelian randomization analysis on psoriasis and liver cancer, based on FinnGen.

| SNP | Effect allele | Other allele | Chr | Pos | Exposure | | | Outcome | | | R^2^ | F |
| --- | --- | --- | --- | --- | --- | --- | --- | --- | --- | --- | --- | --- |
|  |  |  |  |  | beta | se | pval | beta | se | pval |  |  |
| rs10829130 | A | G | 10 | 27174346 | 0.1965 | 0.0359 | 4.24E-08 | -4.14E-05 | 7.47E-05 | 0.58 | 0.00756 | 236.2626 |
| rs12188300 | T | A | 5 | 1.59E+08 | 0.4331 | 0.0495 | 2.24E-18 | -0.0001 | 8.46E-05 | 0.23 | 0.020821 | 659.4917 |
| rs12713428 | C | A | 2 | 61118113 | 0.1694 | 0.0261 | 8.11E-11 | -0.00012 | 5.87E-05 | 0.038 | 0.010724 | 336.1938 |
| rs13210419 | A | G | 6 | 31266977 | 1.1157 | 0.0511 | 1.10E-105 | 6.15E-05 | 9.39E-05 | 0.51 | 0.137296 | 4935.795 |
| rs1611309 | T | C | 6 | 29902063 | 0.241 | 0.0267 | 1.60E-19 | -2.69E-06 | 5.77E-05 | 0.96 | 0.027179 | 866.4769 |
| rs2021511 | T | C | 16 | 11344903 | -0.1387 | 0.0254 | 4.75E-08 | -2.79E-05 | 5.49E-05 | 0.61 | 0.007519 | 234.9718 |
| rs28752856 | G | C | 6 | 31298421 | 0.833 | 0.0393 | 5.90E-100 | 0.000184 | 7.74E-05 | 0.018 | 0.136512 | 4903.176 |
| rs28998802 | A | G | 17 | 26124908 | 0.1672 | 0.0289 | 7.41E-09 | -2.83E-05 | 7.17E-05 | 0.69 | 0.008434 | 263.7866 |
| rs4713605 | A | T | 6 | 32985992 | 0.1526 | 0.0241 | 2.35E-10 | -1.07E-05 | 5.11E-05 | 0.83 | 0.01033 | 323.7356 |
| rs60600003 | G | T | 7 | 37382465 | 0.2128 | 0.0372 | 1.03E-08 | -5.49E-05 | 8.25E-05 | 0.51 | 0.008274 | 258.7529 |
| rs674451 | C | T | 6 | 1.38E+08 | 0.1307 | 0.0235 | 2.82E-08 | -0.00011 | 5.24E-05 | 0.035 | 0.007699 | 240.6349 |
| rs9481169 | T | G | 6 | 1.12E+08 | 0.2515 | 0.0422 | 2.47E-09 | 5.67E-05 | 8.82E-05 | 0.52 | 0.009116 | 285.322 |

Supplementary Table 27. Genetic instrumental tools used in Mendelian randomization analysis on psoriasis and bile duct cancer, based on FinnGen.

| SNP | Effect allele | Other allele | Chr | Pos | Exposure | | | Outcome | | | R^2^ | F |
| --- | --- | --- | --- | --- | --- | --- | --- | --- | --- | --- | --- | --- |
|  |  |  |  |  | beta | se | pval | beta | se | pval |  |  |
| rs10829130 | A | G | 10 | 27174346 | 0.1965 | 0.0359 | 4.24E-08 | -5.91E-05 | 0.000108 | 0.58 | 0.00756 | 218.1946 |
| rs12188300 | T | A | 5 | 1.59E+08 | 0.4331 | 0.0495 | 2.24E-18 | -5.57E-05 | 0.000122 | 0.649999 | 0.020821 | 609.0577 |
| rs12713428 | C | A | 2 | 61118113 | 0.1694 | 0.0261 | 8.11E-11 | -7.19E-05 | 8.46E-05 | 0.4 | 0.010724 | 310.4836 |
| rs13210419 | A | G | 6 | 31266977 | 1.1157 | 0.0511 | 1.10E-105 | 0.000204 | 0.000135 | 0.13 | 0.137296 | 4558.334 |
| rs1611309 | T | C | 6 | 29902063 | 0.241 | 0.0267 | 1.60E-19 | 6.05E-05 | 8.33E-05 | 0.47 | 0.027179 | 800.2138 |
| rs17728338 | A | G | 5 | 1.5E+08 | 0.3092 | 0.0439 | 1.76E-12 | -0.00032 | 0.000163 | 0.047 | 0.01299 | 376.9601 |
| rs2021511 | T | C | 16 | 11344903 | -0.1387 | 0.0254 | 4.75E-08 | -4.85E-05 | 7.93E-05 | 0.54 | 0.007519 | 217.0025 |
| rs28752856 | G | C | 6 | 31298421 | 0.833 | 0.0393 | 5.90E-100 | 0.000107 | 0.000112 | 0.34 | 0.136512 | 4528.209 |
| rs28998802 | A | G | 17 | 26124908 | 0.1672 | 0.0289 | 7.41E-09 | -2.93E-05 | 0.000103 | 0.780001 | 0.008434 | 243.6138 |
| rs4713605 | A | T | 6 | 32985992 | 0.1526 | 0.0241 | 2.35E-10 | 4.14E-05 | 7.37E-05 | 0.57 | 0.01033 | 298.9782 |
| rs60600003 | G | T | 7 | 37382465 | 0.2128 | 0.0372 | 1.03E-08 | -7.52E-05 | 0.000119 | 0.53 | 0.008274 | 238.965 |
| rs674451 | C | T | 6 | 1.38E+08 | 0.1307 | 0.0235 | 2.82E-08 | -0.00011 | 7.56E-05 | 0.13 | 0.007699 | 222.2325 |
| rs9481169 | T | G | 6 | 1.12E+08 | 0.2515 | 0.0422 | 2.47E-09 | -4.90E-05 | 0.000127 | 0.7 | 0.009116 | 263.5023 |

Supplementary Table 28. Genetic instrumental tools used in Mendelian randomization analysis on psoriasis and pancreatic cancer, based on FinnGen.

| SNP | Effect allele | Other allele | Chr | Pos | Exposure | | | Outcome | | | R2 | F |
| --- | --- | --- | --- | --- | --- | --- | --- | --- | --- | --- | --- | --- |
|  |  |  |  |  | beta | se | pval | beta | se | pval |  |  |
| rs10829130 | A | G | 10 | 27177245 | 0.1965 | 0.0359 | 4.24E-08 | 0.02401 | 0.069787 | 0.730321 | 0.00756 | 16.81645 |
| rs12713428 | C | A | 2 | 61133793 | 0.1694 | 0.0261 | 8.11E-11 | -0.03366 | 0.055562 | 0.544705 | 0.010724 | 23.92925 |
| rs2021511 | T | C | 16 | 11361202 | -0.1387 | 0.0254 | 4.75E-08 | 0.015086 | 0.0523 | 0.77332 | 0.007519 | 16.72458 |
| rs674451 | C | T | 6 | 1.38E+08 | 0.1307 | 0.0235 | 2.82E-08 | -0.01228 | 0.048915 | 0.80188 | 0.007699 | 17.12766 |

Supplementary Table 29. Genetic instrumental tools used in Mendelian randomization analysis on psoriasis and colorectal cancer, based on FinnGen.

| SNP | Effect allele | Other allele | Chr | Pos | Exposure | | | Outcome | | | R^2^ | F |
| --- | --- | --- | --- | --- | --- | --- | --- | --- | --- | --- | --- | --- |
|  |  |  |  |  | beta | se | pval | beta | se | pval |  |  |
| rs10829130 | A | G | 10 | 27174346 | 0.1965 | 0.0359 | 4.24E-08 | 3.79E-05 | 0.000424 | 0.93 | 0.007560279 | 221.3044492 |
| rs12188300 | T | A | 5 | 1.59E+08 | 0.4331 | 0.0495 | 2.24E-18 | -0.00026 | 0.000481 | 0.59 | 0.020821403 | 617.738344 |
| rs12713428 | C | A | 2 | 61118113 | 0.1694 | 0.0261 | 8.11E-11 | -0.00032 | 0.000333 | 0.34 | 0.010723733 | 314.9088586 |
| rs13210419 | A | G | 6 | 31266977 | 1.1157 | 0.0511 | 1.10E-105 | 0.0008 | 0.000533 | 0.13 | 0.137295926 | 4623.302256 |
| rs1611309 | T | C | 6 | 29902063 | 0.241 | 0.0267 | 1.60E-19 | 0.000413 | 0.000328 | 0.21 | 0.027178707 | 811.6189966 |
| rs17728338 | A | G | 5 | 1.5E+08 | 0.3092 | 0.0439 | 1.76E-12 | 0.000481 | 0.00064 | 0.450001 | 0.012989926 | 382.3328051 |
| rs2021511 | T | C | 16 | 11344903 | -0.1387 | 0.0254 | 4.75E-08 | -0.0001 | 0.000312 | 0.75 | 0.007519284 | 220.0953515 |
| rs28752856 | G | C | 6 | 31298421 | 0.833 | 0.0393 | 5.90E-100 | -0.00132 | 0.00044 | 0.0027 | 0.136512447 | 4592.748408 |
| rs28998802 | A | G | 17 | 26124908 | 0.1672 | 0.0289 | 7.41E-09 | 0.000464 | 0.000407 | 0.25 | 0.008433605 | 247.0859014 |
| rs4713605 | A | T | 6 | 32985992 | 0.1526 | 0.0241 | 2.35E-10 | -7.86E-07 | 0.00029 | 1 | 0.010330453 | 303.2394231 |
| rs60600003 | G | T | 7 | 37382465 | 0.2128 | 0.0372 | 1.03E-08 | -7.53E-05 | 0.000468 | 0.87 | 0.008274002 | 242.3708486 |
| rs674451 | C | T | 6 | 1.38E+08 | 0.1307 | 0.0235 | 2.82E-08 | -0.00054 | 0.000298 | 0.066999 | 0.007699112 | 225.399925 |
| rs9481169 | T | G | 6 | 1.12E+08 | 0.2515 | 0.0422 | 2.47E-09 | -0.00116 | 0.000501 | 0.021 | 0.009115845 | 267.2578847 |

Supplementary Table 30. Genetic instrumental tools used in Mendelian randomization analysis on psoriasis and kidney cancer, based on FinnGen.

| SNP | Effect allele | Other allele | Chr | Pos | Exposure | | | Outcome | | | R^2^ | F |
| --- | --- | --- | --- | --- | --- | --- | --- | --- | --- | --- | --- | --- |
|  |  |  |  |  | beta | se | se | pval | se | pval |  |  |
| rs4713605 | A | T | 6 | 32985992 | 0.1526 | 0.0241 | 0.0241 | 2.35E-10 | 0.000106 | 0.56 | 0.01033 | 2416.5 |
| rs674451 | C | T | 6 | 1.38E+08 | 0.1307 | 0.0235 | 0.0235 | 2.82E-08 | 0.000108 | 0.31 | 0.007699 | 1796.201 |

Supplementary Table 31. Genetic instrumental tools used in Mendelian randomization analysis on psoriasis and prostate cancer, based on FinnGen.

| SNP | Effect allele | Other allele | Chr | Pos | Exposure | | | Outcome | | | R^2^ | F |
| --- | --- | --- | --- | --- | --- | --- | --- | --- | --- | --- | --- | --- |
|  |  |  |  |  | beta | se | pval | beta | se | pval |  |  |
| rs10829130 | A | G | 10 | 27174346 | 0.1965 | 0.0359 | 4.24E-08 | -0.00108 | 0.001094 | 0.32 | 0.00756 | 107.0082 |
| rs12188300 | T | A | 5 | 1.59E+08 | 0.4331 | 0.0495 | 2.24E-18 | -0.00068 | 0.001237 | 0.58 | 0.020821 | 298.6975 |
| rs12713428 | C | A | 2 | 61118113 | 0.1694 | 0.0261 | 8.11E-11 | -0.00104 | 0.000857 | 0.23 | 0.010724 | 152.2692 |
| rs13210419 | A | G | 6 | 31266977 | 1.1157 | 0.0511 | 1.10E-105 | -0.001 | 0.001374 | 0.47 | 0.137296 | 2235.524 |
| rs1611309 | T | C | 6 | 29902063 | 0.241 | 0.0267 | 1.60E-19 | 0.001486 | 0.000845 | 0.079001 | 0.027179 | 392.4455 |
| rs17728338 | A | G | 5 | 1.5E+08 | 0.3092 | 0.0439 | 1.76E-12 | -0.00111 | 0.001648 | 0.5 | 0.01299 | 184.8709 |
| rs2021511 | T | C | 16 | 11344903 | -0.1387 | 0.0254 | 4.75E-08 | 0.00123 | 0.000804 | 0.13 | 0.007519 | 106.4236 |
| rs28752856 | G | C | 6 | 31298421 | 0.833 | 0.0393 | 5.9E-100 | 5.43E-05 | 0.001132 | 0.96 | 0.136512 | 2220.75 |
| rs28998802 | A | G | 17 | 26124908 | 0.1672 | 0.0289 | 7.41E-09 | -0.00016 | 0.001048 | 0.88 | 0.008434 | 119.4745 |
| rs4713605 | A | T | 6 | 32985992 | 0.1526 | 0.0241 | 2.35E-10 | -0.0000815 | 0.000748 | 0.91 | 0.01033 | 146.6266 |
| rs60600003 | G | T | 7 | 37382465 | 0.2128 | 0.0372 | 1.03E-08 | -0.00226 | 0.001204 | 0.06 | 0.008274 | 117.1946 |
| rs674451 | C | T | 6 | 1.38E+08 | 0.1307 | 0.0235 | 2.82E-08 | -0.00203 | 0.000766 | 0.008 | 0.007699 | 108.9885 |
| rs9481169 | T | G | 6 | 1.12E+08 | 0.2515 | 0.0422 | 2.47E-09 | -0.00236 | 0.001292 | 0.068 | 0.009116 | 129.2283 |

Supplementary Table 32. Genetic instrumental tools used in Mendelian randomization analysis on psoriasis and bladder cancer, based on FinnGen.

| SNP | Effect allele | Other allele | Chr | Pos | Exposure | | | Outcome | | | R^2^ | F |
| --- | --- | --- | --- | --- | --- | --- | --- | --- | --- | --- | --- | --- |
|  |  |  |  |  | beta | se | pval | beta | se | pval |  |  |
| rs10829130 | A | G | 10 | 27174346 | 0.1965 | 0.0359 | 4.24E-08 | -0.0000383 | 0.000205 | 0.85 | 0.00756 | 218.739 |
| rs12188300 | T | A | 5 | 1.59E+08 | 0.4331 | 0.0495 | 2.24E-18 | 0.000252 | 0.000232 | 0.28 | 0.020821 | 610.5772 |
| rs12713428 | C | A | 2 | 61118113 | 0.1694 | 0.0261 | 8.11E-11 | -0.00014 | 0.000161 | 0.39 | 0.010724 | 311.2583 |
| rs13210419 | A | G | 6 | 31266977 | 1.1157 | 0.0511 | 1.1E-105 | -0.0000712 | 0.000258 | 0.780001 | 0.137296 | 4569.707 |
| rs1611309 | T | C | 6 | 29902063 | 0.241 | 0.0267 | 1.60E-19 | 3.28E-05 | 0.000158 | 0.84 | 0.027179 | 802.2103 |
| rs17728338 | A | G | 5 | 1.5E+08 | 0.3092 | 0.0439 | 1.76E-12 | -0.00038 | 0.00031 | 0.22 | 0.01299 | 377.9006 |
| rs2021511 | T | C | 16 | 11344903 | -0.1387 | 0.0254 | 4.75E-08 | -0.00013 | 0.000151 | 0.4 | 0.007519 | 217.5439 |
| rs28752856 | G | C | 6 | 31298421 | 0.833 | 0.0393 | 5.90E-100 | -0.00017 | 0.000213 | 0.42 | 0.136512 | 4539.507 |
| rs28998802 | A | G | 17 | 26124908 | 0.1672 | 0.0289 | 7.41E-09 | -0.00013 | 0.000197 | 0.52 | 0.008434 | 244.2216 |
| rs4713605 | A | T | 6 | 32985992 | 0.1526 | 0.0241 | 2.35E-10 | 0.000137 | 0.00014 | 0.33 | 0.01033 | 299.7241 |
| rs60600003 | G | T | 7 | 37382465 | 0.2128 | 0.0372 | 1.03E-08 | 0.00017 | 0.000226 | 0.450001 | 0.008274 | 239.5612 |
| rs674451 | C | T | 6 | 1.38E+08 | 0.1307 | 0.0235 | 2.82E-08 | 3.33E-05 | 0.000144 | 0.82 | 0.007699 | 222.787 |
| rs9481169 | T | G | 6 | 1.12E+08 | 0.2515 | 0.0422 | 2.47E-09 | -6.68E-05 | 0.000242 | 0.780001 | 0.009116 | 264.1597 |

Supplementary Table 33. Genetic instrumental tools used in Mendelian randomization analysis on psoriasis and cervical carcinoma, based on FinnGen.

| SNP | Effect allele | Other allele | Chr | Pos | Exposure | | | Outcome | | | R^2^ | F |
| --- | --- | --- | --- | --- | --- | --- | --- | --- | --- | --- | --- | --- |
|  |  |  |  |  | beta | se | pval | beta | se | pval |  |  |
| rs10829130 | A | G | 10 | 27174346 | 0.1965 | 0.0359 | 4.24E-08 | 0.000218 | 0.000256 | 0.39 | 0.00756 | 116.6542 |
| rs12188300 | T | A | 5 | 1.59E+08 | 0.4331 | 0.0495 | 2.24E-18 | -0.00021 | 0.00029 | 0.46 | 0.020821 | 325.6229 |
| rs12713428 | C | A | 2 | 61118113 | 0.1694 | 0.0261 | 8.11E-11 | 0.000102 | 0.000201 | 0.61 | 0.010724 | 165.9951 |
| rs13210419 | A | G | 6 | 31266977 | 1.1157 | 0.0511 | 1.10E-105 | 0.000187 | 0.000321 | 0.56 | 0.137296 | 2437.04 |
| rs1611309 | T | C | 6 | 29902063 | 0.241 | 0.0267 | 1.60E-19 | 0.000373 | 0.000197 | 0.059 | 0.027179 | 427.8214 |
| rs17728338 | A | G | 5 | 1.5E+08 | 0.3092 | 0.0439 | 1.76E-12 | 0.000183 | 0.000386 | 0.64 | 0.01299 | 201.5357 |
| rs2021511 | T | C | 16 | 11344903 | -0.1387 | 0.0254 | 4.75E-08 | -0.00019 | 0.000188 | 0.32 | 0.007519 | 116.0169 |
| rs28752856 | G | C | 6 | 31298421 | 0.833 | 0.0393 | 5.90E-100 | 0.000122 | 0.000265 | 0.649999 | 0.136512 | 2420.934 |
| rs28998802 | A | G | 17 | 26124908 | 0.1672 | 0.0289 | 7.41E-09 | -0.0002 | 0.000245 | 0.42 | 0.008434 | 130.2442 |
| rs4713605 | A | T | 6 | 32985992 | 0.1526 | 0.0241 | 2.35E-10 | -1.24E-05 | 0.000175 | 0.94 | 0.01033 | 159.8439 |
| rs60600003 | G | T | 7 | 37382465 | 0.2128 | 0.0372 | 1.03E-08 | 0.000227 | 0.000283 | 0.42 | 0.008274 | 127.7588 |
| rs674451 | C | T | 6 | 1.38E+08 | 0.1307 | 0.0235 | 2.82E-08 | -0.00011 | 0.000179 | 0.54 | 0.007699 | 118.813 |
| rs9481169 | T | G | 6 | 1.12E+08 | 0.2515 | 0.0422 | 2.47E-09 | -0.0002 | 0.000302 | 0.51 | 0.009116 | 140.8773 |

Supplementary Table 34. Genetic instrumental tools used in Mendelian randomization analysis on psoriasis and covaria cancer, based on FinnGen.

| SNP | Effect allele | Other allele | Chr | Pos | Exposure | | | Outcome | | | R^2^ | F |
| --- | --- | --- | --- | --- | --- | --- | --- | --- | --- | --- | --- | --- |
|  |  |  |  |  | beta | se | pval | beta | se | pval |  |  |
| rs10829130 | A | G | 10 | 27174346 | 0.1965 | 0.0359 | 4.24E-08 | 0.000382 | 0.000374 | 0.31 | 0.00756 | 117.0381 |
| rs12188300 | T | A | 5 | 1.59E+08 | 0.4331 | 0.0495 | 2.24E-18 | -0.00055 | 0.000424 | 0.2 | 0.020821 | 326.6943 |
| rs12713428 | C | A | 2 | 61118113 | 0.1694 | 0.0261 | 8.11E-11 | -0.00028 | 0.000294 | 0.34 | 0.010724 | 166.5412 |
| rs13210419 | A | G | 6 | 31266977 | 1.1157 | 0.0511 | 1.10E-105 | -0.00058 | 0.00047 | 0.22 | 0.137296 | 2445.058 |
| rs1611309 | T | C | 6 | 29902063 | 0.241 | 0.0267 | 1.60E-19 | -0.00014 | 0.000289 | 0.630001 | 0.027179 | 429.2291 |
| rs17728338 | A | G | 5 | 1.5E+08 | 0.3092 | 0.0439 | 1.76E-12 | 0.000162 | 0.000565 | 0.77 | 0.01299 | 202.1988 |
| rs2021511 | T | C | 16 | 11344903 | -0.1387 | 0.0254 | 4.75E-08 | -0.00022 | 0.000275 | 0.42 | 0.007519 | 116.3986 |
| rs28752856 | G | C | 6 | 31298421 | 0.833 | 0.0393 | 5.90E-100 | -0.00073 | 0.000388 | 0.059 | 0.136512 | 2428.9 |
| rs28998802 | A | G | 17 | 26124908 | 0.1672 | 0.0289 | 7.41E-09 | 0.000347 | 0.000359 | 0.33 | 0.008434 | 130.6727 |
| rs4713605 | A | T | 6 | 32985992 | 0.1526 | 0.0241 | 2.35E-10 | 0.000246 | 0.000255 | 0.33 | 0.01033 | 160.3698 |
| rs60600003 | G | T | 7 | 37382465 | 0.2128 | 0.0372 | 1.03E-08 | 0.000312 | 0.000414 | 0.450001 | 0.008274 | 128.1791 |
| rs674451 | C | T | 6 | 1.38E+08 | 0.1307 | 0.0235 | 2.82E-08 | 3.40E-05 | 0.000263 | 0.9 | 0.007699 | 119.204 |
| rs9481169 | T | G | 6 | 1.12E+08 | 0.2515 | 0.0422 | 2.47E-09 | -0.00019 | 0.000442 | 0.68 | 0.009116 | 141.3408 |

Supplementary Table 35**.** Heterogeneity tests and MR-Egger intercept of SLE causally linked to cancer, based on Stuart PE et al and FinnGen.

| Database | Outcome | Heterogeneity | | | | | | MR-Egger | |
| --- | --- | --- | --- | --- | --- | --- | --- | --- | --- |
|  |  | MR-Egger | | | IVW | | | Intercept | P-value |
|  |  | Cochrane Q | Q_df | P-value | Cochrane Q | Q_df | P-value |  |  |
| Stuart PE | brain cancer | 41.06013 | 46 | 0.6788497 | 41.07684 | 47 | 0.7153496 | -5.256033e-06 | 0.8977039 |
| Stuart PE | head and neck cancer | 43.74754 | 47 | 0.6080721 | 43.86827 | 48 | 0.6427546 | -1.704857e-03 | 0.7298026 |
| Stuart PE | skin cancer | 98.34994 | 45 | **7.548606e-06** | 98.43328 | 46 | **1.114541e-05** | -2.758677e-05 | 0.8460013 |
| Stuart PE | melanoma | 19.61021 | 23 | 0.6652959 | 19.73038 | 24 | 0.7120010 | -1.83266e-05 | 0.732006 |
| Stuart PE | multiple myeloma | 39.96044 | 46 | 0.7221313 | 39.96342 | 47 | 0.7567216 | -2.004122e-06 | 0.9566861 |
| Stuart PE | breast cancer | 202.5452 | 45 | **7.262295e-022** | 202.8899 | 46 | **1.361500e-21** | -0.00149014 | 0.7832485 |
| Stuart PE | lung cancer | 72.70116 | 47 | **0.009479515** | 75.11301 | 48 | **0.007429417** | 0.0001177444 | 0.2179635 |
| Stuart PE | esophageal cancer | 41.06013 | 46 | 0.6788497 | 41.07684 | 47 | 0.7153496 | -5.256033e-06 | 0.8977039 |
| Stuart PE | liver cancer | 46.19232 | 42 | 0.3031837 | 46.52047 | 43 | 0.3295263 | -1.153087e-05 | 0.5877957 |
| Stuart PE | bile duct cancer | 45.63697 | 46 | 0.4873662 | 45.70798 | 47 | 0.5261178 | -7.463319e-06 | 0.7910725 |
| Stuart PE | pancreatic cancer | 16.47427 | 20 | 0.6868002 | 16.77498 | 21 | 0.7246531 | 0.01711607 | 1.5895049 |
| Stuart PE | colorectal cancer | 76.50725 | 47 | **0.004191791** | 76.93641 | 48 | **0.005035148** | -7.14028e-05 | 0.6100344 |
| Stuart PE | kidney cancer | 28.36423 | 24 | 0.2449776 | 28.42405 | 25 | 0.2887051 | 1.202692e-05 | 0.8238969 |
| Stuart PE | prostate cancer | 49.73632 | 47 | 0.3648832 | 49.74061 | 48 | 0.4038270 | 1.840317e-05 | 0.9495011 |
| Stuart PE | bladder cancer | 53.93172 | 47 | 0.2264544 | 53.95899 | 48 | 0.2571585 | 8.705724e-06 | 0.8781437 |
| Stuart PE | cervical carcinoma | 49.93877 | 46 | 0.3196877 | 53.42871 | 47 | 0.2409889 | 0.0001240273 | 0.0795569 |
| Stuart PE | ovarian cancer | 30.91442 | 47 | 0.9661774 | 31.35286 | 46 | 0.9698079 | 6.36126e-05 | 0.5111129 |
| FinnGen | brain cancer | 12.67968 | 10 | 0.2421367 | 12.68181 | 11 | 0.3146297 | 2.607187e-06 | 0.9681091 |
| FinnGen | head and neck cancer | 11.17727 | 10 | 0.3438752 | 11.78184 | 11 | 0.3802928 | 5.91855e-05 | 0.4789546 |
| FinnGen | skin cancer | 2.029962 | 8 | 0.9800795 | 2.899952 | 9 | 0.9681296 | -0.000136656 | 0.378249 |
| FinnGen | melanoma | 0.00151801 | 1 | 0.968921 | NA | NA | NA | NA | NA |
| FinnGen | multiple myeloma | 4.380955 | 10 | 0.9285296 | 4.586007 | 11 | 0.9495493 | 2.549289e-05 | 0.6603393 |
| FinnGen | breast cancer | 6.393274 | 11 | 0.8458785 | 6.689573 | 12 | 0.8774278 | -0.002131987 | 0.5970733 |
| FinnGen | lung cancer | 16.76142 | 10 | 0.07981257 | 24.57956 | 11 | 0.05049920 | -0.0003288272 | 0.05613663 |
| FinnGen | esophageal cancer | 5.029252 | 10 | 0.8892154 | 5.977605 | 10 | 0.8748543 | 6.073704e-05 | 0.3531064 |
| FinnGen | liver cancer | 8.038069 | 9 | 0.5303144 | 11.708540 | 10 | 0.3050363 | -5.718359e-05 | 0.08763742 |
| FinnGen | bile duct cancer | 9.027451 | 10 | 0.5295004 | 11.410260 | 11 | 0.4095601 | -6.632317e-05 | 0.1537076 |
| FinnGen | pancreatic cancer | 0.3543625 | 2 | 0.8376279 | 0.4610346 | 3 | 0.9273646 | -0.05800688 | 0.774977 |
| FinnGen | colorectal cancer | 13.20689 | 10 | 0.2123332 | 15.20639 | 11 | 0.1732421 | -0.0002390524 | 0.2466914 |
| FinnGen | kidney cancer | 1.336045 | 1 | 0.2477326 | NA | NA | NA | NA | NA |
| FinnGen | prostate cancer | 14.59005 | 10 | 0.14773790 | 18.63250 | 11 | 0.06801775 | -0.0008762002 | 0.1269781 |
| FinnGen | bladder cancer | 6.090287 | 10 | 0.8076215 | 6.319404 | 11 | 0.8512254 | 3.914655e-05 | 0.6424728 |
| FinnGen | cervical carcinoma | 7.689399 | 10 | 0.6591465 | 7.839316 | 11 | 0.7276213 | 3.943334e-05 | 0.7067267 |
| FinnGen | ovarian cancer | 5.449480 | 10 | 0.8592043 | 7.909109 | 11 | 0.7214219 | 0.0002337881 | 0.1478775 |

Supplementary Table 36. Results of Mendelian randomization on psoriasis and cancer, based on Stuart PE et al and FinnGen.

| Database | Methods | Disease | beta | se | P value | OR | 95% CI |
| --- | --- | --- | --- | --- | --- | --- | --- |
| Stuart PE | MR Egger  weighted median  Inverse variance weighted  Simple mode  weighted mode | brain cancer | 1.256535e-04 | 0.0002463561 | 0.6124564 | 1.0001257 | 0.9996429-1.000609 |
|  |  |  | 5.146643e-05 | 0.0001637040 | 0.7532270 | 1.0000525 | 0.9997306-1.000372 |
|  |  |  | 9.680220e-05 | 0.0001043208 | 0.3534447 | 1.0000968 | 0.9998923-1.000301 |
|  |  |  | -1.127119e-04 | 0.0003005294 | 0.7093146 | 0.9998873 | 0.9992985-1.000476 |
|  |  |  | 3.091638e-05 | 0.0002086488 | 0.8828391 | 1.0000309 | 0.9996220-1.000440 |
| Stuart PE | MR Egger  weighted median  Inverse variance weighted  Simple mode  weighted mode | head and neck cancer | 0.0003769763  0.0003225843  0.0002841125  0.0005787668  0.0003772360 | 0.0002957219  0.0001985380  0.0001265619  0.0003420475  0.0002259384 | 0.20865817  0.10420549  0.02477772  0.09711634  0.10150036 | 1.000377  1.000323  1.000284  1.000579  1.000377 | 0.9997974-1.000957  0.9999335-1.000712  1.0000361-1.000532  0.9999084-1.001250  0.9999344-1.000820 |
| Stuart PE | MR Egger  weighted median  Inverse variance weighted  Simple mode  weighted mode | skin cancer | 0.0003900549  -0.0001369913  0.0002364893  -0.0007844438  -0.0003062304 | 0.0008637792  0.0003982116  0.0003535150  0.0008611845  0.0005579737 | 0.6537493  0.7309339  0.5035175  0.3671024  0.5857792 | 1.0003901  0.9998630  1.0002365  0.9992159  0.9996938 | 0.9986979-1.002085  0.9990829-1.000644  0.9995437-1.000930  0.9975307-1.000904  0.99866011-1.000788 |
| Stuart PE | MR Egger  weighted median  Inverse variance weighted  Simple mode  weighted mode | melanoma | -1.607112e-04  -2.45278e-04  -2.80542e-04  5.078255e-05  -2.233447e-04 | 0.0003743318  0.0002240891  0.0001436016  0.0003760081  0.0002603610 | 0.671677932  0.27371120  0.05074441  0.89369308  0.39947380 | 0.9998393  0.9997548  0.9997195  1.0000508  0.9997767 | 0.9991060-1.000573  0.9993157-1.000194  0.9994382-1.000001  0.9993140-1.000788  0.9992666-1.000287 |
| Stuart PE | MR Egger  weighted median  Inverse variance weighted  Simple mode  weighted mode | multiple myeloma | 5.392417e-05  -8.06038e-05  4.292334e-05  -7.004704e-06  -5.944430e-05 | 0.0002223674  0.0001429742  0.0000941648  0.0002435957  0.00019700972 | 0.8094705  0.5729140  0.6485107  0.9770511  0.7642833 | 1.0000539  0.5729140  0.6485107  0.9770511  0.7642833 | 0.9996182-1.000490  0.9996392-1.000200  0.9998584-1.000228  0.9995156-1.000471  0.9995543-1.000327 |
| Stuart PE | MR Egger  weighted median  Inverse variance weighted  Simple mode  weighted mode | breast cancer | -0.017695654  -0.023074852  -0.026075407  0.003940079  -0.014322115 | 0.03329585  0.01220176  0.01370616  0.02416881  0.01448811 | 0.59770893  0.05860984  0.05711124  0.87121420  0.32805828 | 0.9824600  0.9771893  0.9742616  1.0039479  0.9857800 | 0.9203921-1.048714  0.9540967-1.000841  0.9484374-1.000789  0.9574988-1.052650  0.9581808-1.014174 |
| Stuart PE | MR Egger  weighted median  Inverse variance weighted  Simple mode  weighted mode | lung cancer | -3.449221e-04  -3.071025e-05  3.964571e-04  -5.588771e-04  -8.006630e-04 | 0.0005683164  0.0003433685  0.0002446299  0.0009654493  0.0005443445 | 0.5426254  0.9287545  0.2255661  0.5217462  0.1478504 | 0.9996551  0.9999693  1.0002965  0.9994413  0.9991997 | 0.9985422-1.000769  0.9992965-1.000643  0.9998170-1.000776  0.9977464-1.001139  0.9981342-1.000266 |
| Stuart PE | MR Egger  weighted median  Inverse variance weighted  Simple mode  weighted mode | esophageal cancer | 1.256535e-04  5.146643e-05  9.680220e-05  -1.127119e-04  3.091638e-05 | 0.0002463561  0.0001637040  0.0001043208  0.0003005294  0.0002086488 | 0.6124564  0.7532270  0.3534447  0.7093146  0.8828391 | 1.0001257  1.0000515  1.0000968  0.9998873  1.0000309 | 0.9996429-1.000609  0.9997306-1.000372  0.9998923-1.000301  0.9992985-1.000476  0.9996220-1.000440 |
| Stuart PE | MR Egger  weighted median  Inverse variance weighted  Simple mode  weighted mode | liver cancer | -1.722598e-05  -8.554084e-05  -8.265509e-05  -1.282307e-04  -8.969770e-05 | 1.316006e-04  8.574026e-05  5.405943e-05  1.461788e-04  9.894622e-05 | 0.8964826  0.3184374  0.1262726  0.3852430  0.3697076 | 0.9999828  0.9999145  0.9999173  0.9998718  0.9999103 | 0.9997249-1.000241  0.9997464-1.000083  0.9998114-1.000023  0.9995853-1.000158  0.9997164-1.000104 |
| Stuart PE | MR Egger  weighted median  Inverse variance weighted  Simple mode  weighted mode | bile duct cancer | 1.026651e-05  -1.377713e-04  -3.070112e-05  2.38137e-04  -1.891523e-04 | 1.697127e-04  1.108538e-04  7.186641e-05  2.217194e-04  1.540046e-04 | 0.9520247  0.2139342  0.6692358  0.2882839  0.2254780 | 1.0000103  0.9998622  0.9999693  1.0002382  0.9998109 | 0.9996777-1.000343  0.9996450-1.000080  0.9998285-1.000110  0.9998036-1.000673  0.9995091-1.000113 |
| Stuart PE | MR Egger  weighted median  Inverse variance weighted  Simple mode  weighted mode | pancreatic cancer | -0.06055160  0.02339400  0.04707955  0.04357920  0.03479049 | 0.20942649  0.10201271  0.07304921  0.21344670  0.17949187 | 0.7754572  0.8186168  0.5192572  0.8401871  0.8481734 | 0.9412452  1.0236698  1.0482054  1.0445427  1.0354028 | 0.6243604-1.418960  0.8381561-1.250244  0.9083760-1.209559  0.6874430-1.5787142  0.7283209-1.471960 |
| Stuart PE | MR Egger  weighted median  Inverse variance weighted  Simple mode  weighted mode | colorectal cancer | 6.850669e-04  5.797045e-05  3.961203e-04  3.953156e-04  2.520779e-05 | 0.0008381335  0.0004852998  0.0003559323  00010968359  0.0008398425 | 0.4178402  0.9049166  0.4054333  0.7201184  0.9761796 | 1.000685  1.000058  1.000296  1.000395  1.000025 | 0.9990428-1.002331  0.9991072-1.001010  0.9995986-1.000994  0.9982471-1.0002548  0.9983804-1.001673 |
| Stuart PE | MR Egger  weighted median  Inverse variance weighted  Simple mode  weighted mode | kidney cancer | -1.151062e-04  1.998616e-05  -4.237458e-05  2.581111e-04  5.926268e-05 | 0.000355022  0.0001901125  0.0001450651  0.0003123071  0.0002128228 | 0.7489057  0.9162742  0.7702046  0.4163583  0.7829494 | 0.9998849  1.0000200  0.9999576  1.0002581  0.0000593 | 0.9991884-1.000582  0.9996470-1.000393  0.9996734-1.000242  0.9996461-1.000871  0.9996422-1.000477 |
| Stuart PE | MR Egger  weighted median  Inverse variance weighted  Simple mode  weighted mode | prostate cancer | -0.001881135  -0.001052856  -0.001780895  -0.001666966  -0.001289182 | 0.0017418709  0.0010947151  0.0007376359  0.0021869776  0.0016758099 | 0.28567476  0.33616870  0.01576428  0.44965593  0.44576079 | 0.9981206  0.9989477  0.9982207  0.9983344  0.9987116 | 0.9947188-1.0015341  0.9968066-1.0010934  0.9967785-0.9996649  0.9940642-1.0026229  0.9954347-1.0019994 |
| Stuart PE | MR Egger  weighted median  Inverse variance weighted  Simple mode  weighted mode | bladder cancer | 2.161309e-05  -3.364630e-05  6.903385e-05  -4.248300e-04  -2.588211e-05 | 0.0003403521  0.0002097375  0.0001441720  0.0004045524  0.0002717253 | 0.9496360  0.8725494  0.6320598  0.2989199  0.9245120 | 1.0000216  0.9999664  1.0000690  0.9995753  0.9999741 | 0.9993547-1.000689  0.9995554-1.000378  0.9997865-1.000352  0.9987830-1.000368  0.9994417-1.000507 |
| Stuart PE | MR Egger  weighted median  Inverse variance weighted  Simple mode  weighted mode | cervical carcinoma | -8.770601e-04  -2.322672e-04  -1.961429e-04  -5.133399e-06  -4.268647e-04 | 0.0004192030  0.0002474874  0.0001816242  0.000489330  0.0003330060 | 0.041966674  0.34798687  0.28016977  0.99168446  0.20618179 | 0.9991233  0.9997678  0.9998029  0.9999949  0.9995732 | 0.9983027-0.9999446  0.9992829-1.0002528  0.9994480-1.0001599  0.9990351-1.0009556  0.9989210-1.0002259 |
| Stuart PE | MR Egger  weighted median  Inverse variance weighted  Simple mode  weighted mode | ovarian cancer | -1.007490e-04  6.109446e-05  6.547224e-05  1.961892e-04  5.168990e-05 | 2.231030e-04  1.447953e-04  9.447512e-05  2.804571e-04  1.706032e-04 | 0.6536932  0.6730711  0.4883030  0.4876664  0.7632397 | 0.9998993  1.0000611  1.0000655  1.0001962  1.0000517 | 0.9994621-1.000337  0.9997773-1.000345  0.9998803-1.000251  0.9996466-1.000746  0.9997173-1.000386 |
| FinnGen | MR Egger  weighted median  Inverse variance weighted  Simple mode  weighted mode | brain cancer | -8.916371e-07  -1.213881e-05  5.550577e-06  1.475179e-04  4.542366e-06 | 0.0002073197  0.0001488066  0.0001289327  0.0002704395  0.0001452990 | 0.9966531  0.9349852  0.9656615  0.5963147  0.9756205 | 0.9999991  0.9999879  1.0000056  1.0001475  1.0000045 | 0.9995928-1.000406  0.9996962-1.000280  0.9997529-1.000258  0.9996175-1.000678  0.9997198-1.000289 |
| FinnGen | MR Egger  weighted median  Inverse variance weighted  Simple mode  weighted mode | head and neck cancer | 0.0002252956  0.0002944165  0.0003715310  0.0009811576  0.0003135467 | 0.0002623041  0.0001948625  0.0001674703  0.0004221952  0.0002091108 | 0.41050618  0.13081553  0.02652152  0.04029690  0.16190407 | 1.000225  1.000294  1.000372  1.000982  1.000314 | 0.9997112-1.000740  0.9999125-1.000677  1.0000433-1.000700  1.0001537-1.001810  0.9999037-1.0000724 |
| FinnGen | MR Egger  weighted median  Inverse variance weighted  Simple mode  weighted mode | skin cancer | 1.904035e-04  4.725055e-05  -1.257507e-04  -7.199883e-04  5.174328e-05 | 0.0004658148  0.0003757816  0.0003195200  0.0007613179  0.0003719551 | 0.6934437  0.8999382  0.6939051  0.3689914  0.8924251 | 1.0001904  1.0000473  0.9998743  0.9992803  1.0000517 | 0.9992777-1.001104  0.9993110-1.000784  0.9992483-1.000501  0.9977903-1.000772  0.9993229-1.000781 |
| FinnGen | Inverse variance weighted | melanoma | -0.0008317943 | 0.0005142235 | 0.1057545 | 0.9991686 | 0.998162-1.000176 |
| FinnGen | MR Egger  weighted median  Inverse variance weighted  Simple mode  weighted mode | multiple myeloma | -2.764187e-05  -4.523864e-06  3.534778e-05  4.305488e-05  -9.696664e-06 | 0.0001835040  0.0001424281  0.0001196830  0.0002710308  0.0001532297 | 0.8832599  0.9746615  0.7677303  0.8766613  0.9506773 | 0.9999724  0.9999955  1.000035  1.0000431  0.9999903 | 0.9996128-1.000332  0.9997164-1.000275  0.9998008-1.000270  0.9995120-1.000574  0.9996900-1.000291 |
| FinnGen | MR Egger  weighted median  Inverse variance weighted  Simple mode  weighted mode | breast cancer | 0.018779765  0.017081948  0.013816665  0.009580158  0.018051858 | 0.012389912  0.09615009  0.008389065  0.018642343  0.010296099 | 0.15778393  0.07563540  0.09956112  0.61665817  0.10503750 | 1.018957  1.017229  1.013913  1.009626  1.018216 | 0.9945107-1.044005  0.9982381-1.036581  0.9973775-1.030722  0.9734013-1.047199  0.9978738-1.038972 |
| FinnGen | MR Egger  weighted median  Inverse variance weighted  Simple mode  weighted mode | lung cancer | 0.0009623620  0.0006273589  0.0001501679  -0.0011384732  0.0009537268 | 0.0004961549  0.0003667576  0.0003736806  0.0008106269  0.0003396646 | 0.08112927  0.08716335  0.68778592  0.18779080  0.01703394 | 1.0009628  1.0006276  1.0001502  0.9988622  1.0009542 | 0.9999899-1.001937  0.9999085-1.001347  0.9994179-1.000883  0.9972764-1.000450  1.0002880-1.001621 |
| FinnGen | MR Egger  weighted median  Inverse variance weighted  Simple mode  weighted mode | esophageal cancer | -1.561620e-04  -5.001501e-05  -6.085660e-06  -6.973070e-05  -4.935772e-05 | 0.0002032992  0.0001637938  0.0001325936  0.0002818095  0.0001649907 | 0.4601591  0.7600969  0.9633923  0.8091261  0.7703984 | 0.9998439  0.9999500  0.9999939  0.9999303  0.9999506 | 0.9994455-1.000242  0.9996290-1.000271  0.9997341-1.000254  0.9993781-1.000483  0.9996273-1.000274 |
| FinnGen | MR Egger  weighted median  Inverse variance weighted  Simple mode  weighted mode | liver cancer | 9.263963e-05  2.607188e-05  -4.729459e-05  -1.482369e-04  4.028042e-05 | 9.731221e-05  7.911666e-05  6.957881e-05  1.734884e-04  8.139758e-05 | 0.3659702  0.7417497  0.4966774  0.4128578  0.6313904 | 1.0000926  1.0000261  0.9999527  0.9998518  1.0000403 | 0.9999019-1.000283  0.9998710-1.000181  0.9998163-1.000089  0.9995118-1.000192  0.9998807-1.000200 |
| FinnGen | MR Egger  weighted median  Inverse variance weighted  Simple mode  weighted mode | bile duct cancer | 1.947538e-04  1.050377e-04  3.089383e-05  -1.942240e-04  1.610794e-04 | 1.400387e-04  1.106549e-04  9.302543e-05  2.489623e-04  1.119031e-04 | 0.1944817  0.3425001  0.7398130  0.4517725  0.1778663 | 1.0001948  1.0001050  1.0000309  0.9998058  1.0001611 | 0.9999203-1.000469  0.9998882-1.000322  0.9998486-1.000213  0.9993180-1.000294  0.9999418-1.000380 |
| FinnGen | MR Egger  weighted median  Inverse variance weighted  Simple mode  weighted mode | pancreatic cancer | 0.29683811  -0.10266659  -0.07369627  -0.11388657  -0.12013684 | 1.1484478  0.2031133  0.1784677  0.2686931  0.2763523 | 0.8202129  0.6132325  0.6796513  0.7002276  0.6931353 | 1.3455974  0.9024278  0.9289538  0.8923592  0.8867991 | 0.1416892-12.778906  0.6060647-1.343711  0.6547558-1.317980  0.5270157-1.510970  0.5159284-1.524267 |
| FinnGen | MR Egger  weighted median  Inverse variance weighted  Simple mode  weighted mode | colorectal cancer | 0.0008592267  0.0005989399  0.0002687801  0.0003539267  0.0006502322 | 0.0006331099  0.0004640199  0.0004225220  0.0008471174  0.0004587178 | 0.2045726  0.1967858  0.5246899  0.6841317  0.1840351 | 1.000860  1.000599  1.000269  1.000354  1.000650 | 0.9996184-1.002102  0.9996895-1.001510  0.9994408-1.001089  0.9986944-1.002016  0.9997512-1.001551 |
| FinnGen | Inverse variance weighted | kidney cancer | 0.0001054642 | 0.0006145701 | 0.8637469 | 1.000105 | 0.9989015-1.001311 |
| FinnGen | MR Egger  weighted median  Inverse variance weighted  Simple mode  weighted mode | prostate cancer | 0.0002124660  -0.0009188399  -0.0019543620  -0.0029931638  -0.0011756505 | 0.001716846  0.001107773  0.001206100  0.002803070  0.001126869 | 0.9039624  0.4068511  0.1051469  0.3084777  0.3192006 | 1.0002125  0.9990816  0.9980475  0.9970113  0.9988250 | 0.9968524-1.003584  0.9969147-1.001253  0.9956910-1.000410  0.9915487-1.002504  0.9966214-1.001034 |
| FinnGen | MR Egger  weighted median  Inverse variance weighted  Simple mode  weighted mode | bladder cancer | -7.457440e-05  -1.971909e-05  2.215209e-05  9.344123e-05  -2.041094e-05 | 0.0002665787  0.0002097561  0.0001738652  0.0003947750  0.0002198083 | 0.7853732  0.9251015  0.8986163  0.8172435  0.9276861 | 0.9999254  0.9999803  1.0000222  1.0000934  0.9999796 | 0.9994031-1.000448  0.9995693-1.000391  0.9996814-1.000363  0.9993199-1.000868  0.9995489-1.000410 |
| FinnGen | MR Egger  weighted median  Inverse variance weighted  Simple mode  weighted mode | cervical carcinoma | 0.0001093924  0.0001630732  0.0002067372  0.0008894342  0.0001421343 | 0.0003317525  0.0002662490  0.0002164523  0.0005726896  0.0002760148 | 0.7483987  0.5402178  0.3395187  0.1486839  0.6167761 | 1.000109  1.000163  1.000207  1.000890  1.000142 | 0.9994593-1.000760  0.9996413-1.000685  0.9997825-1.000631  0.9997670-1.002014  0.9996012-1.000683 |
| FinnGen | MR Egger  weighted median  Inverse variance weighted  Simple mode  weighted mode | ovarian cancer | -0.0008653940  -0.0005256838  -0.0002880824  0.0015687521  -0.005787914 | 0.0004857043  0.0003987378  0.0003168675  0.0008312084  0.0004102291 | 0.10513109  0.18737996  0.36326711  0.08577069  0.18592484 | 0.9991350  0.9994745  0.9997120  1.0015700  0.9994214 | 0.9981843-1.000087  0.9986936-1.000256  0.9990913-1.000333  0.9999396-1.003203  0.9986181-1.000225 |

Supplementary Figure 1. Analysis of psoriasis and cancers using the leave-one-out method, based on Stuart PE et al.


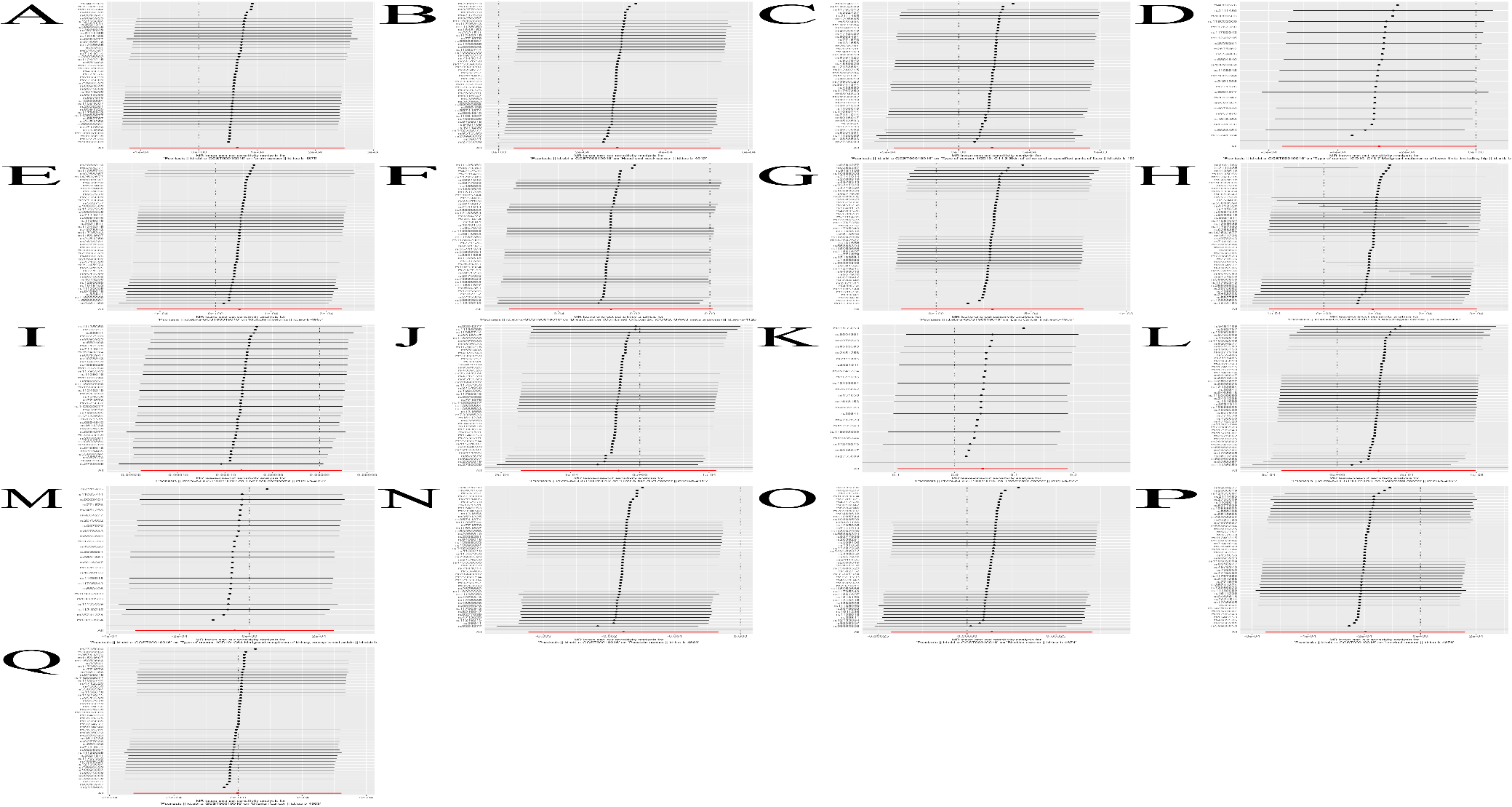


Supplementary Figure 2. Analysis of psoriasis and cancers using the leave-one-out method, based on FinnGen.


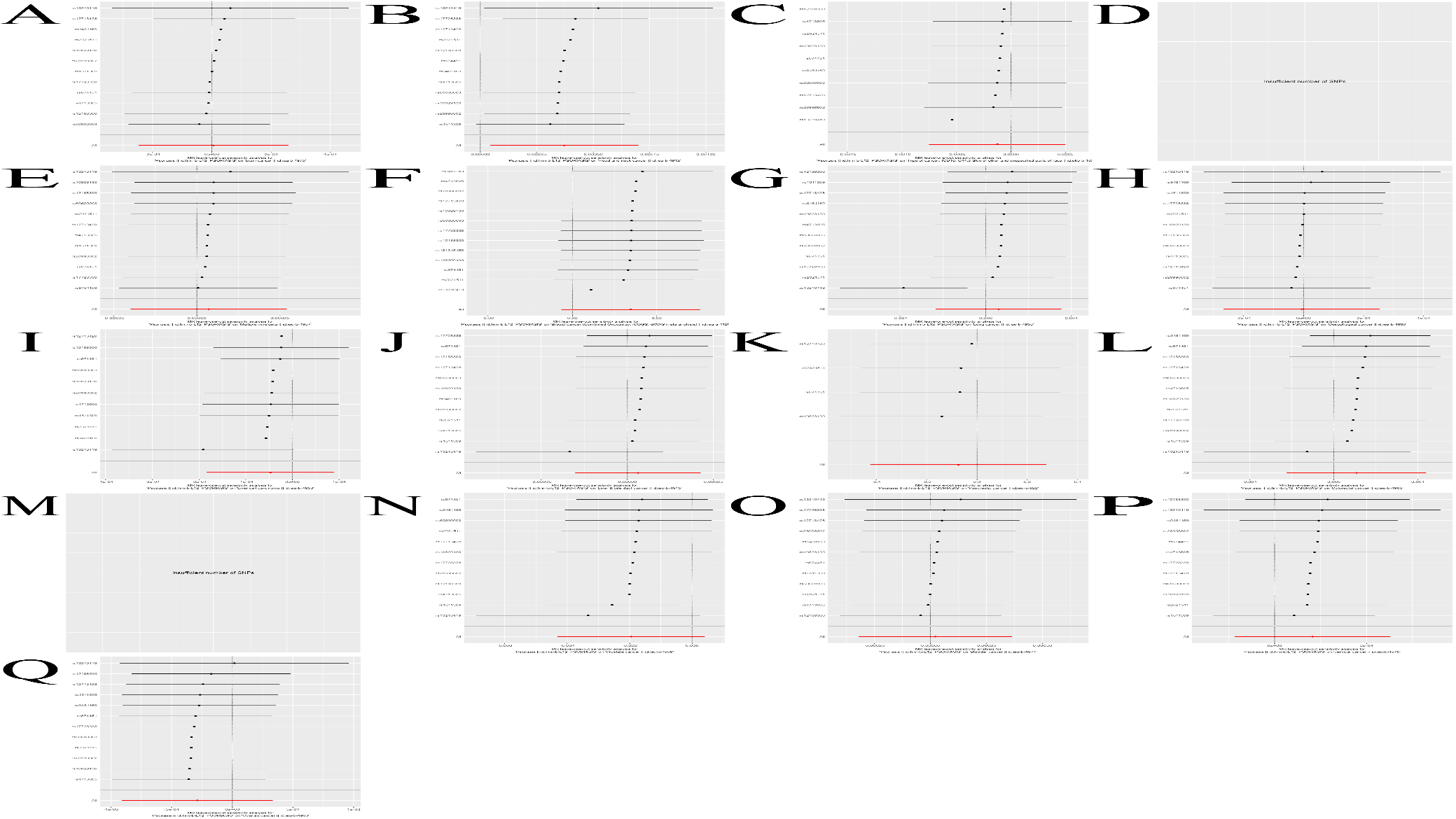

Supplement: Supplementary file 1 [file DataSheet_1.docx]
